# From Big Data to the Clinic: Methodological and Statistical Enhancements to Implement the UK Biobank Imaging Framework in a Memory Clinic

**DOI:** 10.1002/hbm.70151

**Published:** 2025-02-19

**Authors:** Grace Gillis, Gaurav Bhalerao, Jasmine Blane, Robert Mitchell, Pieter M. Pretorius, Celeste McCracken, Thomas E. Nichols, Stephen M. Smith, Karla L. Miller, Fidel Alfaro‐Almagro, Vanessa Raymont, Lola Martos, Clare E. Mackay, Ludovica Griffanti

**Affiliations:** ^1^ Department of Psychiatry, Oxford Centre for Human Brain Activity, Wellcome Centre for Integrative Neuroimaging University of Oxford Oxford UK; ^2^ Oxford Health NHS Foundation Trust Oxford UK; ^3^ Nuffield Department of Clinical Neurosciences, Wellcome Centre for Integrative Neuroimaging, FMRIB University of Oxford Oxford UK; ^4^ Department of Neuroradiology Oxford University Hospitals NHS Foundation Trust Oxford UK; ^5^ Division of Cardiovascular Medicine, Radcliffe Department of Medicine University of Oxford Oxford UK

## Abstract

The analysis tools and statistical methods used in large neuroimaging research studies differ from those applied in clinical contexts, making it unclear whether these techniques can be translated to a memory clinic setting. The Oxford Brain Health Clinic (OBHC) was established in 2020 to bridge this gap between research studies and memory clinics. We optimised the UK Biobank imaging framework for the memory clinic setting by integrating enhanced quality control (QC) processes (MRIQC, QUAD, and DSE decomposition) and supplementary dementia‐informed analyses (lobar volumes, NBM volumes, WMH classification, PSMD, cortical diffusion MRI metrics, and tract volumes) into the analysis pipeline. We explored associations between resultant imaging‐derived phenotypes (IDPs) and clinical phenotypes in the OBHC patient population (*N* = 213), applying hierarchical FDR correction to account for multiple testing. 14%–24% of scans were flagged by automated QC tools, but upon visual inspection, only 0%–2.4% of outputs were excluded. The pipeline successfully generated 5683 IDPs aligned with UK Biobank and 110 IDPs targeted towards dementia‐related changes. We replicated established associations and found novel associations between brain metrics and age, cognition, and dementia‐related diagnoses. The imaging protocol is feasible, acceptable, and yields high‐quality data that is usable for both clinical and research purposes. We validated the use of this methodology in a real‐world memory clinic population, which demonstrates the potential of this enhanced pipeline to bridge the gap between big data studies and clinical settings.

## Graphical abstract

We optimised the UK Biobank imaging framework for memory clinic use by integrating enhanced quality control (QC) and supplementary analyses targeted towards dementia‐related changes. These analyses capture established and novel associations between brain metrics and dementia‐related phenotypes, creating a translational bridge between big data analytics and real‐world clinical populations.

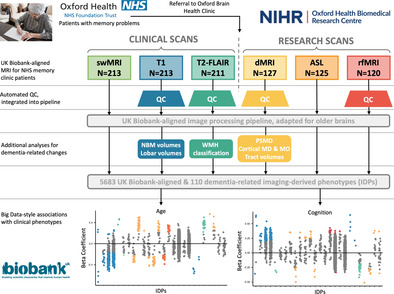

**Summary**

- The imaging methods, analysis techniques, and population characteristics in research studies often differ to those in traditional clinical settings.
- To bridge this gap, we optimised the UK Biobank imaging framework for memory clinic use by integrating enhanced quality control (QC) and supplementary analyses targeted towards dementia‐related changes.
- We generated 5683 imaging‐derived phenotypes (IDPs) aligned with UK Biobank and 110 supplementary dementia‐informed IDPs that captured both established and novel associations between brain metrics and dementia‐related clinical phenotypes, highlighting the value of integrating UK Biobank‐aligned imaging and analyses in a real‐world memory clinic population.

## 1 Introduction

State‐of‐the‐art neuroimaging is included in large population studies like UK Biobank to assess baseline ‘brain health’ and to predict the risk of diseases and disorders such as Alzheimer's Disease (AD) (Harms et al. 2018; Miller et al. 2016). Major advances in image analysis methodology and statistical methods have been developed within the UK Biobank imaging substudy (Alfaro‐Almagro et al. 2018, 2021), meaning that well‐powered conclusions can be drawn about the relationship between brain structure and function and a range of health and lifestyle factors. However, these advances widen the (already substantial) gap between neuroimaging methodology available in research settings and those available in clinical practice.

Structural brain imaging is included in the diagnostic guidelines for dementia, typically as a computerised tomography (CT) scan used primarily for ruling out alternative causes of cognitive impairment (Jack et al. 2016; National Institute for Health and Care Excellence (NICE) 2018). Compared to CT, structural magnetic resonance imaging (MRI) offers higher resolution and contrast, with greater sensitivity to brain changes like atrophy and white matter hyperintensities, both of which are hallmarks of AD and other forms of dementia (Harper et al. 2015). There are automated analysis tools to quantify these structural changes, including commercial software (Pemberton et al. 2021), but standard radiology practice continues to rely on qualitative or semi‐quantitative methods using visual rating scales (Fazekas et al. 1987; Harper et al. 2015; Scheltens et al. 1995). Advanced MR imaging and analysis have also revealed differences in cerebral blood flow and structural and functional connectivity in patients with cognitive impairment (Liu, Mazumdar, and Bath 2023; Penalba‐Sánchez et al. 2023; Teipel and Grothe 2023; Xiao et al. 2023), but these methods are currently not recommended for memory clinic use since their diagnostic and prognostic value is less established (Egle et al. 2022; Haidar et al. 2023; Vemuri, Jones, and Jack 2012). Thus, our understanding of the links between brain and phenotypic changes is constrained by the data currently available (i.e., cohort studies rather than real‐world clinical populations).

Participants in cohort studies, however, tend to be younger and healthier than patients with memory problems, making it difficult to (i) draw conclusions about dementia‐related changes and (ii) test the performance of analysis tools in brains representing more advanced disease stages. Dementia cohort studies have been established to address these limitations (e.g., ADNI—Petersen et al. 2010). However, these cohorts are often overused, which limits generalisability (Borchert et al. 2023), and use strict inclusion/exclusion criteria, making the data poorly representative of the overall memory clinic patient population (Langbaum et al. 2023; Lim et al. 2023). Methodological differences further widen the gap between large cohorts and smaller clinical studies. For example, while smaller clinical studies can employ visual inspection to control the quality and accuracy of raw and processed MRI data, big data studies must rely on automated quality control (QC) tools. Moreover, differences in study design (e.g., data‐ vs. hypothesis‐driven) and statistical power also require distinct, context‐dependent statistical approaches.

To bridge the technological, methodological, and population differences between research studies and memory clinics, the Oxford Brain Health Clinic (OBHC) was established in August 2020 as a joint clinical‐research service (O'Donoghue et al. 2023). In South Oxfordshire, National Health Service (NHS) patients with memory concerns are referred by their GPs to an Oxford Health NHS Foundation Trust memory clinic and may subsequently be referred to the OBHC for high‐quality multimodal assessments, including an MRI protocol aligned with UK Biobank (Griffanti et al. 2022). The results of these assessments are sent back to the referring memory clinic as a detailed report, which the psychiatrist takes into consideration during the follow‐up diagnostic appointment with the patient.

Here, we tested the suitability of employing UK Biobank image acquisition and analysis pipelines in patients referred to the OBHC. We evaluated the feasibility and acceptability of the protocol in this ‘real‐world’ population by assessing completion rates and data quality, including the use of automated quality control tools. Pipeline adaptations, previously described (Griffanti et al. 2022), were incorporated to overcome challenges associated with brain changes in older populations, and additional dementia‐informed measures were added to the standard set of IDPs. Finally, the utility of the standard and dementia‐informed IDPs was tested by performing a set of well‐established associations with age, cognition and diagnosis, including careful consideration of the most appropriate statistical adaptations (i.e., hierarchical false discovery rate procedure) for a clinical population.

## 2 Methods

### 2.1 OBHC Model and Patients

In the UK, NHS patients over 65 with memory concerns are typically referred by their GP to psychiatry‐based memory services. In Oxfordshire, these patients may be triaged by the memory clinic and referred to the OBHC if they are able to travel and eligible for an MRI scan. The OBHC appointment involves several high‐quality assessments as standard, including a detailed cognitive assessment and an MRI scan, and offers multiple avenues for optional research participation. Patients can consent to join the OBHC Research Database and can choose to complete a range of additional research assessments including: more time in the MRI scanner, a saliva sample for genotyping, and detailed questionnaires for both themselves and their relative. By reducing the barriers to research participation, this model makes the OBHC population highly representative of the ‘real‐world’ local memory clinic population. The OBHC Research Database was reviewed and approved by the South Central—Oxford C research ethics committee (SC/19/0404). Please refer to O'Donoghue et al. (2023) for more details about the protocols and assessments.

Assessment results are compiled into a report which is used by clinicians to support their diagnostic decision during the patient's subsequent memory clinic visit. As of May 2023, 213 patients were MRI scanned as part of their NHS assessment at the OBHC and consented to the use of their data for research purposes.

### 2.2 Joint Clinical‐Research Neuroimaging Protocol

The MRI scanning protocol used at the OBHC is available in Section 1 in Supporting Information. This protocol is aligned with the UK Biobank brain imaging protocol (Miller et al. 2016). It is restructured to prioritise the modalities with known clinical relevance (‘core clinical’ sequences: T1‐weighted, T2 fluid attenuated inversion recovery—T2‐FLAIR, and susceptibility‐weighted—swMRI) whilst enabling consenting patients to stay in the scanner for additional research sequences (diffusion MRI—dMRI, arterial spin labelling—ASL, and resting‐state functional MRI—rfMRI) (Griffanti et al. 2022).

The core clinical MRI scans for all patients are transferred to the NHS clinical records system and reported by a neuroradiologist (PP), using a structured radiology report template (Griffanti et al. 2022). The resulting report is sent to the referring memory clinic. If the patient gives consent for their data to be used for research, then the image files from all completed scans are pseudonymised and forwarded to a secure research server. Full details of the MRI protocol and operating procedures are available online (O'Donoghue et al. 2022).

### 2.3 Clinical Outcome Data

Cognitive performance is measured during the OBHC appointment with Addenbrooke's Cognitive Examination III (ACE‐III), which assesses cognition across five domains of memory, attention, language, fluency, and visuospatial skills. Patient diagnoses are based on the OBHC assessments, clinician judgement, and any additional assessments completed during the subsequent memory clinic visit, on average 2.5 months after their OBHC appointment. These diagnoses are extracted from secondary care electronic healthcare records (EHRs) (O'Donoghue et al. 2023). For this study, primary diagnoses were categorised as dementia (ICD10 codes F00, F01, F02, F03), mild cognitive impairment (MCI—F06.7), and no dementia‐related diagnoses (F10, F31, F32, F41, and patients who attended their memory clinic appointment but did not receive a formal diagnosis).

### 2.4 Pipeline Adaptations

**FIGURE 1:**
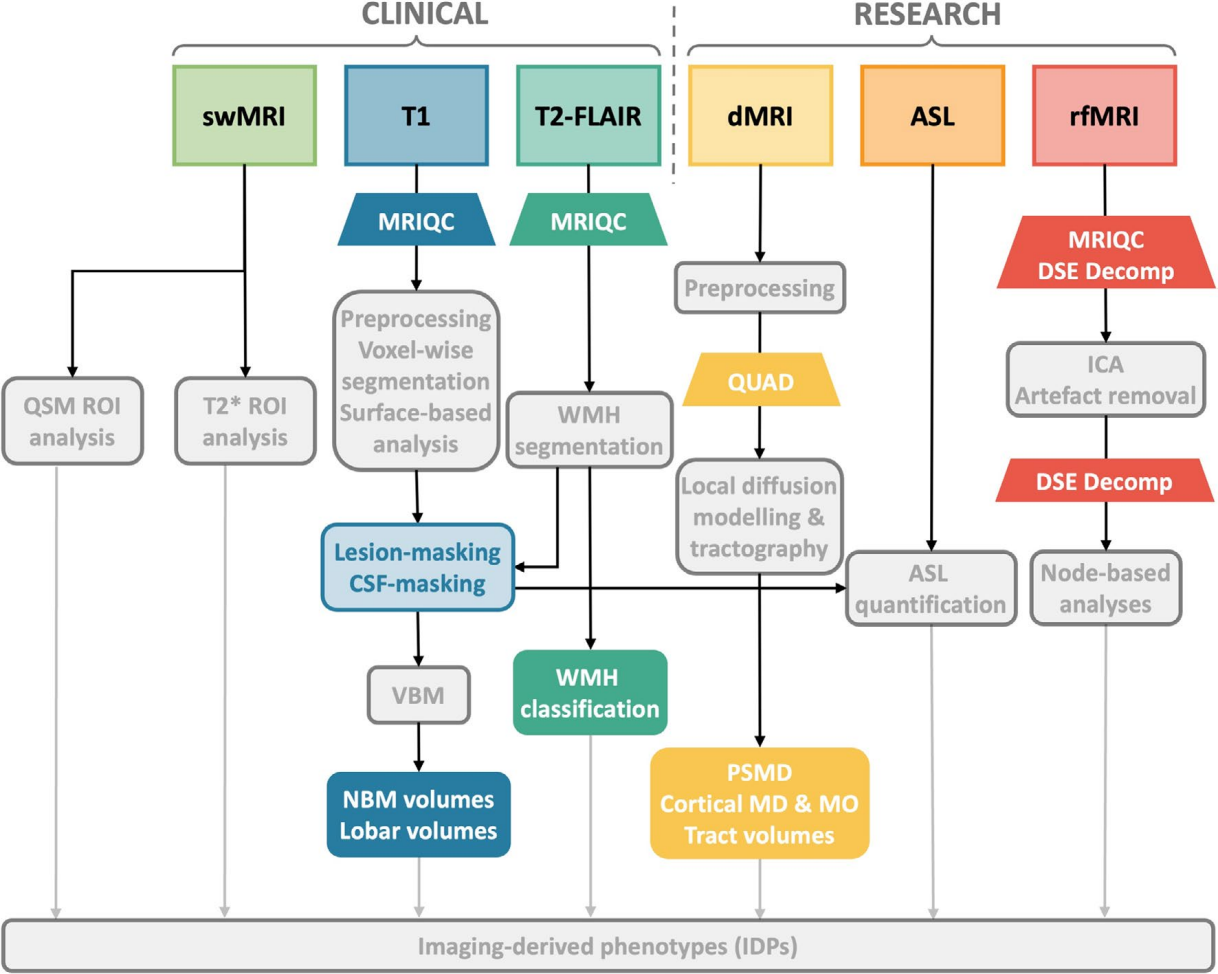
Simplified overview of the UK Biobank image analysis pipeline (in grey) with adaptations for OBHC use. Previously‐described adaptations (lesion‐masking and CSF‐masking) are in light blue, while novel adaptations and enhancements are in darker colours. See Alfaro‐Almagro et al. (2018) for details of the UKB pipeline. swMRI, susceptibility‐weighted MRI; T1, T1‐weighted imaging; T2‐FLAIR, T2‐weighted fluid attenuated inversion recovery imaging; dMRI, diffusion‐weighted MRI; ASL, arterial spin labelling; rfMRI, resting‐state functional MRI; QSM, quantitative susceptibility mapping; ROI, region of interest; MRIQC, MRI Quality Control tool (see (Esteban et al. 2017)); VBM, voxel‐based morphometry; NBM, nucleus basalis of Meynert; WMH, white matter hyperintensity; QUAD, QUality Assessment of DMRI (see (Bastiani et al. 2019)); PSMD, peak width of skeletonised mean diffusivity; MD, mean diffusivity; MO, mode of anisotropy; DSE, D‐var, S‐var, E‐var (see (Afyouni and Nichols 2018)); ICA, independent component analysis.

Scans were processed using a version of the UK Biobank image analysis pipeline (Alfaro‐Almagro et al. 2018; Smith, Alfaro‐Almagro, and Miller 2022). Figure 1 shows a schematic overview of the adapted pipeline. The pipeline was adapted to include lesion‐masking of the grey matter segmentations (SIENAX) and CSF‐masking of the hippocampal segmentations (FIRST), as previously described (Griffanti et al. 2022), to obtain accurate segmentations in the presence of atrophy and high vascular pathology. Downstream adaptations were also applied to the parts of the pipeline that rely on these corrected segmentations (ASL and VBM analyses). Additional IDPs were added to the pipeline to capture other putative dementia‐related changes, as described below.

#### 2.4.1 Lobar Volumes

Predominance of atrophy in one or more lobes can guide differential diagnosis, for example distinguishing between Alzheimer's disease, semantic dementia, and frontotemporal lobar degeneration (FTLD) (Rabinovici et al. 2007). Masks of the frontal, parietal, temporal, and occipital lobes (MNI structural atlas—see Collins et al. 1995; Mazziotta et al. 2001) were non‐linearly registered to the T1‐weighted scan and applied to the corrected SIENAX grey matter (GM) segmentation to yield lobar GM volumes.

#### 2.4.2 WMH Volumes

The default UK Biobank pipeline calculates the periventricular, deep, and total white matter hyperintensity (WMH) volumes derived using BIANCA (Griffanti et al. 2016). Given the relevance of the spatial distributions of WMHs to underlying dementia‐related pathologies (Biesbroek et al. 2016, 2024; Veldsman et al. 2020), we additionally generated tract‐wise WMH volumes by calculating the overlap of the WMH mask with 48 white matter (WM) tract masks registered to T1 space (Mori et al. 2008).

#### 2.4.3 Cholinergic Analyses

Given the relevance of the cholinergic systems in dementia (Bohnen et al. 2018), additional IDPs were added to measure the volumes of the left and right nucleus basalis of Meynert (NBM) in the basal forebrain. A widely used histologically‐defined NBM mask (Zaborszky et al. 2008), non‐linearly registered to T1 space, was masked with the GM partial volume estimate.

#### 2.4.4 Additional dMRI Metrics

The peak width of the skeletonised mean diffusivity (PSMD) is a robust marker of overall cerebrovascular pathology and holds biomarker potential in dementia (Low et al. 2020; Satizabal et al. 2020). It shows good correlations with cognition, explaining variability in cognition beyond traditional measures of cerebrovascular‐related WM changes such as WMH volume (Satizabal et al. 2020). PSMD was calculated as the width between the 5th and 95th percentiles of the skeletonised mean diffusivity maps, aligned with the protocol outlined by Baykara et al. (2016).

Tractography‐defined WM tract volumes have also been proposed as estimates of tract‐specific atrophy with moderate subject‐differentiating power (Besseling et al. 2012; de Groot et al. 2015). Following Warrington et al. (2020), we binarised each tractography‐defined tract, normalised for the number of valid streamlines and registered to dMRI space, at 0.005 and calculated its volume.

Grey matter mean diffusivity (MD) metrics are also gaining popularity as potential biomarkers in dementia and cognitive decline (Douaud et al. 2011, 2022; Illán‐Gala et al. 2019; Montal et al. 2021; Weston et al. 2020). The mode of anisotropy (MO) has demonstrated sensitivity to WM changes in MCI and early dementia (Douaud et al. 2011), although it seems potentially less informative in GM structures (Beer, Plank, and Greenlee 2020; Douaud et al. 2013). Using the GM partial volume estimate (PVE) after lesion‐masking, weighted MD and MO were calculated within the corrected FIRST segmentations of the hippocampi and amygdala as well as five ROIs defined with the UKB GM atlas: parahippocampal gyrus, precuneus, superior frontal cortex, superior parietal cortex and supramarginal cortex.

### 2.5 Integrated Quality Control

Automated QC of the scans was integrated into the analysis pipeline where possible. Details of the image‐quality metrics (IQMs) included in the different tools are summarised in Table 1. MRIQC (Esteban et al. 2017) was run for T1 and T2‐FLAIR, EDDY QUAD (Bastiani et al. 2019) for dMRI, and both MRIQC and DSE decomposition (Afyouni and Nichols 2018) for rfMRI. Given the importance of T1 as an anatomical reference for other modalities and all analyses, we do perform manual QC using static visual summaries (nine orthogonal slices, generated with the FSL command slicesdir) of T1 scans, brain extracted and registered to MNI space. FIRST outputs are overlaid on the CSF map (output of FAST), and structures with full overlap are identified as mislocalised subcortical segmentations and excluded from subsequent analyses.

**TABLE 1:** Automated quality control (QC) tools with categories of image‐quality metrics (IQMs).

Tool	Image‐quality metric (IQM) categories
MRIQC: T1 and T2‐FLAIR	Noise measures (*CJV for T1, CNR, SNR within CSF, GM, and for T1 WM, QI2*); information theory measures (*EFC, FBER*); bias field measures (*INU_range for T1, INU_med*); carotid vessels/fat hyperintensity (*WM2MAX*)
MRIQC: rfMRI	Noise measures (*SNR, tSNR, GSR*); information theory measures (*EFC, FBER*); global correlation (*GCOR*); outliers (*AOR*); quality (*AQI*)
DSE decomposition: rfMRI	Global and whole % S‐var and % D‐var (pre‐ and post‐FIX)
EDDY QUAD: dMRI	Head motion; susceptibility; outliers; noise measures

*Note:* For details about the individual measures please refer to Esteban et al. (2017) for MRIQC, Afyouni and Nichols (2018) for DSE, and Bastiani et al. (2019) for EDDY QUAD.

Scans with outliers (> 1.5 IQR away from Q1/Q3) in at least one of the IQMs or visual inspection of the T1 summary were flagged for further inspection of the core pipeline outputs: corrected SIENAX and FIRST segmentations for T1, BIANCA segmentations for T2‐FLAIR, tractography from dMRI, and preprocessed DSE decomposition for rfMRI. Low‐quality raw or derived images were excluded from subsequent analysis.

### 2.6 Statistics

Numeric variables, including all IDPs, ACE‐III scores, and age, were normalised to unit variance using quantile normalisation (Peterson and Cavanaugh 2020), implemented with the R package bestNormalise (Peterson 2021). Due to sample size limiting the degrees of freedom compared to UKB, we compromised on the number of covariates and included only age, sex, and head size. We investigated the extent to which IDPs predict age and cognition by performing linear regression with each IDP and covariates (Equations 1 and 2). We included total cognitive score as a covariate in the linear regression with age (Equation 1) to highlight age‐related changes that are not purely related to cognitive impairment in this memory clinic population where cognition may be expected to account for a large share of the observed variance. Associations with diagnoses were assessed with ordinal regression (Equation 3). We additionally investigated associations of clinical phenotypes with T2*, QSM, and tractography IDPs when controlling for ROI/tract volumes.

(1) $$\mathrm{Age}=\beta_{1}\mathrm{IDP}+\beta_{2}\mathrm{Sex}+\beta_{3}\text{Headsize}+\beta_{4}\mathrm{ACE}$$

(2) $$\mathrm{ACE}=\beta_{1}\mathrm{IDP}+\beta_{2}\mathrm{Age}+\beta_{3}\mathrm{Sex}+\beta_{4}\text{Headsize}$$

(3) $$\text{Diagnosis}=\beta_{1}\mathrm{IDP}+\beta_{2}\mathrm{Age}+\beta_{3}\mathrm{Sex}+\beta_{4}\text{Headsize}$$

In order to control for multiple testing across modalities while enabling interpretation within modality, we used a hierarchical false discovery rate procedure (Yekutieli 2008). This guarantees that the false discovery rate (FDR, the expected proportion of false positives among detections) is controlled within each modality. The method proceeds by computing omnibus *p*‐values for each modality with a Simes test, and then the modalities that are significant at the 5% FDR level have their IDPs tested with FDR that uses a more stringent threshold according to the number of significant omnibus *p*‐values.

## 3 Results

### 3.1 Demographics

**FIGURE 2:**
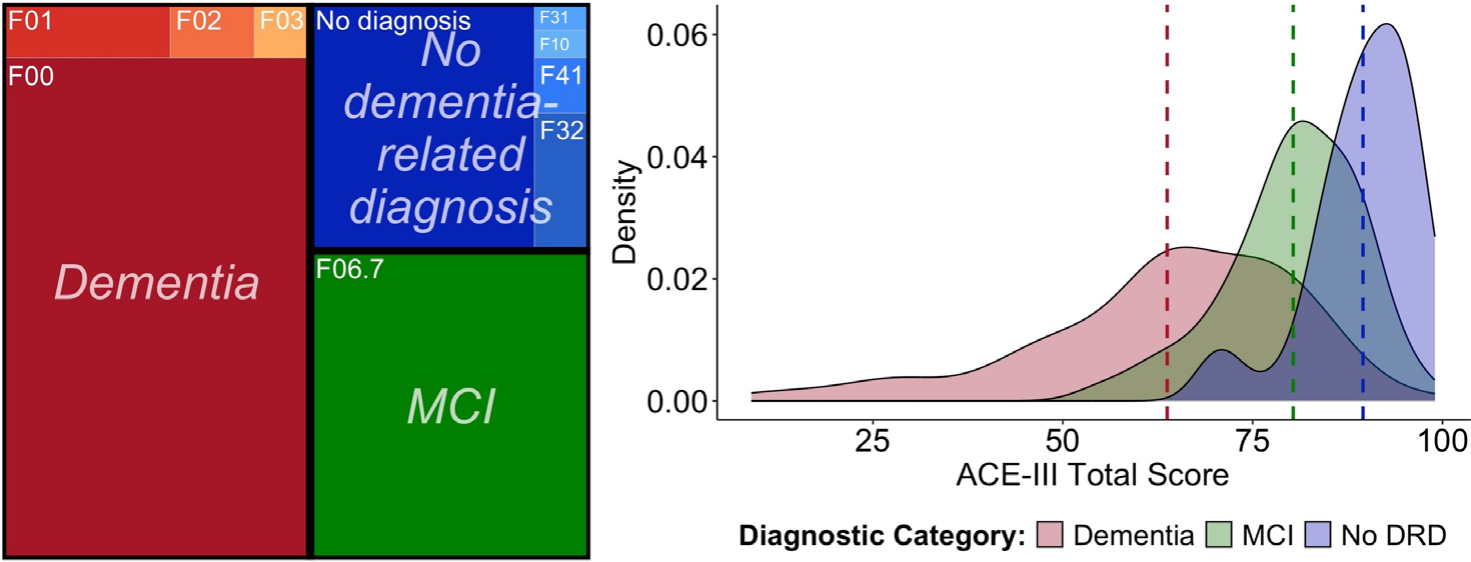
Subsequent diagnoses of OBHC patients and distribution of cognitive scores across the diagnostic groups. Dashed lines on density plot are the group means. ACE‐III, Addenbrooke's Cognitive Examination III; DRD, dementia‐related diagnosis; F00, Alzheimer's dementia; F01, vascular dementia; F02, dementia in other diseases classified elsewhere; F03, unspecified dementia; F06.7, mild cognitive impairment; F10, alcohol‐related disorders; F31, bipolar disorder; F32, depressive episode; F41, other anxiety disorders.

See Table 2 for OBHC patient demographics. Figure 2 illustrates the prevalence of diagnoses that were obtained subsequently, grouped into three diagnostic categories (dementia, MCI, and no dementia‐related diagnosis) along with the distributions of ACE‐III cognitive scores for these diagnostic groups.

**TABLE 2:** Demographics of MRI‐scanned OBHC patient population: Overall and stratified by the subsequent diagnostic category.

Characteristic	Overall	Stratified by diagnostic category		
Sample size	*N* = 213	Dementia (*N* = 111)	MCI (*N* = 56)	No DRD (*N* = 45)
Age (years)—mean ± SD (range)	78.0 ± 6.2 (65–101)	79.8 ± 6.5 (65–101)	76.8 ± 5.5 (66–88)	74.9 ± 4.7 (65–85)
ACE‐III total score—mean ± SD (range)	73.9 ± 17.4 (9–99)	63.5 ± 17.3 (9–98)	80.3 ± 8.8 (55–97)	89.5 ± 6.9 (70–99)
Years FT education—mean ± SD (range) [*N*]	13.1 ± 3.5 (3–21) [*N* = 192]	12.9 ± 3.6 (3–21) [*N* = 99]	12.7 ± 3.5 (4–21) [*N* = 52]	14.1 ± 3.3 (9–21) [*N* = 40]
Clinical frailty score—mean ± SD (range)	2.9 ± 1.4 (1–7)	3.2 ± 1.5 (1–7)	2.7 ± 1.2 (1–6)	2.4 ± 1.1 (1–6)
ApoE—% with at least 1 E4 allele [*N*/total genotyped]	41.4% [55/133]	49.2% [31/63]	37.5% [12/32]	31.6% [12/38]

*Note:* One patient missing a diagnosis was excluded from stratified results.

Abbreviations: ACE‐III, Addenbrooke's Cognitive Examination III; ApoE, Apolipoprotein E; DRD, dementia‐related diagnosis.

### 3.2 Feasibility and Acceptability

As of May 2023, 244 patients attended the OBHC, 93.4% (*N* = 228) of whom consented to join the OBHC Research Database. 93.4% of these patients (*N* = 213) were able to complete at least one of the UKB‐aligned MRI sequences (excluded patients had MRI contraindications [*N* = 4], incompatible body habitus/kyphosis [*N* = 4], claustrophobia [*N* = 3], or discomfort/anxiety [*N* = 4]). 211 patients completed all core clinical sequences (swMRI, T1, T2‐FLAIR; 16 min and 29 s), with two scans terminated prematurely due to patient discomfort. Of the 165 patients (67.6%) that initially consented to the additional MRI research sequences, 127 were still able and willing to stay in the scanner when asked after the clinical sequences. 120 patients (56.3% of those who started the scanning protocol) completed all sequences (37 min and 46 s total).

**FIGURE 3:**
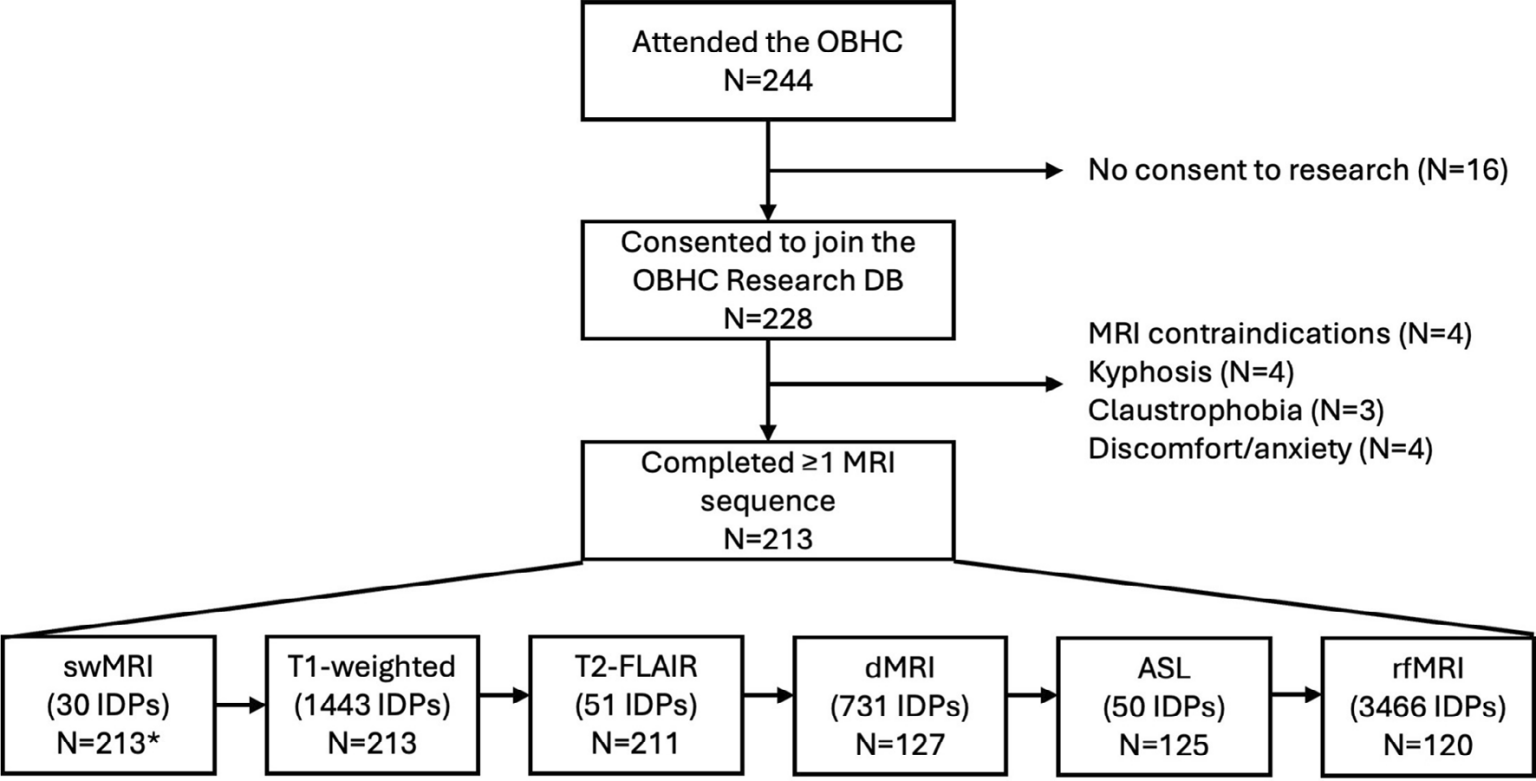
Available MRI data with the number of IDPs generated from each modality. One scan was unsuccessfully archived from the scanner and excluded from subsequent analyses. *For 19 swMRI scans with incomplete data (uncombined channel images unavailable), the scanner‐reconstructed swMRI images were used as inputs for the core swMRI pipeline, but the QSM pipeline was skipped since it relies on phase images prior to high‐pass filtering. See Section 2 in Supporting Information for the equivalence of the T2* IDPs generated from the uncombined and scanner‐combined images.

Figure 3 summarises the available MRI data. The high rates of willing consent and completion indicate that the protocol is feasible and acceptable. Integrating this research‐quality scanning protocol into clinical workflows via the OBHC also yielded high rates of MRI scans suitable for detailed radiology reporting for clinical purposes. From the 213 patients that started the MRI session, the neuroradiologist completed 212 full structured radiology reports and 1 partial report due to scan termination. One additional scan was noted as poor quality, but this did not impede reporting.

### 3.3 Quality Control

Enhanced QC of the T1‐weighted, T2‐FLAIR, dMRI, and rfMRI scans flagged 14%–24% of scans for further inspection (Table 3). See Section 3 in Supporting Information for counts of IQMs flagged for each modality. Visual inspection of core outputs from T1, T2‐FLAIR, and dMRI revealed that 0%–2.4% of segmentations were low quality and subsequently excluded. Visual inspection of DSE plots revealed that although residual noise was high (S‐var > 75% at timepoints) in 97.1% (33/34) of processed rfMRI data, no scans were deemed unusable based on this criterion. See Section 4 in Supporting Information for full visual QC results.

**TABLE 3:** Quality control results.

Modality	% Flagged (*N*/total)	% Excluded (*N*/total)
T1	14.6% (31/212)	1.4% (3/212) all IDPs 2.4% (5/212) one or more IDPs
T2‐FLAIR	16.7% (35/210)	1.0% (2/210)
dMRI	19.0% (24/126)	0% (0/126)
rfMRI	Pre‐FIX: 24.2% (29/120) Post‐FIX: 10.0% (12/120)	0% (0/120)

### 3.4 Associations With Age

**FIGURE 4:**
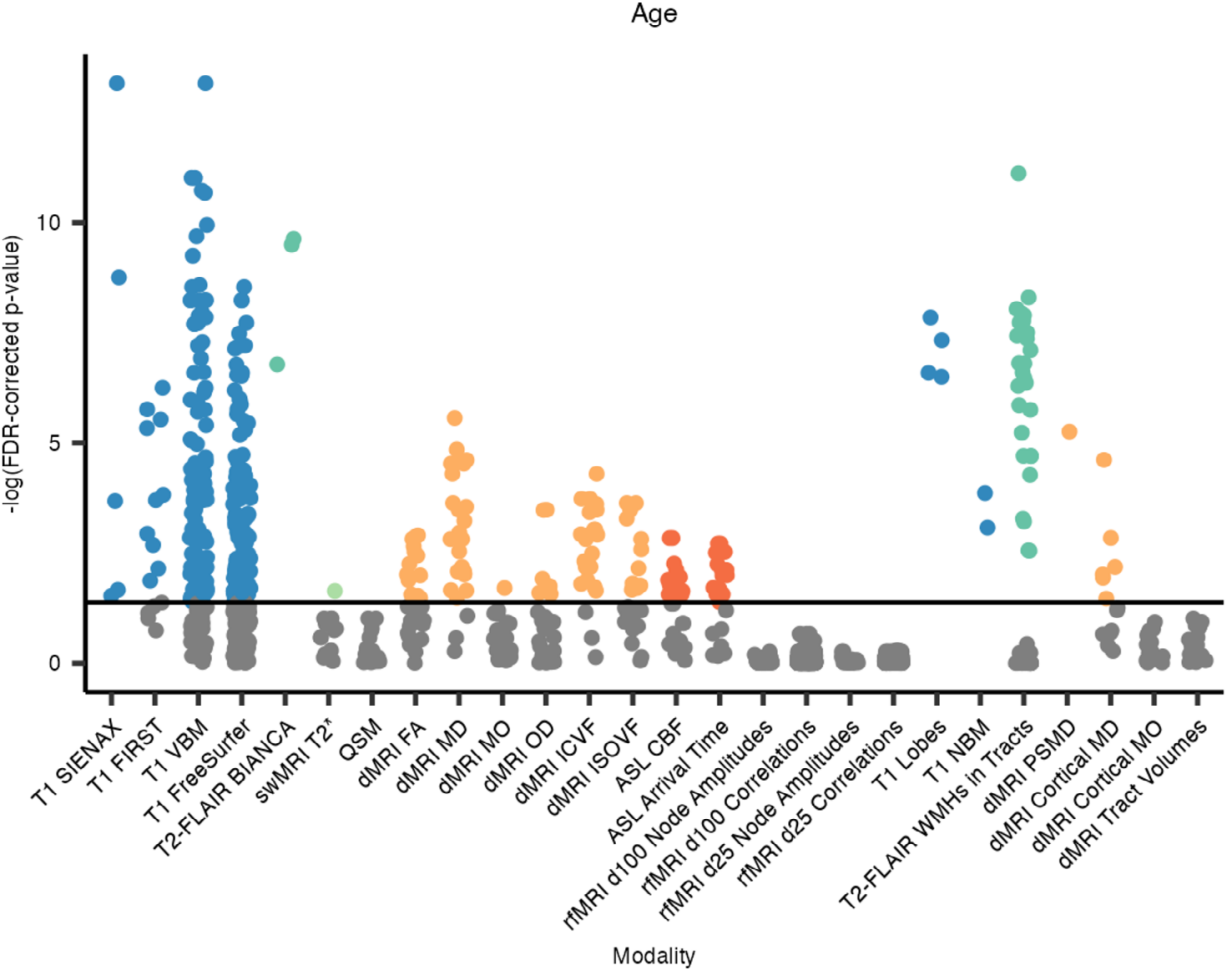
FDR‐corrected *p*‐values, hierarchical by modality, for associations between IDPs and age. Each dot represents one IDP, grouped by analysis tool/method and colour‐coded by scan modality if significant. BIANCA, Brain Intensity AbNormality Classification Algorithm; CBF, cerebral blood flow; FA, fractional anisotropy; FIRST, FMRIB's Integrated Registration and Segmentation Tool; ICVF, intra‐cellular volume fraction; ISOVF, isotropic volume fraction; MD, mean diffusivity; MO, mode of anisotropy; NBM, nucleus basalis of Meynert; OD, orientation dispersion index; PSMD, peak width of skeletonised mean diffusivity; SIENAX, Structural Image Evaluation using Normalisation of Atrophy (cross‐sectionally); VBM, voxel‐based morphometry; WMH, white matter hyperintensity.

**FIGURE 5:**
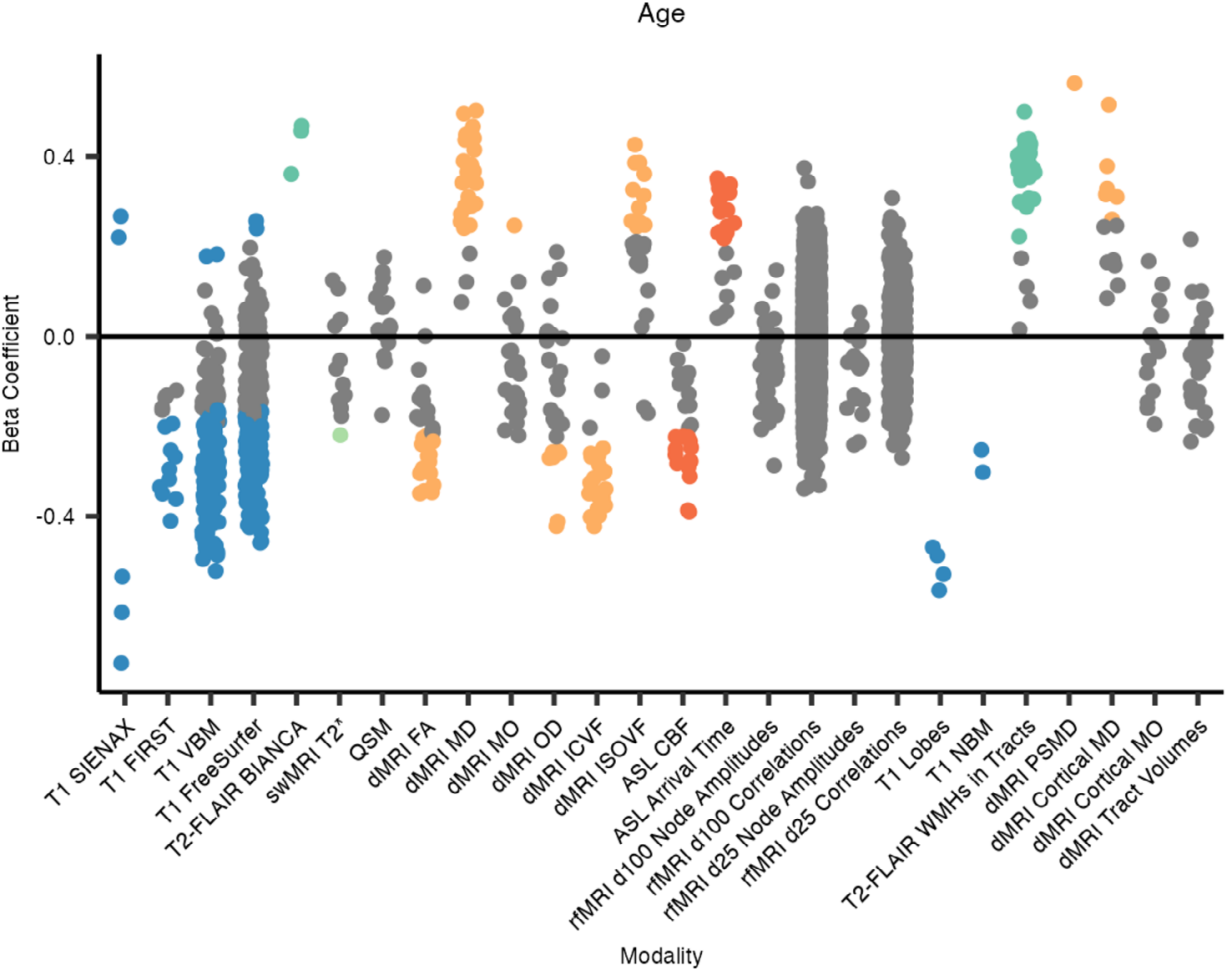
Beta coefficients for associations between IDPs and age. All numeric variables are unit standardised, meaning that 1 standard deviation (SD) increase in an IDP value is associated with a *β* SD difference in age. Coloured dots indicate associations significant at the 5% FDR level, hierarchical by modality. BIANCA, Brain Intensity AbNormality Classification Algorithm; CBF, cerebral blood flow; FA, fractional anisotropy; FIRST, FMRIB's Integrated Registration and Segmentation Tool; ICVF, intra‐cellular volume fraction; ISOVF, isotropic volume fraction; MD, mean diffusivity; MO, mode of anisotropy; NBM, nucleus basalis of Meynert; OD, orientation dispersion index; PSMD, peak width of skeletonised mean diffusivity; SIENAX, Structural Image Evaluation using Normalisation of Atrophy (cross‐sectionally); VBM, voxel‐based morphometry; WMH, white matter hyperintensity.

Using covariates of sex, head size, and total cognitive score (ACE‐III; see Equation 1), IDPs from 5 out of the 6 MRI modalities were significantly associated with age (Figures 4 and 5). The strongest negative associations were observed with volumetric measures derived from the T1‐weighted scans (240 significant associations), with the strongest associations being with the total grey matter volume (SIENAX) and some cerebellar volumes (VBM). Periventricular, deep, and total white matter hyperintensity (WMH) volumes all positively associated with age. Mean T2* in the right amygdala was negatively associated with age, while no other swMRI‐derived metrics survived correction for multiple testing. The fractional anisotropy (FA) of 14 tracts negatively associated with age, while mean diffusivity (MD) from 24 tracts positively associated with age. The NODDI‐derived metrics ICVF and ISOVF negatively and positively associated with age, respectively, in many tracts. Thirty‐two ASL‐derived measures associated with age (negative associations with cerebral blood flow and positive associations with arrival time). No rfMRI‐derived IDPs were significantly associated with age. A similar pattern was found across all IDPs when not covarying for total cognitive score (Section 5 in Supporting Information).

Regarding the additional dementia‐informed IDPs, all four lobar grey matter (GM) volumes negatively associated with age, with the strongest being with the temporal GM volume. Volumes of the bilateral nuclei basalis of Meynert also negatively associated with age. WMH volumes within 30 tracts were positively associated with age, with the most significant being the WMH volume in the right superior corona radiata. The peak width of the skeletonised MD (PSMD) positively associated with age, as did six cortical MD IDPs. See Section 6 in Supporting Information for the full list of associations that were significant following hierarchical FDR correction for multiple testing.

When controlling for ROI/tract volumes, no swMRI‐derived IDPs and fewer tractography‐based IDPs survived hierarchical FDR correction (Section 7 in Supporting Information).

### 3.5 Associations With Cognition

**FIGURE 6:**
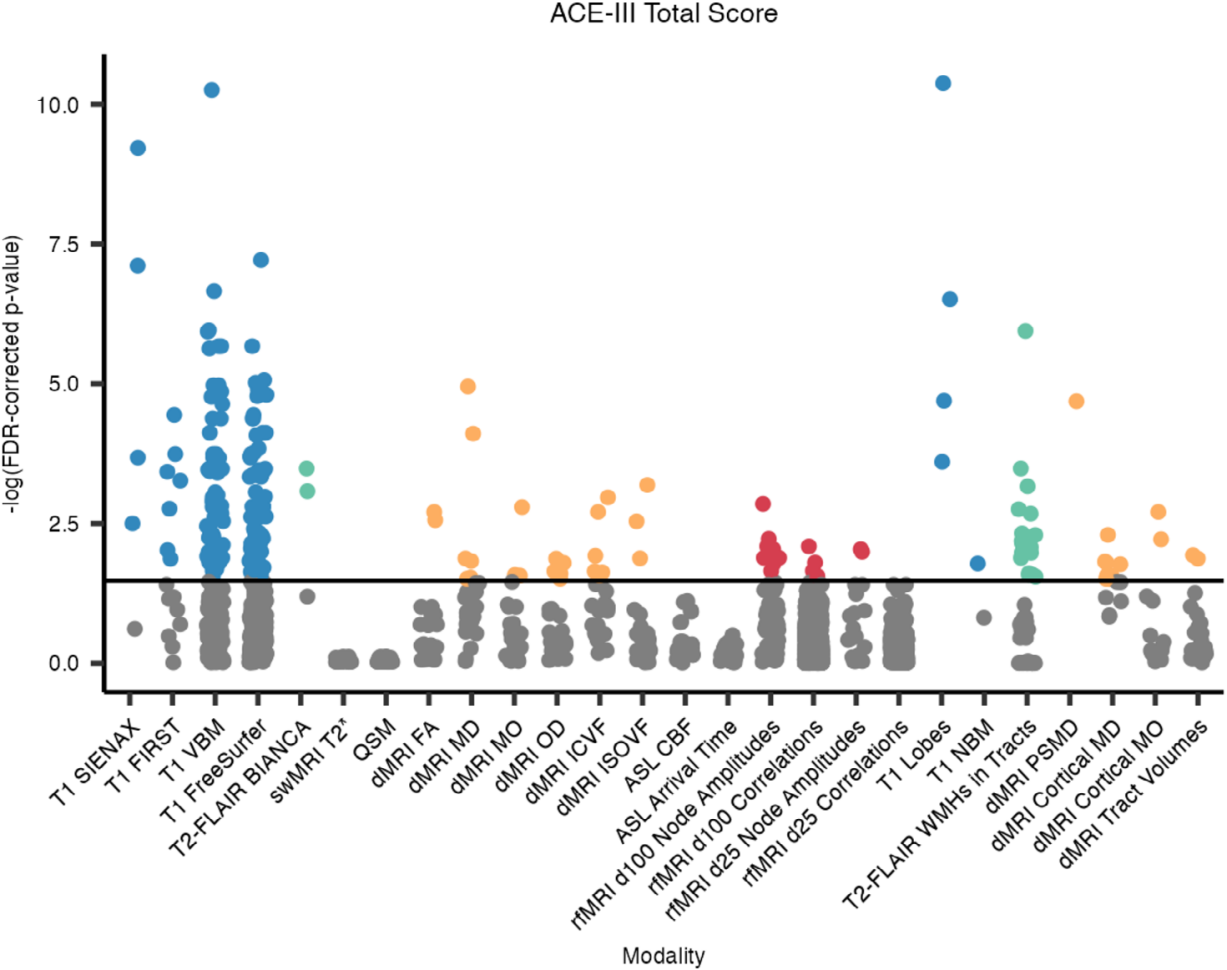
FDR‐corrected *p*‐values, hierarchical by modality, for associations between IDPs and ACE‐III total cognitive scores. Each dot represents one IDP, grouped by analysis tool/method and colour‐coded by scan modality if significant. BIANCA, Brain Intensity AbNormality Classification Algorithm; CBF, cerebral blood flow; FA, fractional anisotropy; FIRST, FMRIB's Integrated Registration and Segmentation Tool; ICVF, intra‐cellular volume fraction; ISOVF, isotropic volume fraction; MD, mean diffusivity; MO, mode of anisotropy; NBM, nucleus basalis of Meynert; OD, orientation dispersion index; PSMD, peak width of skeletonised mean diffusivity; SIENAX, Structural Image Evaluation using Normalisation of Atrophy (cross‐sectionally); VBM, voxel‐based morphometry; WMH, white matter hyperintensity.

**FIGURE 7:**
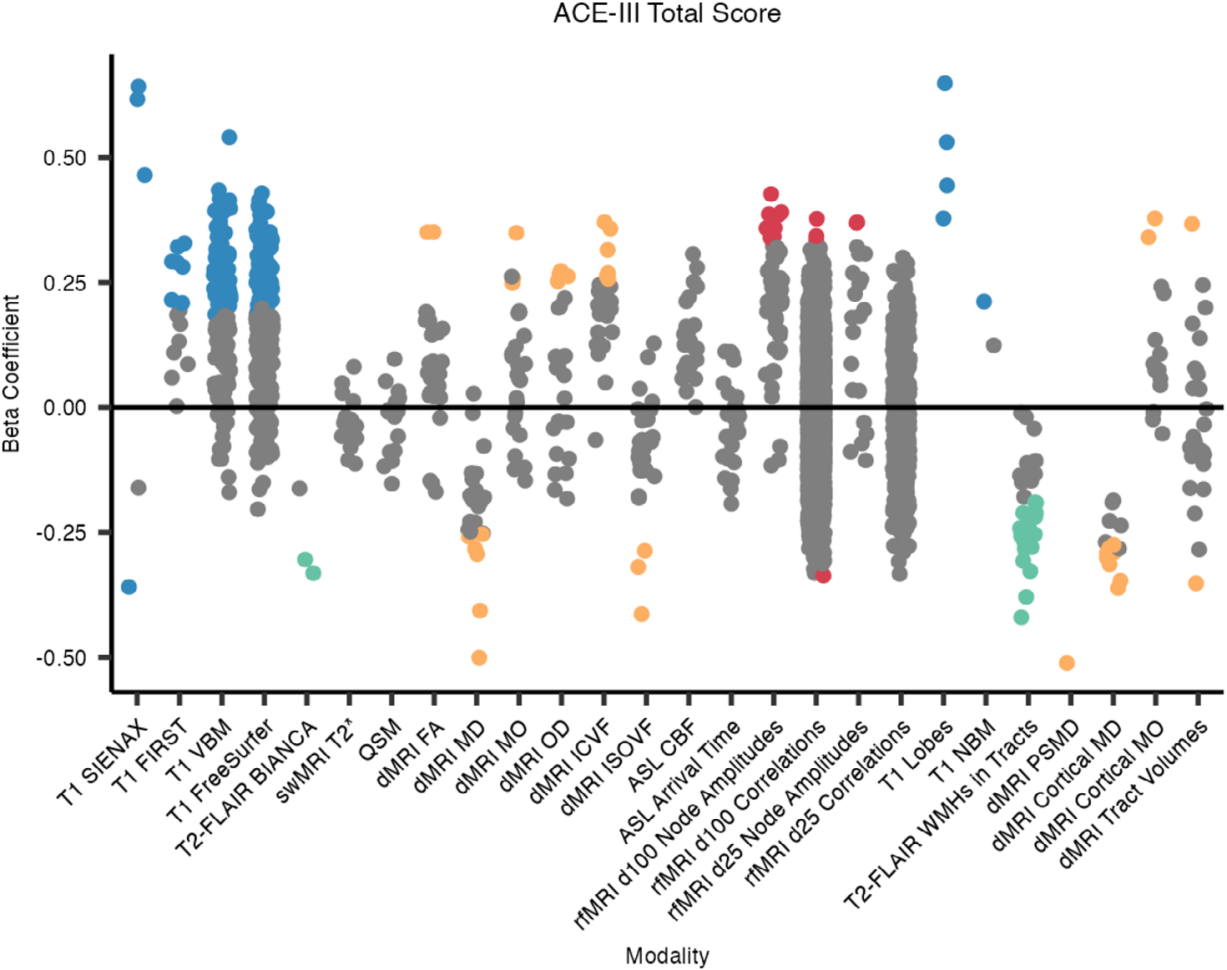
Beta coefficients for associations between IDPs and ACE‐III total cognitive scores. All numeric variables are unit standardised, meaning that 1 standard deviation (SD) increase in an IDP value is associated with a *β* SD difference in ACE‐III total cognitive score. Coloured dots indicate associations significant at the 5% FDR level, hierarchical by modality. BIANCA, Brain Intensity AbNormality Classification Algorithm; CBF, cerebral blood flow; FA, fractional anisotropy; FIRST, FMRIB's Integrated Registration and Segmentation Tool; ICVF, intra‐cellular volume fraction; ISOVF, isotropic volume fraction; MD, mean diffusivity; MO, mode of anisotropy; NBM, nucleus basalis of Meynert; OD, orientation dispersion index; PSMD, peak width of skeletonised mean diffusivity; SIENAX, Structural Image Evaluation using Normalisation of Atrophy (cross‐sectionally); VBM, voxel‐based morphometry; WMH, white matter hyperintensity.

IDPs from 4 out of the 6 MRI modalities were significantly associated with ACE‐III total scores, after controlling for age, sex, and head size (Figures 6 and 7). Regarding the core UK Biobank‐aligned IDPs, most of the significant associations were with volumetric measures derived from the T1‐weighted scans (160 significant associations), with the strongest positive associations being with volumes of the posterior left middle temporal gyrus (VBM) and the peripheral GM (SIENAX). Periventricular and total white matter hyperintensity (WMH) volumes negatively associated with ACE‐III total scores. Of the core dMRI‐derived IDPs, most significant ones relate to the parahippocampal cinguli. Mean diffusivity (MD) metrics in the parahippocampal cinguli were most strongly negatively associated with cognition, while most other significant associations, such as with FA, MO, and ICVF, are positive. Eleven node amplitude IDPs from rfMRI were positively associated with cognition, primarily relating to the salience, frontoparietal, and language networks.

Regarding the additional dementia‐informed IDPs, all four lobar grey matter (GM) volumes positively associated with ACE‐III, with the strongest being with the temporal GM volume. WMH volumes within 20 tracts were negatively associated with ACE‐III, with the most significant being the WMH volume in the splenium of the corpus collosum. PSMD negatively associated with cognition and was the second‐most significant dMRI‐derived metric overall. Cortical MD metrics, including in the bilateral supramarginal gyri, hippocampi, amygdalae, and parahippocampal cinguli, were negatively associated with cognitive scores, while MO in the bilateral supramarginal gyri positively associated with cognition. See Section 8 in Supporting Information for the full list of significant associations.

When controlling for ROI/tract volumes, no swMRI‐derived IDPs and fewer tractography‐based IDPs survived hierarchical FDR correction (Section 9 in Supporting Information).

### 3.6 Associations With Diagnoses

**FIGURE 8:**
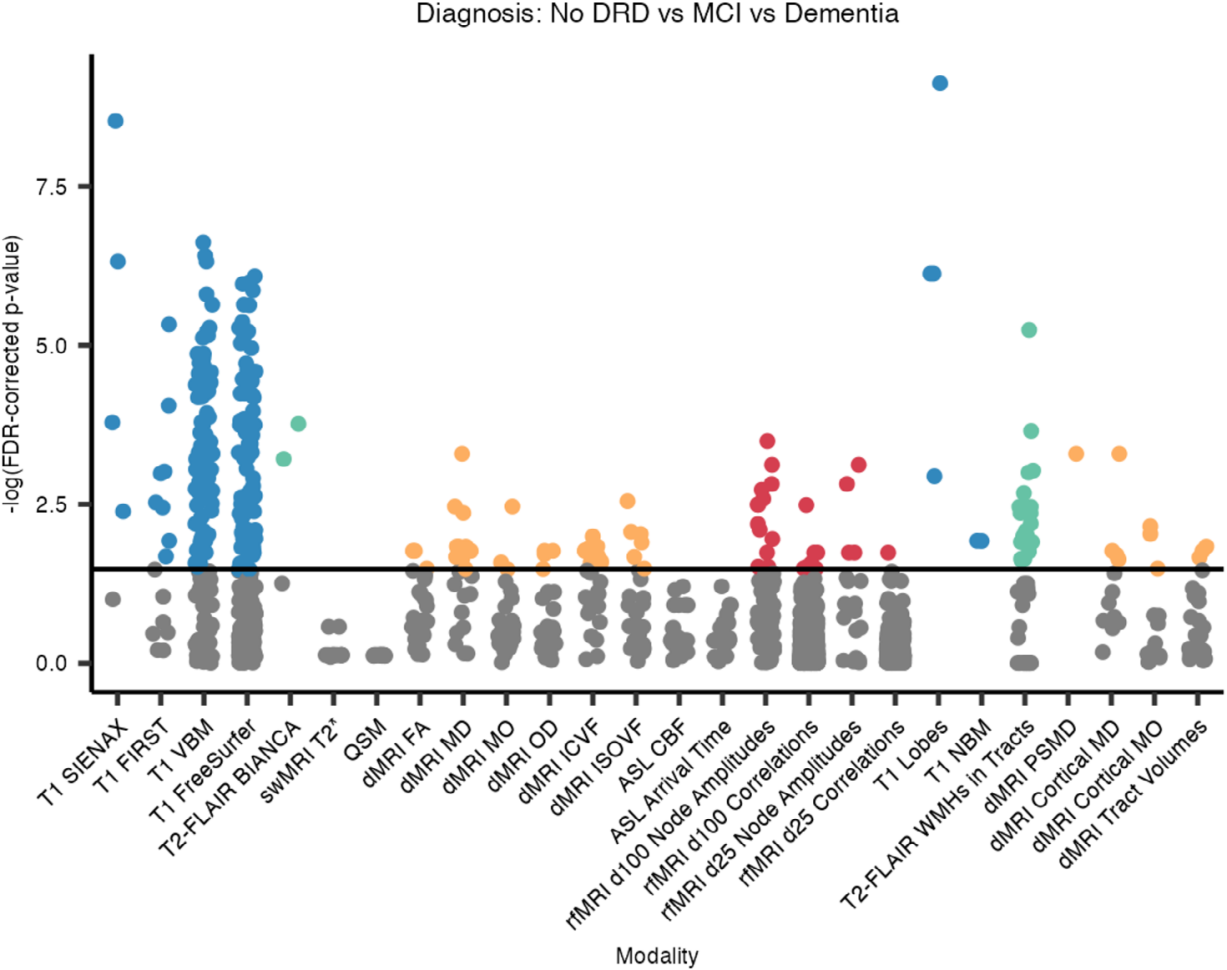
FDR‐corrected *p*‐values, hierarchical by modality, for associations between IDPs and diagnostic groups. Each dot represents one IDP, grouped by analysis tool/method and colour‐coded by scan modality if significant. BIANCA, Brain Intensity AbNormality Classification Algorithm; CBF, cerebral blood flow; DRD, dementia‐related diagnosis; FA, fractional anisotropy; FIRST, FMRIB's Integrated Registration and Segmentation Tool; ICVF, intra‐cellular volume fraction; ISOVF, isotropic volume fraction; MD, mean diffusivity; MO, mode of anisotropy; NBM, nucleus basalis of Meynert; OD, orientation dispersion index; PSMD, peak width of skeletonised mean diffusivity; SIENAX, Structural Image Evaluation using Normalisation of Atrophy (cross‐sectionally); VBM, voxel‐based morphometry; WMH, white matter hyperintensity.

**FIGURE 9:**
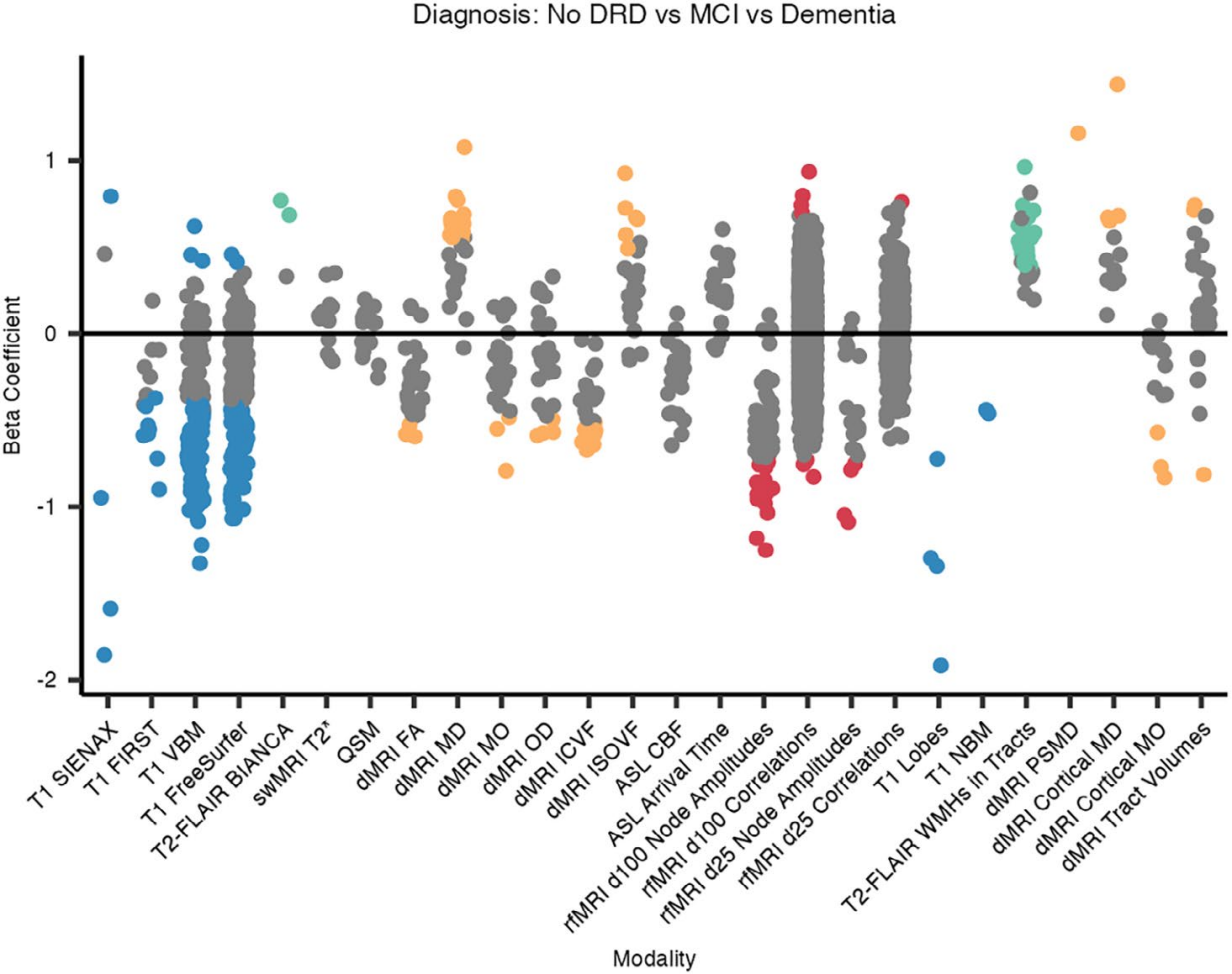
Beta coefficients for associations between IDPs and diagnostic groups. All numeric variables are unit standardised, meaning that for a 1 standard deviation (SD) increase in an IDP value, the log‐odds of receiving a dementia‐related diagnosis increases (or decreases if negative) by the *β* coefficient. Coloured dots indicate associations significant at the 5% FDR level, hierarchical by modality. BIANCA, Brain Intensity AbNormality Classification Algorithm; CBF, cerebral blood flow; DRD, dementia‐related diagnosis; FA, fractional anisotropy; FIRST, FMRIB's Integrated Registration and Segmentation Tool; ICVF, intra‐cellular volume fraction; ISOVF, isotropic volume fraction; MD, mean diffusivity; MO, mode of anisotropy; NBM, nucleus basalis of Meynert; OD, orientation dispersion index; PSMD, peak width of skeletonised mean diffusivity; SIENAX, Structural Image Evaluation using Normalisation of Atrophy (cross‐sectionally); VBM, voxel‐based morphometry; WMH, white matter hyperintensity.

A similar pattern of associations is observed between IDPs and diagnostic groups (dementia, MCI, no dementia‐related diagnosis; Figures 8 and 9). This is likely because the results of cognitive assessment play an important role in dementia diagnosis. See Section 10 in Supporting Information for the full list of significant associations.

## 4 Discussion

In this study, we have optimised a well‐established imaging framework for a typical NHS memory clinic population by integrating enhanced quality control processes and dementia‐specific analyses. Our detailed QC revealed that the imaging protocol is feasible, acceptable, and yields sufficiently high‐quality data that is usable for both clinical and research purposes. We found that this enhanced pipeline can generate reliable UKB‐aligned IDPs and dementia‐informed supplementary IDPs. We replicated established associations from earlier work and found novel associations between brain metrics and age, cognition, and diagnosed dementia. Together, these findings validate the use of this enhanced methodology in this real‐world population and demonstrate its potential to bridge the gap between big data studies and clinical settings.

The OBHC model of integrated, tiered research opportunities (O'Donoghue et al. 2023) continues to yield high rates of consent for research participation (93.4%) and additional scanner time (67.6%). Completion rates likewise remain high, with 99.1% and 72.7% of consenting patients completing the clinical and research sequences, respectively. All but one of the 213 scans were usable for radiologist reporting and therefore provided usable, structured data to the memory clinic.

One strength of this study is the seamless integration of rigorous QC measures into the UKB pipeline, creating a comprehensive framework for analysing real‐world datasets. Automated QC tools (MRIQC, QUAD, and DSE decomposition) were used to flag T1‐weighted, T2‐FLAIR, dMRI, and rfMRI scans for visual inspection. We performed detailed QC at the level of individual IQMs, inspecting any scans that were outliers for at least one of these metrics. This process revealed that most of the raw data and pipeline outputs were useable. Although we accept that our outlier flagging approach might be overly sensitive, the percentage of flagged and excluded scans or IDPs in our study (Table 3) is consistent with other studies including automated QC and visual inspection. For example, in a sample of 4282 scans by Alfaro‐Almagro et al. (2018), 17.5% of T1‐weighted scans were flagged for inspection and 1.7% were excluded. Hence, this work supports the clinical application of automated QC tools as part of a staged QC approach. In clinical settings, we cannot afford to blindly exclude data based solely on fixed criteria or metrics, and we have demonstrated that this approach is useful to reduce the burden of visual inspection.

The analysis pipeline generates IDPs that are aligned with UK Biobank, while being suitable and relevant to a memory clinic population. In addition to incorporating previously described modifications to account for the high degree of atrophy and vascular burden (Griffanti et al. 2022), we added additional IDPs to capture other previously reported dementia‐related changes. Many of these additional analyses are computationally light, ensuring that they will not significantly increase the computational load beyond the existing UKB pipeline. The code is openly available on Gitlab (https://git.fmrib.ox.ac.uk/open‐science/analysis/brain‐health‐clinic‐mri).

We were then able to validate our obtained IDPs by replicating associations with well‐known brain health changes in this real‐world memory clinic population, carefully controlling multiple testing within each modality with a hierarchical FDR procedure. As anticipated, associations with age, cognition, and diagnoses were mainly with volumetric IDPs, particularly corresponding to temporal lobe structures. Associations with periventricular and total WMH volumes were stronger than with deep WMH volumes, in keeping with research studies in older adults (Bolandzadeh et al. 2012; Griffanti et al. 2018). Some of the strongest dMRI‐derived associations were with the bilateral parahippocampal cinguli, which have previously been widely implicated in dementia and cognitive decline (Bozzali et al. 2011; Hirschfeld et al. 2023). In addition to the tensor‐based metrics (e.g., MD), NODDI‐derived metrics (i.e., ICVF and ISOVF) also show significant associations with clinical phenotypes, consistent with the global findings of McCracken et al. (2022). From rfMRI, associations with node amplitudes were most consistent. Using the same node labels as Lee et al. (2023), left fronto‐parietal and language node amplitudes were positively associated with cognition meanwhile higher node amplitudes in the default mode, salience, and attention networks were associated with lower odds of receiving a dementia‐related diagnosis.

The dementia‐informed IDPs may capture additional variability in this memory clinic population. Lobar grey matter volumes have well‐established relevance to cognitive status and prognosis, with the smaller temporal lobe volumes particularly associated with cognitive decline and dementia (Harper et al. 2017; Rabinovici et al. 2007; Visser et al. 2002; Woodworth et al. 2022). We, too, found the strongest associations with the temporal lobe (age: *β* = −0.56, corrected *p* = 1.42 × 10^−8^; ACE‐III: *β* = 0.65, corrected *p* = 4.21 × 10^−11^; diagnosis: OR = 0.147, corrected *p* = 7.56 × 10^−10^), although all four lobes were significantly associated. In our analyses, many tract‐specific WMH volumes associated with clinical phenotypes to a similar or greater extent than total WMH volume or even the periventricular‐deep subclassification, supporting the literature on the relevance of WMH regional distributions (Biesbroek et al. 2016, 2024; Veldsman et al. 2020). The peak width of the skeletonised mean diffusivity (PSMD) was strongly associated with age, cognition and diagnoses, with effect sizes similar to those reported by Satizabal et al. (2020). Our findings extend the evidence supporting the use of this simple summary measure of small vessel disease to a memory clinic setting (Baykara et al. 2016; Deary et al. 2019).

In keeping with the literature on cortical mean diffusivity (MD) (Douaud et al. 2011, 2022; Illán‐Gala et al. 2019; Montal et al. 2021; Weston et al. 2020), we also observed associations with age (*p* = 6), cognition (*p* = 8), and diagnosis (*p* = 4), supporting the potential of MD to detect microstructural cortical changes in a memory clinic population. Although cortical mode of anisotropy (MO) is less common, we observed significant associations with MO in the bilateral supramarginal gyri (cognition and diagnosis) and right amygdala (diagnosis), supporting the ability of this measure to also detect dementia‐related changes. Some tract volumes were significantly associated with cognition (left corticospinal tract and major fornix) and diagnoses (left corticospinal tract, major fornix, and left medial lemniscus), but the directionality of these associations was inconsistent. Like many dMRI‐derived measures, changes in tract volumes are less specific, being influenced by both atrophy and microstructural changes, but nevertheless they can highlight tract‐specific atrophy patterns or flag poorer‐quality reconstructions (de Groot et al. 2015). Consistent with the literature on the cholinergic systems in dementia (Bohnen et al. 2018; Lagarde et al. 2024; Schumacher et al. 2022), we found that volumes of the Nucleus basalis of Meynert (NBM) bilaterally correlated with dementia diagnosis and age. The left NBM volume also associated with cognition. These associations were not as strong as with many other volumetric IDPs, but they may nevertheless hold value for differential diagnosis and prognosis.

In this work, we present a pragmatic statistical approach to enable data‐driven exploratory research in the clinical setting. Large sample sizes may be needed to reliably detect associations in the style of big data analyses (Marek et al. 2022), but without proper adjustments these sample sizes also inflate the risks of spurious associations. Indeed, moderate sample sizes may also be sufficient to detect associations with larger true effect sizes (Spisak, Bingel, and Wager 2023). As opposed to big data studies which may include hundreds of covariates (Alfaro‐Almagro et al. 2021), here we employ a core set of covariates more in line with clinical settings. We utilise hierarchical FDR‐correction by first correcting for comparisons across MRI modalities and then within modality using the Benjamini‐Hochberg procedure applied to those that survive the Simes test (Benjamini and Hochberg 1995; Yekutieli 2008). With hierarchical FDR control, we mitigate the risks associated with non‐independent analyses in small sample sizes (Nichols and Poline 2009; Vul et al. 2009; Yarkoni 2009). This novel application in a memory clinic setting enables data‐driven exploration of high‐dimensional neuroimaging data without relying on the select few large cohort studies.

A number of methodological limitations need to be considered when interpreting this study. Although this enhanced pipeline includes integrated QC for some modalities (T1, T2‐FLAIR, dMRI, and rfMRI), more work is needed to develop appropriate QC metrics and tools for swMRI, QSM, and ASL. For the modalities with integrated QC, it is important to note that the IQMs are no‐reference measures (i.e., without a ground truth). Flagging outliers was used as common criterion across IQMs across modalities, but this may not be necessarily the best method to detect quality deviations. Automated classifiers using IQMs as features do exist, but they are mostly for T1‐weighted scans, and classification accuracy varies substantially on clinical datasets (Bhalerao et al. 2024). When using traditional cut‐offs for DSE plots, our rfMRI QC reveals that some further optimisation may be required for this memory clinic population, but it remains unclear whether different thresholds may be more appropriate for this population, as no dataset was deemed unusable after further visual inspection. In this work, we employed previously proposed adaptations, but work is ongoing to evaluate alternatives for hippocampal segmentation (Sghirripa et al. 2024). Regarding surface‐based analyses, additional automated QC (e.g., Qoala‐T—Klapwijk et al. 2019) and optimisation of Freesurfer outputs may also be warranted, especially in this clinical population with substantial WMHs (Oi et al. 2023).

In addition, although our selection of dementia‐informed IDPs is non‐exhaustive, they demonstrate that the UKB IDPs may underrepresent the full picture when there is a clear clinical question. Compared to big data studies, the sample size here affects our ability to accurately detect associations. However, in this study we demonstrate the utility of using hierarchical FDR for detecting both known and novel associations in the presence of limited data. Future work could expand on the set of essential covariates used here and include analyses with greater sensitivity to non‐linear associations. Nevertheless, the findings here serve to demonstrate the possibilities enabled by integrating UK Biobank‐aligned imaging and analyses, enriched for sensitivity to dementia‐related changes, directly into clinical settings. Further work will aim to address the extent to which these IDPs capture unique variability and inform differential diagnosis and precision phenotyping in a memory clinic setting.

This enriched and integrated quality control‐analysis pipeline for memory clinics offers possibilities in both clinical and research spheres. In clinical practice, the combination of established and novel quantitative measures has the potential to significantly improve the accuracy of differential diagnoses and predicted responses to treatment. In contrast to UK Biobank where participants are mostly healthy, this study better describes the brain alterations associated with dementia in a sample of memory clinic attendees. Although a future direct comparison with UK Biobank will need to take into account the fact that the two datasets were acquired on different hardware and that there is an age difference between the two populations, the alignment of our image acquisition and processing pipeline with UKB provides a foundation for future harmonisation efforts with high potential for personalised medicine and normative modelling. We currently present a rich dataset of deeply‐phenotyped, unselected memory clinic patients representative of our region, recognising that this population is drawn from an area with relatively little diversity. However, wider use of this dementia‐enhanced pipeline offers avenues for more inclusive research in real‐world memory clinic populations. This analysis‐QC pipeline is also well‐suited as a framework to plug‐in and pilot additional analyses suited to different clinical applications.

## Author Contributions

**Grace Gillis:** conceptualisation, data curation, formal analysis, investigation, methodology, software, validation, visualisation, writing – original draft. **Gaurav Bhalerao:** methodology, software, validation, writing – review and editing. **Jasmine Blane:** investigation, project administration, writing – review and editing. **Robert Mitchell:** investigation, writing – review and editing. **Pieter M. Pretorius:** investigation, writing – review and editing. **Celeste McCracken:** methodology, writing – review and editing. **Thomas E. Nichols:** methodology, writing – review and editing. **Stephen M. Smith:** methodology, software, funding acquisition, writing – review and editing. **Karla L. Miller:** methodology, software, funding acquisition, writing – review and editing. **Fidel Alfaro‐Almagro:** methodology, software, writing – review and editing. **Vanessa Raymont:** conceptualisation, resources, funding acquisition, supervision, writing – review and editing. **Lola Martos:** conceptualisation, resources, funding acquisition, writing – review and editing. **Clare E. Mackay:** conceptualisation, methodology, resources, funding acquisition, supervision, writing – review and editing. **Ludovica Griffanti:** conceptualisation, methodology, resources, funding acquisition, supervision, writing – review and editing.

## Conflicts of Interest

C.E.M. is a cofounder and shareholder of Exprodo Software, which was used to develop the OBHC database. C.E.M. serves on a Biogen Brain Health Consortium (unpaid). No other competing interests to report.

## Supporting information


Data S1.

## Data Availability

The complete OBHC MRI protocol and scanning procedure is available through the WIN MR Protocols Database at: https://open.win.ox.ac.uk/protocols/stable/6974395a‐3745–4861‐b8cc‐1887e787d1c4 (O'Donoghue et al. 2022). The UKB‐dementia pipeline presented here and used for this analysis is openly available (https://git.fmrib.ox.ac.uk/open‐science/analysis/brain‐health‐clinic‐mri) along with the original UK Biobank brain MRI analysis pipeline (https://git.fmrib.ox.ac.uk/falmagro/uk_biobank_pipeline_v_1.5/‐/tree/master). Additional scripts used for OBHC analyses are also available (https://git.fmrib.ox.ac.uk/gillisc/01_bhc_imaging.git). Interactive versions of the figures are available (https://users.ox.ac.uk/~scat8503/). The MRI data presented in this paper will be available via the Dementias Platform UK (https://portal.dementiasplatform.uk/CohortDirectory/Item?fingerPrintID=BHC), and access will be granted through an application process, reviewed by the OBHC Data Access Group. The OBHC Data Access Group will start accepting applications to access OBHC data upon publication of the present work. Data will continue to be released in batches as the OBHC progresses in order to minimise the risk of participant identification.

## References

[hbm70151-bib-0001] Afyouni, S. , and T. E. Nichols . 2018. “Insight and Inference for DVARS.” NeuroImage 172: 291–312. 10.1016/j.neuroimage.2017.12.098. 29307608 PMC5915574

[hbm70151-bib-0002] Alfaro‐Almagro, F. , M. Jenkinson , N. K. Bangerter , et al. 2018. “Image Processing and Quality Control for the First 10,000 Brain Imaging Datasets From UK Biobank.” NeuroImage 166: 400–424. 10.1016/j.neuroimage.2017.10.034. 29079522 PMC5770339

[hbm70151-bib-0003] Alfaro‐Almagro, F. , P. McCarthy , S. Afyouni , et al. 2021. “Confound Modelling in UK Biobank Brain Imaging.” NeuroImage 224: 117002. 10.1016/j.neuroimage.2020.117002. 32502668 PMC7610719

[hbm70151-bib-0004] Bastiani, M. , M. Cottaar , S. P. Fitzgibbon , et al. 2019. “Automated Quality Control for Within and Between Studies Diffusion MRI Data Using a Non‐Parametric Framework for Movement and Distortion Correction.” NeuroImage 184: 801–812. 10.1016/j.neuroimage.2018.09.073. 30267859 PMC6264528

[hbm70151-bib-0005] Baykara, E. , B. Gesierich , R. Adam , et al. 2016. “A Novel Imaging Marker for Small Vessel Disease Based on Skeletonization of White Matter Tracts and Diffusion Histograms.” Annals of Neurology 80, no. 4: 581–592. 10.1002/ana.24758. 27518166

[hbm70151-bib-0006] Beer, A. L. , T. Plank , and M. W. Greenlee . 2020. “Aging and Central Vision Loss: Relationship Between the Cortical Macro‐Structure and Micro‐Structure.” NeuroImage 212: 116670. 10.1016/j.neuroimage.2020.116670. 32088318

[hbm70151-bib-0007] Benjamini, Y. , and Y. Hochberg . 1995. “Controlling the False Discovery Rate: A Practical and Powerful Approach to Multiple Testing.” Journal of the Royal Statistical Society: Series B: Methodological 57, no. 1: 289–300. 10.1111/j.2517-6161.1995.tb02031.x.

[hbm70151-bib-0008] Besseling, R. M. H. , J. F. A. Jansen , G. M. Overvliet , et al. 2012. “Tract Specific Reproducibility of Tractography Based Morphology and Diffusion Metrics.” PLoS One 7, no. 4: e34125. 10.1371/journal.pone.0034125. 22485157 PMC3317780

[hbm70151-bib-0009] Bhalerao, G. , G. Gillis , M. Dembele , et al. 2024. “Automated Quality Control of T1‐Weighted Brain MRI Scans for Clinical Research: Methods Comparison and Design of a Quality Prediction Classifier.” medRxiv. 10.1101/2024.04.12.24305603. PMC12319838 40800787

[hbm70151-bib-0010] Biesbroek, J. M. , M. Coenen , C. DeCarli , et al. 2024. “Amyloid Pathology and Vascular Risk Are Associated With Distinct Patterns of Cerebral White Matter Hyperintensities: A Multicenter Study in 3132 Memory Clinic Patients.” Alzheimer's & Dementia 20, no. 4: 2980–2989. 10.1002/alz.13765. PMC11032573 38477469

[hbm70151-bib-0011] Biesbroek, J. M. , N. A. Weaver , S. Hilal , et al. 2016. “Impact of Strategically Located White Matter Hyperintensities on Cognition in Memory Clinic Patients With Small Vessel Disease.” PLoS One 11, no. 11: e0166261. 10.1371/journal.pone.0166261. 27824925 PMC5100905

[hbm70151-bib-0012] Bohnen, N. I. , M. J. Grothe , N. J. Ray , M. L. T. M. Müller , and S. J. Teipel . 2018. “Recent Advances in Cholinergic Imaging and Cognitive Decline—Revisiting the Cholinergic Hypothesis of Dementia.” Current Geriatrics Reports 7, no. 1: 1. 10.1007/s13670-018-0234-4. 29503795 PMC5831510

[hbm70151-bib-0013] Bolandzadeh, N. , J. C. Davis , R. Tam , T. C. Handy , and T. Liu‐Ambrose . 2012. “The Association Between Cognitive Function and White Matter Lesion Location in Older Adults: A Systematic Review.” BMC Neurology 12, no. 1: 126. 10.1186/1471-2377-12-126. 23110387 PMC3522005

[hbm70151-bib-0014] Borchert, R. J. , T. Azevedo , A. Badhwar , et al. 2023. “Artificial Intelligence for Diagnostic and Prognostic Neuroimaging in Dementia: A Systematic Review.” Alzheimer's & Dementia 19, no. 12: 5885–5904. 10.1002/alz.13412. 37563912

[hbm70151-bib-0015] Bozzali, M. , G. Giulietti , B. Basile , et al. 2011. “Damage to the Cingulum Contributes to Alzheimer's Disease Pathophysiology by Deafferentation Mechanism.” Human Brain Mapping 33, no. 6: 1295–1308. 10.1002/hbm.21287. 21520352 PMC6870125

[hbm70151-bib-0016] Collins, D. L. , C. J. Holmes , T. M. Peters , and A. C. Evans . 1995. “Automatic 3‐D Model‐Based Neuroanatomical Segmentation.” Human Brain Mapping 3, no. 3: 190–208. 10.1002/hbm.460030304.

[hbm70151-bib-0017] Deary, I. J. , S. J. Ritchie , S. Muñoz Maniega , et al. 2019. “Brain Peak Width of Skeletonized Mean Diffusivity (PSMD) and Cognitive Function in Later Life.” Frontiers in Psychiatry 10: 524. 10.3389/fpsyt.2019.00524. 31402877 PMC6676305

[hbm70151-bib-0018] Douaud, G. , S. Jbabdi , T. E. J. Behrens , et al. 2011. “DTI Measures in Crossing‐Fibre Areas: Increased Diffusion Anisotropy Reveals Early White Matter Alteration in MCI and Mild Alzheimer's Disease.” NeuroImage 55, no. 3: 880–890. 10.1016/j.neuroimage.2010.12.008. 21182970 PMC7116583

[hbm70151-bib-0019] Douaud, G. , S. Lee , F. Alfaro‐Almagro , et al. 2022. “SARS‐CoV‐2 Is Associated With Changes in Brain Structure in UK Biobank.” Nature (London) 604, no. 7907: 697–707. 10.1038/s41586-022-04569-5. 35255491 PMC9046077

[hbm70151-bib-0020] Douaud, G. , R. A. L. Menke , A. Gass , et al. 2013. “Brain Microstructure Reveals Early Abnormalities More Than Two Years Prior to Clinical Progression From Mild Cognitive Impairment to Alzheimer's Disease.” Journal of Neuroscience 33, no. 5: 2147–2155. 10.1523/JNEUROSCI.4437-12.2013. 23365250 PMC6571077

[hbm70151-bib-0021] Egle, M. , S. Hilal , A. M. Tuladhar , et al. 2022. “Prediction of Dementia Using Diffusion Tensor MRI Measures: The OPTIMAL Collaboration.” Journal of Neurology, Neurosurgery, and Psychiatry 93, no. 1: 14–23. 10.1136/jnnp-2021-326571. 34509999

[hbm70151-bib-0022] Esteban, O. , D. Birman , M. Schaer , O. O. Koyejo , R. A. Poldrack , and K. J. Gorgolewski . 2017. “MRIQC: Advancing the Automatic Prediction of Image Quality in MRI From Unseen Sites.” PLoS One 12, no. 9: e0184661. 10.1371/journal.pone.0184661. 28945803 PMC5612458

[hbm70151-bib-0023] Fazekas, F. , J. Chawluk , A. Alavi , H. Hurtig , and R. Zimmerman . 1987. “MR Signal Abnormalities at 1.5 T in Alzheimer's Dementia and Normal Aging.” American Journal of Roentgenology 149, no. 2: 351–356. 10.2214/ajr.149.2.351. 3496763

[hbm70151-bib-0024] Griffanti, L. , G. Gillis , M. C. O'Donoghue , et al. 2022. “Adapting UK Biobank Imaging for Use in a Routine Memory Clinic Setting: The Oxford Brain Health Clinic.” NeuroImage: Clinical 36: 103273. 10.1016/j.nicl.2022.103273. 36451375 PMC9723313

[hbm70151-bib-0025] Griffanti, L. , M. Jenkinson , S. Suri , et al. 2018. “Classification and Characterization of Periventricular and Deep White Matter Hyperintensities on MRI: A Study in Older Adults.” NeuroImage 170: 174–181. 10.1016/j.neuroimage.2017.03.024. 28315460

[hbm70151-bib-0027] Griffanti, L. , G. Zamboni , A. Khan , et al. 2016. “BIANCA (Brain Intensity AbNormality Classification Algorithm): A New Tool for Automated Segmentation of White Matter Hyperintensities.” NeuroImage 141: 191–205. 10.1016/j.neuroimage.2016.07.018. 27402600 PMC5035138

[hbm70151-bib-0028] de Groot, M. , M. A. Ikram , S. Akoudad , et al. 2015. “Tract‐Specific White Matter Degeneration in Aging: The Rotterdam Study.” Alzheimer's & Dementia 11, no. 3: 321–330. 10.1016/j.jalz.2014.06.011. 25217294

[hbm70151-bib-0029] Haidar, H. , R. E. Majzoub , S. Hajeer , and L. A. Abbas . 2023. “Arterial Spin Labeling (ASL‐MRI) Versus Fluorodeoxyglucose‐PET (FDG‐PET) in Diagnosing Dementia: A Systematic Review and Meta‐Analysis.” BMC Neurology 23, no. 1: 385. 10.1186/s12883-023-03432-y. 37875879 PMC10594722

[hbm70151-bib-0030] Harms, M. P. , L. H. Somerville , B. M. Ances , et al. 2018. “Extending the Human Connectome Project Across Ages: Imaging Protocols for the Lifespan Development and Aging Projects.” NeuroImage 183: 972–984. 10.1016/j.neuroimage.2018.09.060. 30261308 PMC6484842

[hbm70151-bib-0031] Harper, L. , F. Barkhof , N. C. Fox , and J. M. Schott . 2015. “Using Visual Rating to Diagnose Dementia: A Critical Evaluation of MRI Atrophy Scales.” Journal of Neurology, Neurosurgery & Psychiatry 86, no. 11: 1225–1233. 10.1136/jnnp-2014-310090. 25872513

[hbm70151-bib-0032] Harper, L. , F. Bouwman , E. J. Burton , et al. 2017. “Patterns of Atrophy in Pathologically Confirmed Dementias: A Voxelwise Analysis.” Journal of Neurology, Neurosurgery & Psychiatry 88, no. 11: 908–916. 10.1136/jnnp-2016-314978. 28473626 PMC5740544

[hbm70151-bib-0033] Hirschfeld, L. R. , R. Deardorff , E. J. Chumin , et al. 2023. “White Matter Integrity Is Associated With Cognition and Amyloid Burden in Older Adult Koreans Along the Alzheimer's Disease Continuum.” Alzheimer's Research & Therapy 15, no. 1: 218. 10.1186/s13195-023-01369-5. PMC10725037 38102714

[hbm70151-bib-0034] Illán‐Gala, I. , V. Montal , S. Borrego‐Écija , et al. 2019. “Cortical Microstructure in the Behavioural Variant of Frontotemporal Dementia: Looking Beyond Atrophy.” Brain: A Journal of Neurology 142, no. 4: 1121–1133. 10.1093/brain/awz031. 30906945 PMC6439330

[hbm70151-bib-0035] Jack, C. R. , D. A. Bennett , K. Blennow , et al. 2016. “A/T/N: An Unbiased Descriptive Classification Scheme for Alzheimer Disease Biomarkers.” Neurology 87, no. 5: 539–547. 10.1212/WNL.0000000000002923. 27371494 PMC4970664

[hbm70151-bib-0036] Klapwijk, E. T. , F. van de Kamp , M. van der Meulen , S. Peters , and L. M. Wierenga . 2019. “Qoala‐T: A Supervised‐Learning Tool for Quality Control of FreeSurfer Segmented MRI Data.” NeuroImage 189: 116–129. 10.1016/j.neuroimage.2019.01.014. 30633965

[hbm70151-bib-0037] Lagarde, J. , P. Olivieri , M. Tonietto , et al. 2024. “Combined In Vivo MRI Assessment of Locus Coeruleus and Nucleus Basalis of Meynert Integrity in Amnestic Alzheimer's Disease, Suspected‐LATE and Frontotemporal Dementia.” Alzheimer's Research & Therapy 16, no. 1: 97. 10.1186/s13195-024-01466-z. PMC11067144 38702802

[hbm70151-bib-0038] Langbaum, J. B. , J. Zissimopoulos , R. Au , et al. 2023. “Recommendations to Address Key Recruitment Challenges of Alzheimer's Disease Clinical Trials.” Alzheimer's & Dementia 19, no. 2: 696–707. 10.1002/alz.12737. PMC9911558 35946590

[hbm70151-bib-0039] Lee, S. , J. D. Bijsterbosch , F. A. Almagro , et al. 2023. “Amplitudes of Resting‐State Functional Networks—Investigation Into Their Correlates and Biophysical Properties.” NeuroImage 265: 119779. 10.1016/j.neuroimage.2022.119779. 36462729 PMC10933815

[hbm70151-bib-0040] Lim, A. C. , L. L. Barnes , G. H. Weissberger , et al. 2023. “Quantification of Race/Ethnicity Representation in Alzheimer's Disease Neuroimaging Research in the USA: A Systematic Review.” Communications Medicine 3, no. 1: 1. 10.1038/s43856-023-00333-6. 37491471 PMC10368705

[hbm70151-bib-0041] Liu, Y. , S. Mazumdar , and P. A. Bath . 2023. “An Unsupervised Learning Approach to Diagnosing Alzheimer's Disease Using Brain Magnetic Resonance Imaging Scans.” International Journal of Medical Informatics 173: 105027. 10.1016/j.ijmedinf.2023.105027. 36921480

[hbm70151-bib-0042] Low, A. , E. Mak , J. D. Stefaniak , et al. 2020. “Peak Width of Skeletonized Mean Diffusivity as a Marker of Diffuse Cerebrovascular Damage.” Frontiers in Neuroscience 14: 238. 10.3389/fnins.2020.00238. 32265640 PMC7096698

[hbm70151-bib-0043] Marek, S. , B. Tervo‐Clemmens , F. J. Calabro , et al. 2022. “Reproducible Brain‐Wide Association Studies Require Thousands of Individuals.” Nature 603, no. 7902: 654–660. 10.1038/s41586-022-04492-9. 35296861 PMC8991999

[hbm70151-bib-0044] Mazziotta, J. , A. Toga , A. Evans , et al. 2001. “A Probabilistic Atlas and Reference System for the Human Brain: International Consortium for Brain Mapping (ICBM).” Philosophical Transactions of the Royal Society of London. Series B: Biological Sciences 356, no. 1412: 1293–1322. 10.1098/rstb.2001.0915. 11545704 PMC1088516

[hbm70151-bib-0045] McCracken, C. , Z. Raisi‐Estabragh , M. Veldsman , et al. 2022. “Multi‐Organ Imaging Demonstrates the Heart‐Brain‐Liver Axis in UK Biobank Participants.” Nature Communications 13, no. 1: 7839. 10.1038/s41467-022-35321-2. PMC9772225 36543768

[hbm70151-bib-0046] Miller, K. L. , F. Alfaro‐Almagro , N. K. Bangerter , et al. 2016. “Multimodal Population Brain Imaging in the UK Biobank Prospective Epidemiological Study.” Nature Neuroscience 19, no. 11: 1523–1536. 10.1038/nn.4393. 27643430 PMC5086094

[hbm70151-bib-0047] Montal, V. , E. Vilaplana , J. Pegueroles , et al. 2021. “Biphasic Cortical Macro‐ and Microstructural Changes in Autosomal Dominant Alzheimer's Disease.” Alzheimer's & Dementia 17, no. 4: 618–628. 10.1002/alz.12224. PMC8043974 33196147

[hbm70151-bib-0048] Mori, S. , K. Oishi , H. Jiang , et al. 2008. “Stereotaxic White Matter Atlas Based on Diffusion Tensor Imaging in an ICBM Template.” NeuroImage 40, no. 2: 570–582. 10.1016/j.neuroimage.2007.12.035. 18255316 PMC2478641

[hbm70151-bib-0049] National Institute for Health and Care Excellence (NICE) . 2018. Overview | Dementia: Assessment, Management and Support for People Living With Dementia and Their Carers | Guidance | NICE. NICE. https://www.nice.org.uk/guidance/ng97. 30011160

[hbm70151-bib-0050] Nichols, T. E. , and J.‐B. Poline . 2009. “Commentary on Vul Et Al.'s (2009) ‘Puzzlingly High Correlations in fMRI Studies of Emotion, Personality, and Social Cognition’.” Perspectives on Psychological Science: A Journal of the Association for Psychological Science 4, no. 3: 291–293. 10.1111/j.1745-6924.2009.01126.x. 26158965

[hbm70151-bib-0051] O'Donoghue, M. C. , J. Blane , G. Gillis , et al. 2023. “Oxford Brain Health Clinic: Protocol and Research Database.” BMJ Open 13, no. 8: e067808. 10.1136/bmjopen-2022-067808. PMC10407419 37541753

[hbm70151-bib-0052] O'Donoghue, M. C. , J. Blane , J. Semple , et al. 2022. “WIN MR Protocol: Oxford Brain Health Centre (2019_102_BHC).” 10.5281/zenodo.6598036.

[hbm70151-bib-0053] Oi, Y. , M. Hirose , H. Togo , et al. 2023. “Identifying and Reverting the Adverse Effects of White Matter Hyperintensities on Cortical Surface Analyses.” NeuroImage 281: 120377. 10.1016/j.neuroimage.2023.120377. 37714391

[hbm70151-bib-0054] Pemberton, H. G. , L. A. M. Zaki , O. Goodkin , et al. 2021. “Technical and Clinical Validation of Commercial Automated Volumetric MRI Tools for Dementia Diagnosis—A Systematic Review.” Neuroradiology 63, no. 11: 1773–1789. 10.1007/s00234-021-02746-3. 34476511 PMC8528755

[hbm70151-bib-0055] Penalba‐Sánchez, L. , P. Oliveira‐Silva , A. L. Sumich , and I. Cifre . 2023. “Increased Functional Connectivity Patterns in Mild Alzheimer's Disease: A rsfMRI Study.” Frontiers in Aging Neuroscience 14: 1037347. 10.3389/fnagi.2022.1037347. 36698861 PMC9869068

[hbm70151-bib-0056] Petersen, R. C. , P. S. Aisen , L. A. Beckett , et al. 2010. “Alzheimer's Disease Neuroimaging Initiative (ADNI).” Neurology 74, no. 3: 201–209. 10.1212/WNL.0b013e3181cb3e25. 20042704 PMC2809036

[hbm70151-bib-0057] Peterson, R. A. , and J. E. Cavanaugh . 2020. “Ordered Quantile Normalization: A Semiparametric Transformation Built for the Cross‐Validation Era.” Journal of Applied Statistics 47, no. 13–15: 2312–2327. 10.1080/02664763.2019.1630372. 35707424 PMC9042069

[hbm70151-bib-0058] Peterson, R. A. 2021. “Finding Optimal Normalizing Transformations via Bestnormalize.” R Journal 13, no. 1: 310–329. 10.32614/RJ-2021-041.

[hbm70151-bib-0059] Rabinovici, G. D. , W. W. Seeley , E. J. Kim , et al. 2007. “Distinct MRI Atrophy Patterns in Autopsy‐Proven Alzheimer's Disease and Frontotemporal Lobar Degeneration.” American Journal of Alzheimer's Disease and Other Dementias 22, no. 6: 474–488. 10.1177/1533317507308779. PMC2443731 18166607

[hbm70151-bib-0060] Satizabal, C. L. , A. S. Beiser , P. Maillard , et al. 2020. “PSMD, a Novel Marker of Small Vessel Disease, and Its Association With Cognitive Function in the Community.” Alzheimer's & Dementia 16, no. S5: e041993. 10.1002/alz.041993.

[hbm70151-bib-0061] Scheltens, P. , L. J. Launer , F. Barkhof , H. C. Weinstein , and W. A. van Gool . 1995. “Visual Assessment of Medial Temporal Lobe Atrophy on Magnetic Resonance Imaging: Interobserver Reliability.” Journal of Neurology 242, no. 9: 557–560. 10.1007/BF00868807. 8551316

[hbm70151-bib-0062] Schumacher, J. , N. J. Ray , C. A. Hamilton , et al. 2022. “Cholinergic White Matter Pathways in Dementia With Lewy Bodies and Alzheimer's Disease.” Brain: A Journal of Neurology 145, no. 5: 1773–1784. 10.1093/brain/awab372. 34605858 PMC9166545

[hbm70151-bib-0063] Sghirripa, S. , G. Bhalerao , L. Griffanti , et al. 2024. “Evaluating Traditional, Deep Learning, and Subfield Methods for Automatically Segmenting the Hippocampus From MRI.” medRxiv. 10.1101/2024.08.06.24311530. PMC11947432 40143669

[hbm70151-bib-0064] Smith, S. , F. Alfaro‐Almagro , and K. Miller . 2022. “UK Biobank Brain Imaging Documentation.” https://biobank.ctsu.ox.ac.uk/crystal/crystal/docs/brain_mri.pdf.

[hbm70151-bib-0065] Spisak, T. , U. Bingel , and T. D. Wager . 2023. “Multivariate BWAS Can Be Replicable With Moderate Sample Sizes.” Nature 615, no. 7951: E4–E7. 10.1038/s41586-023-05745-x. 36890392 PMC9995263

[hbm70151-bib-0066] Teipel, S. , and M. J. Grothe . 2023. “MRI‐Based Basal Forebrain Atrophy and Volumetric Signatures Associated With Limbic TDP‐43 Compared to Alzheimer's Disease Pathology.” Neurobiology of Disease 180: 106070. 10.1016/j.nbd.2023.106070. 36898615

[hbm70151-bib-0067] Veldsman, M. , P. Kindalova , M. Husain , I. Kosmidis , and T. E. Nichols . 2020. “Spatial Distribution and Cognitive Impact of Cerebrovascular Risk‐Related White Matter Hyperintensities.” NeuroImage: Clinical 28: 102405. 10.1016/j.nicl.2020.102405. 32971464 PMC7511743

[hbm70151-bib-0068] Vemuri, P. , D. T. Jones , and C. R. Jack . 2012. “Resting State Functional MRI in Alzheimer's Disease.” Alzheimer's Research & Therapy 4, no. 1: 2. 10.1186/alzrt100. PMC3471422 22236691

[hbm70151-bib-0069] Visser, P. J. , F. R. J. Verhey , P. A. M. Hofman , P. Scheltens , and J. Jolles . 2002. “Medial Temporal Lobe Atrophy Predicts Alzheimer's Disease in Patients With Minor Cognitive Impairment.” Journal of Neurology, Neurosurgery & Psychiatry 72, no. 4: 491–497. 10.1136/jnnp.72.4.491. 11909909 PMC1737837

[hbm70151-bib-0070] Vul, E. , C. Harris , P. Winkielman , and H. Pashler . 2009. “Puzzlingly High Correlations in fMRI Studies of Emotion, Personality, and Social Cognition.” Perspectives on Psychological Science: A Journal of the Association for Psychological Science 4, no. 3: 274–290. 10.1111/j.1745-6924.2009.01125.x. 26158964

[hbm70151-bib-0071] Warrington, S. , K. L. Bryant , A. A. Khrapitchev , et al. 2020. “XTRACT—Standardised Protocols for Automated Tractography in the Human and Macaque Brain.” NeuroImage 217: 116923. 10.1016/j.neuroimage.2020.116923. 32407993 PMC7260058

[hbm70151-bib-0072] Weston, P. S. J. , T. Poole , J. M. Nicholas , et al. 2020. “Measuring Cortical Mean Diffusivity to Assess Early Microstructural Cortical Change in Presymptomatic Familial Alzheimer's Disease.” Alzheimer's Research & Therapy 12: 112. 10.1186/s13195-020-00679-2. PMC7499910 32943095

[hbm70151-bib-0073] Woodworth, D. C. , N. Sheikh‐Bahaei , K. A. Scambray , et al. 2022. “Dementia Is Associated With Medial Temporal Atrophy Even After Accounting for Neuropathologies.” Brain Communications 4, no. 2: fcac052. 10.1093/braincomms/fcac052. 35350552 PMC8952251

[hbm70151-bib-0074] Xiao, Y. , J. Wang , K. Huang , L. Gao , S. Yao , and Initiative, for the A. D. N . 2023. “Progressive Structural and Covariance Connectivity Abnormalities in Patients With Alzheimer's Disease.” Frontiers in Aging Neuroscience 14: 1064667. 10.3389/fnagi.2022.1064667. 36688148 PMC9853893

[hbm70151-bib-0075] Yarkoni, T. 2009. “Big Correlations in Little Studies: Inflated fMRI Correlations Reflect Low Statistical Power‐Commentary on Vul Et Al. (2009).” Perspectives on Psychological Science: A Journal of the Association for Psychological Science 4, no. 3: 294–298. 10.1111/j.1745-6924.2009.01127.x. 26158966

[hbm70151-bib-0076] Yekutieli, D. 2008. “Hierarchical False Discovery Rate‐Controlling Methodology.” Journal of the American Statistical Association 103, no. 481: 309–316.

[hbm70151-bib-0077] Zaborszky, L. , L. Hoemke , H. Mohlberg , A. Schleicher , K. Amunts , and K. Zilles . 2008. “Stereotaxic Probabilistic Maps of the Magnocellular Cell Groups in Human Basal Forebrain.” NeuroImage 42, no. 3: 1127–1141. 10.1016/j.neuroimage.2008.05.055. 18585468 PMC2577158

