# Supplementary material for: From Big Data to the Clinic: Methodological and Statistical Enhancements to Implement the UK Biobank Imaging Framework in a Memory Clinic
Source: Hum Brain Mapp. 2025 Feb 19;46(3):e70151. doi: 10.1002/hbm.70151 (PMC11837031; doi:10.1002/hbm.70151)
Supplement: Supplementary file 1 — Data S1. [file HBM-46-e70151-s001.pdf]

Supplementary Materials

Supplementary Section 1: MRI Protocol 2
Supplementary Section 2: T2* IDPs 3
Supplementary Section 3: IQMs 4
Supplementary Section 4: Visual QC 5
Supplementary Section 5: Associations with age without ACE-III adjustment 7
Supplementary Section 6: Associations with age 9
Supplementary Section 7: Associations with age controlling for volume 19
Supplementary Section 8: Associations with ACE-III 21
Supplementary Section 9: Associations with cognition controlling for volume 27
Supplementary Section 10: Associations with diagnosis 29

Supplementary Section 1: MRI Protocol

Supplementary Table 1: MRI protocol. MPRAGE = Magnetization Prepared RApid Gradient Echo; FLAIR = Fluid-attenuated inversion recovery; SPACE = Sampling Perfection with Application optimized Contrasts using different flip angle Evolution; ASL = Arterial Spin Labeling; PCASL = pseudo-continuous ASL; TR = Repetition time; TE = Echo time; TI = Inversion time; R = In-plane acceleration factor; MB = Multi-band acceleration factor; α = flip angle; PLD = postlabeling delay; M0 = equilibrium magnetization, required for ASL quantification. Full MRI protocols are available online (BHC: https://open.win.ox.ac.uk/protocols/stable/6974395a-3745–4861-b8cc-1887e787d1c4 (O'Donoghue et al., 2022)); UKB: https://open.win.ox.ac.uk/protocols/stable/d2b297c3-4a4f-4fde-9b7c-7a8ae6e5fa83).

**Modality**	**Acquisition time**	**Resolution**	**Matrix**	**Key Parameters**	**UKB Match**
**BHC core clinical protocol**	16:29				
Localiser	0:14				No
Diffusion MRI (dMRI 3-scan trace)	0:43	0.8x0.8x4 mm	260x260 x27	TR = 3800 ms , 3 dirs, b = 0, 1000 s/mm^2	N/A
Susceptibility -weighted MRI (swMRI)	4:46	0.8x0.8x1.5 mm	256x288 x80	TE1/TE2/TR = 10/20/30 ms, R = 2	Adapted
T1 (MPRAGE)	4:54	1.0x1.0x1.0 mm	256x256 x208	TI/TR = 800/2 000 ms, R = 2	Exact
T2-FLAIR (SPACE)	5:52	1.0x1.0x1.0 5 mm	256x256 x192	TI/TR = 1800/ 5000 ms, R = 2	Exact
**BHC research protocol**	21:17				
ASL localiser (time of flight)	0:42				N/A
Diffusion MRI (dMRI)	7:08	2.0x2.0x2.0 mm	104x104 x72	TR = 3600 ms , 50 dirs/shell, b = 0,1000,20 00 s/mm2, MB = 3, blip-reversed b = 0	Exact
Arterial spin labelling (PCASL)	7:17	3.4x3.4x4.5 mm	64x64x2 4	TE/TR = 14/4 400 ms, tag = 1400 ms , seven PLDs = 250,5 00…1750 ms, 1 M0 calibration image	Not in UKB initial protocol
Resting state fMRI (rfMRI)	6:10	2.4x2.4x2.4 mm	88x88x6 4	TE/TR = 39/7 35 ms, α = 52°, MB = 8	Exact

Supplementary Section 2: T2* IDPs

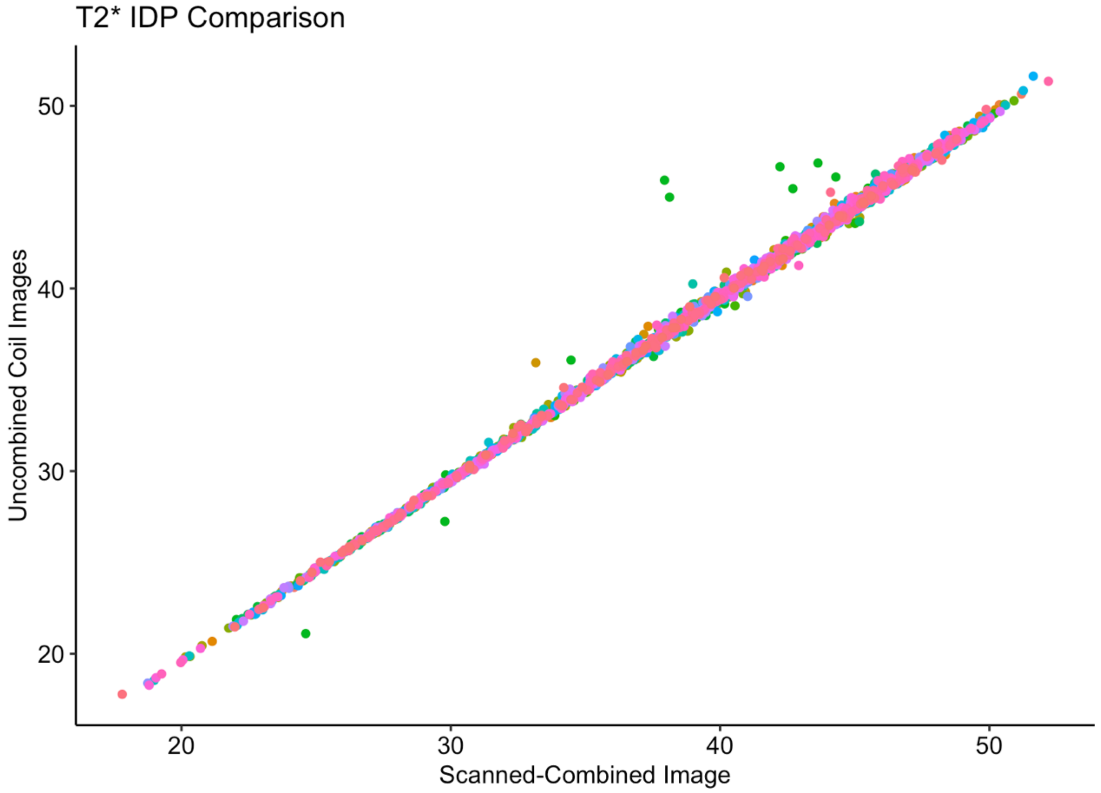

Supplementary Figure 1: Comparison of T2 IDPs derived from the scanner-combined and the separate coil images, showing their equivalence. IDPs from one scan (in green) differed and were excluded from further analysis.*

Supplementary Section 3: IQMs

Supplementary Table 2: Image quality metrics (IQMs) flagged by automated quality control. N is the number of scans flagged as outliers (>1.5 IQR away from Q1/Q3) for each IQM. DSE decomposition, which was run separately on the raw and processed rfMRI data, returns the whole (or total) D-var, S-var, and E-var sum-of-squares decomposition as well the global components within each of these. For details about the individual IQMs, please refer to Esteban et al. (2017) for MRIQC, Afyouni and Nichols (2018) for DSE and Bastiani et al. (2019) for EDDY QUAD.

Modality	Flagged Image Quality Metrics (IQMs)
**T1 (MRIQC)**	CJV (N=1); CNR (N=1); SNR_CSF (N=0); **SNR_GM (N= 5)**; **SNR_WM (N=5)**; **QI2 (N=7)**; EFC (N=2); **FBER (N=5)**; INU_med (N=1); INU_range (N=1); WM2MAX (N=4)
**T2-FLAIR (MRIQC)**	**CNR (N=6)**; SNR_CSF (N=1); SNR_GM (N=3); QI2 (N=3); EFC (N=3); FBER (N=3); INU_med (N=3); **WM2MAX (N=18)**
**dMRI (EDDY QUAD)**	SNR (N=3); CNR_b1000 (N=0); CNR_b1997 (N=1); **absolute motion (N=8)**; **relative motion (N=6)**; susceptibility (N=3); **total % outliers (N=11)**
**rfMRI (MRIQC, DSE decomposition)**	EFC (N=0); FBER (N=1); SNR (N=2); tSNR (N=2); **GSR_x (N=7)**; GSR_y (N=0); **GCOR (N=10)**; **AOR (N=6)**; AQI (N=4); raw data % d-Var (whole – N=0; **global – N=8**); raw data % s-Var (whole – N=0; **global – N=7**); processed data % d-Var (whole – N=1; **global – N=8**); processed data % s-Var (whole – N=1; **global – N=10**)

Supplementary Section 4: Visual QC

Supplementary Table 3: Detailed results of visual inspection of flagged scans. One patient dataset, flagged for T1-weighted and T2-FLAIR, could not be processed or visually inspected due to a scan archive failure (excluded from subsequent analyses).

Modality (N inspected)	Output visually inspected*	Quality		
		High (N)	Medium (N)	Low (N)
**T1-weighted (31)**	*Corrected SIENAX segmentation*	87.1% (27)	3.2% (1)	6.5% (2)
	Corrected FIRST segmentation	71.0% (22)	3.2% (1)	22.6% (7)
**T2-FLAIR (35)**	*BIANCA segmentation*	68.6% (24)	25.7% (9)	2.9% (1)
**dMRI (24)**	*Tractography*	70.8% (17)	29.2% (7)	0% (0)
		**S-var exceeds 75%**		
		**Never (N)**	**Rarely (N)**	**Often (N)**
**rfMRI (34)**	*DSE plots*	2.9% (1)	29.4% (10)	67.6% (23)

*Segmentations from T1 (corrected FIRST and SIENAX) and T2-FLAIR (BIANCA) were visually inspected by one of 3 raters, all trained on example scans to follow similar criteria (e.g., alignment with tissue-type boundaries, mislocalisation of FIRST subcortical structures, over or under-segmentation of WMHs with different image qualities). Thresholded tractography segmentations from dMRI were inspected by one rater to ensure a good overlap with anatomical landmarks. The DSE decomposition plots of the preprocessed data from flagged rfMRI scans were visually inspected by one rater noting the frequency of S-var exceeding 75%.

Noticing that the 75% threshold for the %S-var proposed in Afyouni and Nichols (2018) was exceeded at least occasionally in most of the preprocessed OBHC scans, we further investigated other DSE metrics analysed in Afyouni and Nichols (2018). We compared the mean %A-var from raw and processed scans as an indication of rfMRI processing overall performance. We were able to replicate the results of the original paper (Figure 10 in Afyouni and Nichols (2018)) on OBHC data (Supplementary Figure 3), with D-var and S-var converging around 50% of A-var after preprocessing. This supports successful rfMRI preprocessing despite %S-var exceeding the 75% threshold in some volumes, suggesting that this threshold, which was set based on HCP and ABIDE scans, may be too strict for our OBHC setting. Although the mean %A-var metrics were secondarily considered when deciding whether to reject data that were flagged based on the 75% threshold, in future work they could be incorporated into the primary visual and automated QC work to assess rfMRI quality more comprehensively.

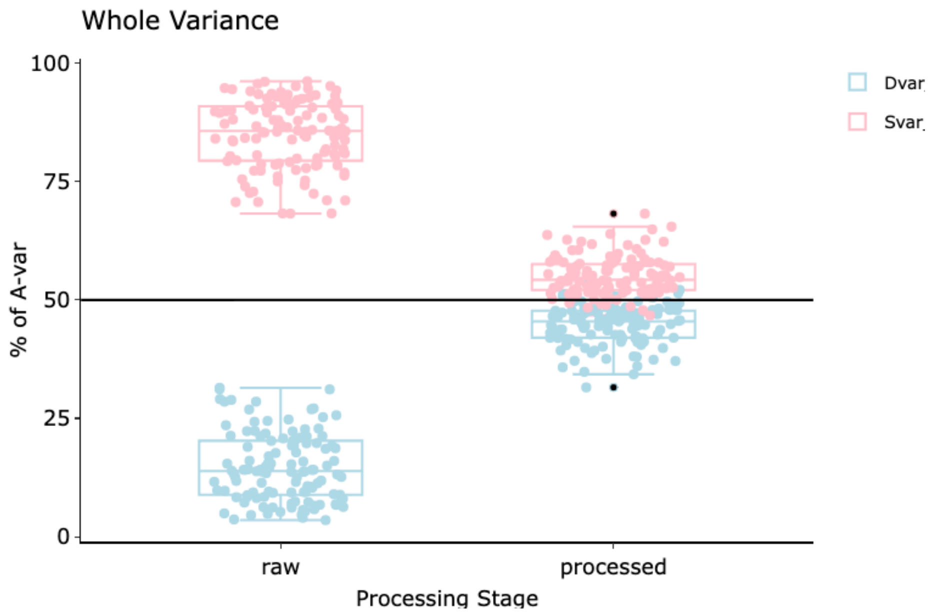

Supplementary Figure 2: Convergence of % of A-var from raw and processed OBHC scans (as done in Figure 10 of Afyouni and Nichols (2018)). In independent and identically-distributed data, D-var and S-var should converge around 50% of A-var.

Supplementary Section 5: Associations with age without ACE-III adjustment

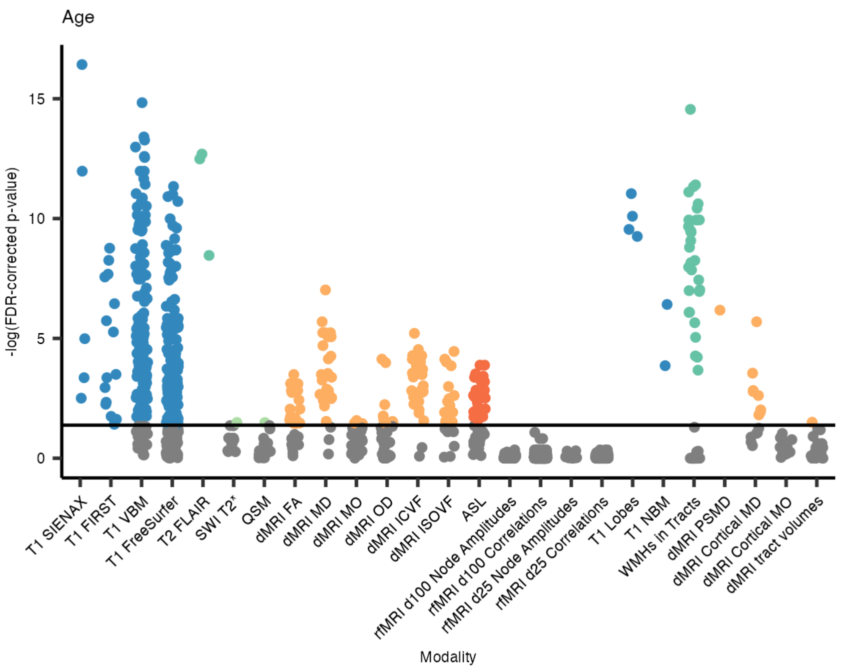

Supplementary Figure 3: FDR-corrected p-values for associations between IDPs and age without covarying for ACE-III total cognitive score. Each dot represents one IDP, grouped by analysis tool/method and colour-coded by scan modality if significant. SIENAX, Structural Image Evaluation using Normalization of Atrophy (cross-sectionally); FIRST, FMRIB's Integrated Registration and Segmentation Tool; VBM, voxel-based morphometry; BIANCA, Brain Intensity AbNormality Classification Algorithm; FA, fractional anisotropy; MD, mean diffusivity; MO, mode of anisotropy; OD, orientation dispersion index; ICVF, intra-cellular volume fraction; ISOVF, isotropic volume fraction; CBF, cerebral blood flow; NBM, nucleus basalis of Meynert; WMH, white matter hyperintensity; PSMD, peak width of skeletonised mean diffusivity.

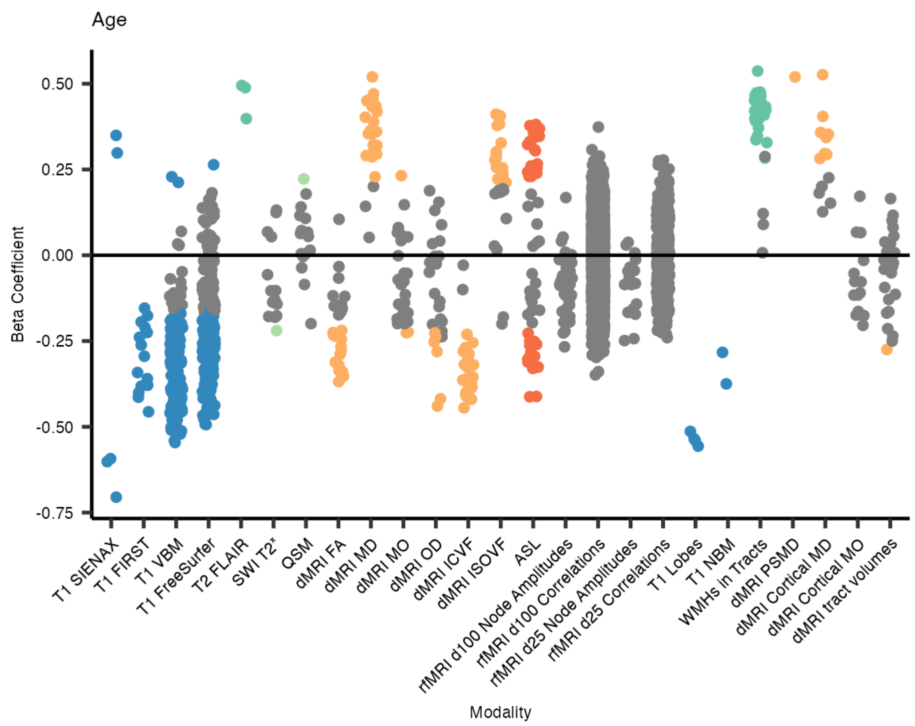

Supplementary Figure 4: Beta coefficients for associations between IDPs and age without covarying for ACE-III total cognitive score. All numeric variables are unit standardised, meaning that 1 standard deviation (SD) increase in an IDP value is associated with a β SD difference in age. Coloured dots indicate associations significant at the 5% FDR level, hierarchical by modality. SIENAX, Structural Image Evaluation using Normalization of Atrophy (cross-sectionally); FIRST, FMRIB's Integrated Registration and Segmentation Tool; VBM, voxel-based morphometry; BIANCA, Brain Intensity AbNormality Classification Algorithm; FA, fractional anisotropy; MD, mean diffusivity; MO, mode of anisotropy; OD, orientation dispersion index; ICVF, intra-cellular volume fraction; ISOVF, isotropic volume fraction; CBF, cerebral blood flow; NBM, nucleus basalis of Meynert; WMH, white matter hyperintensity; PSMD, peak width of skeletonised mean diffusivity.

Supplementary Section 6: Associations with age

Supplementary Table 4: Associations between IDPs and age after adjusting for ACE-III total cognitive score. Corr-pval are after hierarchical FDR correction.

Variable	coef	pval	corr-pval
T1_SIENAX_GM_unnorm_vol_LM	-0.727	3.51E-16	6.93E-14
T1_GM_parcellation_L_VI_Cerebellum_vol_LM	-0.521	2.73E-16	6.93E-14
T2_FLAIR_WMHvol_Superior_corona_radiata_R	0.499	1.51E-13	7.69E-12
T1_GM_parcellation_R_VI_Cerebellum_vol_LM	-0.481	7.34E-14	9.67E-12
T1_GM_parcellation_R_Crus_I_Cerebellum_vol_LM	-0.479	9.80E-14	9.68E-12
T1_GM_parcellation_R_Crus_II_Cerebellum_vol_LM	-0.472	2.38E-13	1.88E-11
T1_GM_parcellation_L_Crus_I_Cerebellum_vol_LM	-0.470	3.20E-13	2.11E-11
T1_GM_parcellation_R_Amygdala_vol_LM	-0.495	2.02E-12	1.14E-10
T1_GM_parcellation_R_Hippocampus_vol_LM	-0.480	4.16E-12	2.05E-10
WMH_pvent	0.468	9.23E-12	2.35E-10
WMH_total	0.457	1.88E-11	3.19E-10
T1_GM_parcellation_L_Crus_II_Cerebellum_vol_LM	-0.441	1.28E-11	5.64E-10
T1_SIENAX_peripheral_GM_unnorm_vol_LM	-0.614	4.45E-11	1.76E-09
T1_GM_parcellation_R_V_Cerebellum_vol_LM	-0.437	7.26E-11	2.61E-09
FS_HippSubfield_rh_volume_Whole.FS_Hippocampal.body.	-0.459	9.57E-11	2.91E-09
T1_GM_parcellation_R_Parahippocampal_Gyr_ant_vol_LM	-0.459	8.91E-11	2.91E-09
T2_FLAIR_WMHvol_Body_of_corpus_callosum	0.430	3.94E-10	5.03E-09
FS_HippSubfield_rh_volume_fimbria.	-0.420	2.57E-10	5.82E-09
T1_GM_parcellation_R_Caudate_vol_LM	-0.486	2.20E-10	5.82E-09
T1_GM_parcellation_L_Amygdala_vol_LM	-0.465	2.30E-10	5.82E-09
T1_GM_parcellation_R_VIIb_Cerebellum_vol_LM	-0.413	2.65E-10	5.82E-09
T1_GM_parcellation_R_VIIIa_Cerebellum_vol_LM	-0.415	2.38E-10	5.82E-09
FS_HippSubfield_rh_volume_GC.ML.DG.body.	-0.456	2.82E-10	5.86E-09
T1_GM_parcellation_L_VIIIa_Cerebellum_vol_LM	-0.411	3.02E-10	5.97E-09
T2_FLAIR_WMHvol_Anterior_corona_radiata_R	0.420	8.97E-10	9.14E-09
T1_GM_parcellation_R_I.IV_Cerebellum_vol_LM	-0.407	5.77E-10	1.08E-08
T2_FLAIR_WMHvol_Splenium_of_corpus_callosum	0.439	1.36E-09	1.16E-08
T2_FLAIR_WMHvol_Anterior_limb_of_internal_capsule_R	0.437	1.74E-09	1.27E-08
T1_GM_parcellation_R_Vent_Striatum_vol_LM	-0.418	7.28E-10	1.31E-08
T1_GM_parcellation_R_Temp_Fusiform_ant_vol_LM	-0.434	8.22E-10	1.41E-08
T1_GMvol_temp	-0.565	8.63E-10	1.42E-08
T2_FLAIR_WMHvol_Posterior_thalamic_radiation_R	0.407	2.69E-09	1.71E-08
T2_FLAIR_WMHvol_Superior_corona_radiata_L	0.407	3.27E-09	1.85E-08
FS_HippSubfield_rh_volume_subiculum.body.	-0.436	1.20E-09	1.89E-08
T1_GM_parcellation_L_VIIb_Cerebellum_vol_LM	-0.399	1.26E-09	1.91E-08
T1_GM_parcellation_Vermis_VIIIa_Cerebellum_vol_LM	-0.404	1.36E-09	2.00E-08
T1_GM_parcellation_R_Planum_Polare_vol_LM	-0.460	1.43E-09	2.01E-08
T2_FLAIR_WMHvol_Superior_fronto.occipital_fasciculus_R	0.428	6.07E-09	3.09E-08

T2_FLAIR_WMHvol_Posterior_corona_radiata_L	0.391	6.76E-09	3.13E-08
FS_HippSubfield_rh_volume_CA4.body.	-0.425	2.42E-09	3.29E-08
T2_FLAIR_WMHvol_Anterior_limb_of_internal_capsule_L	0.410	9.09E-09	3.72E-08
T2_FLAIR_WMHvol_Posterior_thalamic_radiation_L	0.404	9.49E-09	3.72E-08
T2_FLAIR_WMHvol_Genu_of_corpus_callosum	0.384	1.18E-08	4.31E-08
T1_GMvol_par	-0.529	3.55E-09	4.68E-08
T1_GM_parcellation_L_Parahippocampal_Gyr_ant_vol_LM	-0.444	4.03E-09	5.13E-08
FS_HippSubfield_lh_volume_fimbria.	-0.396	5.31E-09	6.17E-08
T1_GM_parcellation_R_Precentral_Gyr_vol_LM	-0.417	5.12E-09	6.17E-08
T1_GM_parcellation_L_Hippocampus_vol_LM	-0.435	5.27E-09	6.17E-08
FS_HippSubfield_rh_volume_Whole.FS_Hippocampus.	-0.420	5.73E-09	6.47E-08
FS_aparc.DKTatlas_lh_thickness_precentral.	-0.388	6.65E-09	7.29E-08
T2_FLAIR_WMHvol_Anterior_corona_radiata_L	0.375	2.32E-08	7.89E-08
T1_GM_parcellation_L_V_Cerebellum_vol_LM	-0.381	1.13E-08	1.21E-07
T2_FLAIR_WMHvol_Superior_longitudinal_fasciculus_R	0.374	4.88E-08	1.56E-07
T2_FLAIR_WMHvol_Posterior_corona_radiata_R	0.364	5.27E-08	1.58E-07
WMH_deep	0.361	5.86E-08	1.66E-07
FS_HippSubfield_rh_volume_presubiculum.head.	-0.397	1.62E-08	1.68E-07
T1_GM_parcellation_Vermis_VIIb_Cerebellum_vol_LM	-0.363	2.46E-08	2.49E-07
T2_FLAIR_WMHvol_External_capsule_L	0.379	9.37E-08	2.51E-07
FS_aparc.DKTatlas_rh_volume_entorhinal.	-0.379	2.68E-08	2.57E-07
T1_GM_parcellation_L_I.IV_Cerebellum_vol_LM	-0.362	2.63E-08	2.57E-07
T1_GMvol_front	-0.487	2.74E-08	2.57E-07
FS_HippSubfield_rh_volume_Whole.FS_Hippocampal.head.	-0.402	3.14E-08	2.89E-07
FS_HippSubfield_lh_volume_GC.ML.DG.body.	-0.418	3.37E-08	3.03E-07
T1_GMvol_occ	-0.469	3.62E-08	3.17E-07
T2_FLAIR_WMHvol_Posterior_limb_of_internal_capsule_L	0.421	1.32E-07	3.37E-07
T2_FLAIR_WMHvol_Superior_fronto.occipital_fasciculus_L	0.367	1.79E-07	4.34E-07
T2_FLAIR_WMHvol_External_capsule_R	0.380	2.21E-07	5.13E-07
T1_GM_parcellation_L_Caudate_vol_LM	-0.431	6.72E-08	5.65E-07
R_hipp_masked	-0.362	6.70E-08	5.65E-07
T1_GM_parcellation_L_X_Cerebellum_vol_LM	-0.391	7.75E-08	6.38E-07
FS_HippSubfield_lh_volume_CA4.body.	-0.403	7.98E-08	6.44E-07
T1_GM_parcellation_R_VIIIb_Cerebellum_vol_LM	-0.353	9.05E-08	7.15E-07
FS_aparc.DKTatlas_rh_thickness_precentral.	-0.354	1.29E-07	9.96E-07
T1_GM_parcellation_R_Postcentral_Gyr_vol_LM	-0.371	1.39E-07	1.06E-06
FS_HippSubfield_lh_volume_subiculum.body.	-0.399	1.46E-07	1.09E-06
T1_GM_parcellation_R_Lingual_Gyr_vol_LM	-0.436	1.62E-07	1.19E-06
FS_HippSubfield_lh_volume_Whole.FS_Hippocampal.body.	-0.395	1.88E-07	1.35E-06
T2_FLAIR_WMHvol_Superior_longitudinal_fasciculus_L	0.354	6.29E-07	1.40E-06
IDP_T1_FIRST_right_thalamus_volume	-0.410	2.51E-07	1.74E-06
FS_HippSubfield_rh_volume_subiculum.head.	-0.369	2.49E-07	1.74E-06

T1_GM_parcellation_L_Precentral_Gyr_vol_LM	-0.372	2.60E-07	1.77E-06
T2_FLAIR_WMHvol_Posterior_limb_of_internal_capsule_R	0.403	8.39E-07	1.78E-06
T1_GM_parcellation_L_Vent_Striatum_vol_LM	-0.358	2.96E-07	1.98E-06
FS_aparc.DKTatlas_rh_thickness_entorhinal.	-0.348	3.39E-07	2.23E-06
IDP_dMRI_ProbtrackX_MD_atr_l	0.502	1.25E-08	2.72E-06
IDP_T1_FIRST_left_accumbens_volume	-0.350	4.52E-07	2.93E-06
FS_aparc.DKTatlas_lh_thickness_entorhinal.	-0.349	4.92E-07	3.14E-06
FS_HippSubfield_rh_volume_CA3.head.	-0.350	5.63E-07	3.53E-06
T1_GM_parcellation_R_Front_Orbital_vol_LM	-0.385	6.44E-07	3.97E-06
IDP_T1_FIRST_right_accumbens_volume	-0.335	7.62E-07	4.63E-06
FS_HippSubfield_rh_volume_CA1.head.	-0.354	8.47E-07	5.07E-06
dMRI_PSMD	0.563	5.09E-08	5.55E-06
T2_FLAIR_WMHvol_Cingulum_cingulate_gyrus_R	0.407	2.88E-06	5.87E-06
FS_HippSubfield_lh_volume_Whole.FS_Hippocampus.	-0.373	1.12E-06	6.63E-06
T1_GM_parcellation_R_Parahippocampal_Gyr_post_vol_LM	-0.332	1.43E-06	8.30E-06
T1_GM_parcellation_R_Precuneous_vol_LM	-0.369	1.54E-06	8.83E-06
T1_GM_parcellation_R_Temp_Pole_vol_LM	-0.343	1.87E-06	1.05E-05
IDP_dMRI_ProbtrackX_MD_atr_r	0.467	1.95E-07	1.42E-05
FS_HippSubfield_rh_volume_GC.ML.DG.head.	-0.336	3.34E-06	1.86E-05
T2_FLAIR_WMHvol_Sagittal_stratum_R	0.347	1.00E-05	1.97E-05
T2_FLAIR_WMHvol_Retrolenticular_part_of_internal_capsule_L	0.361	1.08E-05	1.98E-05
T2_FLAIR_WMHvol_Tapetum_L	0.305	1.06E-05	1.98E-05
FS_aparc.DKTatlas_lh_thickness_superiortemporal.	-0.334	3.77E-06	2.07E-05
T1_GM_parcellation_R_Heschl_Gyr_vol_LM	-0.329	3.90E-06	2.11E-05
IDP_dMRI_ProbtrackX_MD_ifo_r	0.448	5.56E-07	2.42E-05
dMRI_MD_precuneousR	0.516	4.57E-07	2.42E-05
IDP_dMRI_ProbtrackX_MD_str_r	0.445	6.75E-07	2.45E-05
T1_GM_parcellation_R_Intracalcarine_vol_LM	-0.306	4.91E-06	2.62E-05
FS_aparc.DKTatlas_rh_thickness_superiortemporal.	-0.320	4.99E-06	2.63E-05
T1_GM_parcellation_Vermis_VI_Cerebellum_vol_LM	-0.305	5.56E-06	2.89E-05
T1_GM_parcellation_L_Front_Orbital_vol_LM	-0.367	5.64E-06	2.90E-05
IDP_dMRI_ProbtrackX_MD_cgh_r	0.495	1.22E-06	2.95E-05
IDP_dMRI_ProbtrackX_MD_ifo_l	0.441	1.22E-06	2.95E-05
IDP_dMRI_ProbtrackX_MD_unc_r	0.437	1.02E-06	2.95E-05
T1_GM_parcellation_L_Temp_Pole_vol_LM	-0.348	7.12E-06	3.61E-05
T1_GM_parcellation_R_Occ_Fusiform_Gyr_vol_LM	-0.315	7.51E-06	3.75E-05
T1_GM_parcellation_L_Postcentral_Gyr_vol_LM	-0.316	7.90E-06	3.89E-05
T1_GM_parcellation_L_VIIIb_Cerebellum_vol_LM	-0.295	7.97E-06	3.89E-05
T1_GM_parcellation_R_X_Cerebellum_vol_LM	-0.322	8.07E-06	3.89E-05
FS_HippSubfield_rh_volume_CA3.body.	-0.306	8.75E-06	4.17E-05
FS_HippSubfield_lh_volume_presubiculum.head.	-0.332	9.27E-06	4.36E-05
T1_GM_parcellation_L_Planum_Polare_vol_LM	-0.344	9.73E-06	4.52E-05

FS_aparc.DKTatlas_lh_volume_precentral.	-0.324	1.00E-05	4.60E-05
T1_GM_parcellation_L_Temp_Fusiform_ant_vol_LM	-0.315	1.02E-05	4.65E-05
FS_aparc.DKTatlas_lh_thickness_superiorfrontal.	-0.313	1.13E-05	5.01E-05
T1_GM_parcellation_L_Central_Opercular_vol_LM	-0.354	1.12E-05	5.01E-05
IDP_dMRI_ProbtrackX_MD_str_l	0.415	2.39E-06	5.04E-05
IDP_dMRI_ProbtrackX_ICVF_atr_l	-0.423	2.54E-06	5.04E-05
T2_FLAIR_WMHvol_Tapetum_R	0.288	3.04E-05	5.35E-05
FS_aparc.DKTatlas_rh_volume_paraFS_Hippocampal.	-0.302	1.24E-05	5.44E-05
FS_aparc.DKTatlas_lh_thickness_paracentral.	-0.297	1.31E-05	5.69E-05
FS_HippSubfield_lh_volume_Whole.FS_Hippocampal.head.	-0.331	1.41E-05	6.00E-05
T1_GM_parcellation_L_Intracalcarine_vol_LM	-0.299	1.41E-05	6.00E-05
FS_HippSubfield_rh_volume_molecular.layer.HP.body.	-0.306	1.47E-05	6.20E-05
T1_GM_parcellation_R_Temp_Fusiform_post_vol_LM	-0.326	1.66E-05	6.92E-05
FS_HippSubfield_rh_volume_CA4.head.	-0.312	1.92E-05	7.89E-05
T1_GM_parcellation_L_Juxtapositional_Lobule_vol_LM	-0.302	2.06E-05	8.40E-05
FS_aparc.DKTatlas_rh_thickness_superiorfrontal.	-0.298	2.27E-05	9.15E-05
FS_HippSubfield_rh_volume_molecular.layer.HP.head.	-0.302	2.58E-05	1.03E-04
FS_aparc.DKTatlas_rh_thickness_transversetemporal.	-0.285	2.71E-05	1.07E-04
T1_GM_parcellation_Vermis_Crus_II_Cerebellum_vol_LM	-0.278	3.02E-05	1.18E-04
FS_HippSubfield_lh_volume_GC.ML.DG.head.	-0.313	3.29E-05	1.27E-04
T1_GM_parcellation_L_Lingual_Gyr_vol_LM	-0.336	3.34E-05	1.28E-04
T1_NBMvol_L	-0.302	3.62E-05	1.37E-04
FS_aparc.DKTatlas_lh_volume_paracentral.	-0.300	3.72E-05	1.39E-04
FS_aparc.DKTatlas_rh_volume_fusiform.	-0.311	3.74E-05	1.39E-04
T1_GM_parcellation_L_Temp_Occ_Fusiform_vol_LM	-0.304	4.11E-05	1.50E-04
L_hipp_masked	-0.295	4.11E-05	1.50E-04
FS_aparc.DKTatlas_lh_volume_entorhinal.	-0.298	4.40E-05	1.60E-04
FS_HippSubfield_lh_volume_CA4.head.	-0.313	4.54E-05	1.63E-04
FS_aparc.DKTatlas_rh_thickness_supramarginal.	-0.287	4.62E-05	1.64E-04
T1_GM_parcellation_R_Lateral_Occ_Inf_vol_LM	-0.310	4.93E-05	1.74E-04
FS_HippSubfield_lh_volume_CA3.head.	-0.297	5.13E-05	1.78E-04
FS_aparc.DKTatlas_lh_thickness_postcentral.	-0.274	5.14E-05	1.78E-04
IDP_dMRI_ProbtrackX_ICVF_atr_r	-0.402	1.11E-05	1.86E-04
IDP_dMRI_ProbtrackX_ICVF_ifo_r	-0.401	1.03E-05	1.86E-04
FS_aparc.DKTatlas_lh_volume_postcentral.	-0.307	5.46E-05	1.87E-04
FS_HippSubfield_rh_volume_presubiculum.body.	-0.283	5.76E-05	1.93E-04
T1_GM_parcellation_R_Supramarginal_Gyr_ant_vol_LM	-0.287	5.72E-05	1.93E-04
T1_GM_parcellation_R_Juxtapositional_Lobule_vol_LM	-0.276	5.73E-05	1.93E-04
IDP_T1_FIRST_left_thalamus_volume	-0.318	6.04E-05	2.00E-04
T1_GM_parcellation_R_Supramarginal_Gyr_post_vol_LM	-0.297	6.14E-05	2.02E-04
T1_SIENAX_brain.unnorm_vol_LM	-0.534	6.40E-05	2.09E-04
T1_GM_parcellation_L_Paracingulate_Gyr_vol_LM	-0.285	6.65E-05	2.15E-04

IDP_dMRI_ProbtrackX_MD_unc_l	0.390	1.57E-05	2.33E-04
IDP_dMRI_ProbtrackX_ISOVF_atr_l	0.386	1.64E-05	2.33E-04
IDP_dMRI_ProbtrackX_ISOVF_atr_r	0.386	1.71E-05	2.33E-04
IDP_dMRI_ProbtrackX_ICVF_unc_r	-0.383	1.91E-05	2.45E-04
FS_aparc.DKTatlas_rh_thickness_inferiortemporal.	-0.283	7.69E-05	2.47E-04
IDP_dMRI_ProbtrackX_MD_fmi	0.382	2.34E-05	2.83E-04
T1_GM_parcellation_R_Paracingulate_Gyr_vol_LM	-0.280	1.01E-04	3.22E-04
IDP_dMRI_ProbtrackX_MD_cst_l	0.365	3.56E-05	3.30E-04
IDP_dMRI_ProbtrackX_ICVF_cgc_l	-0.401	3.47E-05	3.30E-04
IDP_dMRI_ProbtrackX_ICVF_cgh_r	-0.399	3.32E-05	3.30E-04
IDP_dMRI_ProbtrackX_ICVF_unc_l	-0.375	3.39E-05	3.30E-04
IDP_dMRI_ProbtrackX_OD_atr_l	-0.412	3.63E-05	3.30E-04
IDP_dMRI_ProbtrackX_ISOVF_cgh_r	0.426	2.93E-05	3.30E-04
IDP_dMRI_ProbtrackX_OD_atr_r	-0.422	3.84E-05	3.35E-04
IDP_dMRI_ProbtrackX_ICVF_ifo_l	-0.376	4.47E-05	3.74E-04
T1_GM_parcellation_Vermis_VIIIb_Cerebellum_vol_LM	-0.256	1.24E-04	3.93E-04
FS_aparc.DKTatlas_lh_thickness_supramarginal.	-0.271	1.31E-04	4.11E-04
FS_HippSubfield_lh_volume_CA1.head.	-0.283	1.33E-04	4.15E-04
FS_aparc.DKTatlas_lh_thickness_transversetemporal.	-0.260	1.35E-04	4.16E-04
FS_aparc.DKTatlas_rh_thickness_paracentral.	-0.257	1.46E-04	4.47E-04
FS_aparc.DKTatlas_lh_volume_lateralorbitofrontal.	-0.309	1.48E-04	4.50E-04
FS_aparc.DKTatlas_rh_volume_precentral.	-0.285	1.53E-04	4.62E-04
FS_HippSubfield_lh_volume_subiculum.head.	-0.279	1.67E-04	4.99E-04
FS_HippSubfield_lh_volume_molecular.layer.HP.head.	-0.278	1.74E-04	5.17E-04
IDP_dMRI_ProbtrackX_ISOVF_unc_r	0.361	6.45E-05	5.21E-04
T2_FLAIR_WMHvol_Cingulum_cingulate_gyrus_L	0.307	3.08E-04	5.24E-04
FS_HippSubfield_lh_volume_CA3.body.	-0.269	1.82E-04	5.32E-04
FS_aparc.DKTatlas_lh_volume_fusiform.	-0.295	1.81E-04	5.32E-04
T1_GM_parcellation_R_Temp_Occ_Fusiform_vol_LM	-0.284	1.93E-04	5.60E-04
IDP_dMRI_ProbtrackX_MD_ilf_r	0.367	7.53E-05	5.87E-04
FS_aparc.DKTatlas_lh_thickness_rostralmiddlefrontal.	-0.258	2.04E-04	5.87E-04
T2_FLAIR_WMHvol_Sagittal_stratum_L	0.299	3.71E-04	6.11E-04
FS_aparc.DKTatlas_lh_thickness_caudalmiddlefrontal.	-0.258	2.19E-04	6.26E-04
FS_aparc.DKTatlas_rh_thickness_fusiform.	-0.256	2.57E-04	7.30E-04
T1_NBMvol_R	-0.253	2.97E-04	8.39E-04
T1_GM_parcellation_R_IX_Cerebellum_vol_LM	-0.239	3.23E-04	9.03E-04
T1_GM_parcellation_R_Sup_Temp_Gyr_ant_vol_LM	-0.273	3.25E-04	9.04E-04
IDP_dMRI_ProbtrackX_ICVF_ptr_r	-0.355	1.24E-04	9.29E-04
IDP_dMRI_ProbtrackX_ICVF_str_r	-0.349	1.28E-04	9.29E-04
FS_aparc.DKTatlas_rh_thickness_parsopercularis.	-0.245	3.51E-04	9.68E-04
FS_aparc.DKTatlas_lh_thickness_parstriangularis.	-0.246	3.86E-04	1.06E-03
IDP_dMRI_ProbtrackX_MD_ptr_r	0.347	1.55E-04	1.09E-03

IDP_T1_FIRST_brain_stem.4th_ventricle_volume	-0.267	4.22E-04	1.15E-03
FS_aparc.DKTatlas_lh_thickness_lateralorbitofrontal.	-0.242	4.29E-04	1.16E-03
FS_aparc.DKTatlas_rh_volume_inferiortemporal.	-0.279	4.36E-04	1.17E-03
IDP_dMRI_ProbtrackX_ICVF_str_l	-0.340	1.74E-04	1.18E-03
IDP_dMRI_ProbtrackX_ICVF_cgc_r	-0.355	1.89E-04	1.22E-03
IDP_dMRI_ProbtrackX_ICVF_cgh_l	-0.359	1.96E-04	1.22E-03
IDP_dMRI_ProbtrackX_ICVF_fmi	-0.338	1.93E-04	1.22E-03
IDP_dMRI_ProbtrackX_FA_atr_l	-0.347	2.07E-04	1.25E-03
IDP_dMRI_ProbtrackX_FA_ifo_r	-0.331	2.23E-04	1.32E-03
T1_GM_parcellation_R_Inf_Temp_Gyr_ant_vol_LM	-0.260	5.00E-04	1.33E-03
T1_GM_parcellation_L_Front_Operculum_vol_LM	-0.260	5.03E-04	1.33E-03
FS_aparc.DKTatlas_rh_volume_superiortemporal.	-0.276	5.17E-04	1.36E-03
FS_HippSubfield_rh_volume_CA1.body.	-0.236	5.34E-04	1.40E-03
T1_GM_parcellation_R_Insula_vol_LM	-0.272	5.48E-04	1.42E-03
T1_GM_parcellation_L_Heschl_Gyr_vol_LM	-0.256	5.49E-04	1.42E-03
dMRI_MD_R_hipp	0.378	2.52E-04	1.44E-03
ASL_Right_Thalamus	-0.390	5.82E-05	1.45E-03
ASL_Left_Thalamus	-0.386	3.78E-05	1.45E-03
IDP_dMRI_ProbtrackX_FA_fmi	-0.344	2.72E-04	1.52E-03
IDP_dMRI_ProbtrackX_MD_slf_l	0.342	2.92E-04	1.53E-03
IDP_dMRI_ProbtrackX_MD_slf_r	0.340	3.03E-04	1.53E-03
IDP_dMRI_ProbtrackX_ICVF_mcp	-0.325	3.03E-04	1.53E-03
IDP_dMRI_ProbtrackX_ISOVF_str_r	0.326	2.91E-04	1.53E-03
T1_GM_parcellation_L_Precuneous_vol_LM	-0.280	6.62E-04	1.69E-03
T1_GM_parcellation_R_Front_Operculum_vol_LM	-0.253	6.61E-04	1.69E-03
FS_aparc.DKTatlas_lh_area_transversetemporal.	0.257	6.67E-04	1.69E-03
T1_GM_parcellation_L_Front_Pole_vol_LM	-0.286	6.98E-04	1.75E-03
T1_GM_parcellation_L_Sup_Parietal_Lobule_vol_LM	-0.250	6.95E-04	1.75E-03
FS_aparc.DKTatlas_lh_volume_superiorfrontal.	-0.262	7.18E-04	1.78E-03
FS_aparc.DKTatlas_rh_area_paraFS_Hippocampal.	-0.269	7.36E-04	1.82E-03
ASL_arrival_gm_Left_Temporal_Lobe	0.351	1.54E-04	1.93E-03
ASL_arrival_Left_Putamen	0.344	1.37E-04	1.93E-03
FS_aparc.DKTatlas_rh_volume_isthmuscingulate.	-0.245	7.87E-04	1.93E-03
IDP_T1_FIRST_left_putamen_volume	-0.253	8.52E-04	2.08E-03
IDP_dMRI_ProbtrackX_FA_cgh_r	-0.349	4.48E-04	2.22E-03
IDP_dMRI_ProbtrackX_ISOVF_str_l	0.313	5.40E-04	2.62E-03
T2_FLAIR_WMHvol_Retrolenticular_part_of_internal_capsule_R	0.222	1.73E-03	2.76E-03
T2_FLAIR_WMHvol_Fornix_cres.Stria_terminalis_R	0.360	1.82E-03	2.82E-03
IDP_dMRI_ProbtrackX_MD_cst_r	0.310	6.13E-04	2.90E-03
ASL_arrival_Right_Caudate	0.338	3.19E-04	2.98E-03
ASL_arrival_Left_Caudate	0.329	3.58E-04	2.98E-03

IDP_dMRI_ProbtrackX_FA_ptr_r	-0.305	6.43E-04	2.98E-03
ASL_arrival_Right_Putamen	0.319	4.34E-04	3.10E-03
IDP_dMRI_ProbtrackX_ICVF_ilf_r	-0.315	7.17E-04	3.26E-03
T1_GM_parcellation_L_IX_Cerebellum_vol_LM	-0.212	1.36E-03	3.29E-03
FS_aparc.DKTatlas_rh_thickness_caudalmiddlefrontal.	-0.223	1.38E-03	3.32E-03
FS_aparc.DKTatlas_rh_thickness_parsorbitalis.	-0.221	1.40E-03	3.33E-03
T1_GM_parcellation_L_Parahippocampal_Gyr_post_vol_LM	-0.240	1.40E-03	3.33E-03
FS_HippSubfield_lh_volume_FS_Hippocampal.tail.	-0.243	1.42E-03	3.36E-03
T1_GM_parcellation_R_Front_Medial_vol_LM	-0.226	1.44E-03	3.38E-03
IDP_dMRI_ProbtrackX_FA_ifo_l	-0.304	8.10E-04	3.60E-03
T1_GM_parcellation_R_Central_Opercular_vol_LM	-0.255	1.70E-03	3.98E-03
FS_aparc.DKTatlas_lh_volume_lingual.	-0.223	1.80E-03	4.18E-03
FS_aparc.DKTatlas_lh_thickness_insula.	-0.212	1.83E-03	4.22E-03
T1_GM_parcellation_R_Lateral_Occ_Sup_vol_LM	-0.236	2.05E-03	4.72E-03
FS_aparc.DKTatlas_lh_thickness_parsopercularis.	-0.214	2.09E-03	4.75E-03
FS_aparc.DKTatlas_rh_volume_lateralorbitofrontal.	-0.252	2.09E-03	4.75E-03
IDP_dMRI_ProbtrackX_ICVF_ptr_l	-0.305	1.11E-03	4.84E-03
ASL_gm_Right_Occipital_Lobe	-0.311	8.83E-04	5.52E-03
IDP_dMRI_ProbtrackX_FA_unc_l	-0.293	1.31E-03	5.59E-03
ASL_arrival_gm_LICA	0.320	1.03E-03	5.70E-03
FS_aparc.DKTatlas_lh_area_lingual.	-0.226	2.84E-03	6.39E-03
FS_aparc.DKTatlas_rh_area_rostralanteriorcingulate.	-0.231	2.85E-03	6.39E-03
FS_aparc.DKTatlas_rh_volume_parsorbitalis.	-0.216	2.91E-03	6.48E-03
IDP_dMRI_ProbtrackX_MD_ar_l	0.286	1.66E-03	6.54E-03
IDP_dMRI_ProbtrackX_ICVF_cst_l	-0.286	1.68E-03	6.54E-03
IDP_dMRI_ProbtrackX_ICVF_slf_l	-0.300	1.61E-03	6.54E-03
IDP_dMRI_ProbtrackX_ICVF_slf_r	-0.300	1.57E-03	6.54E-03
dMRI_MD_precuneousL	0.328	1.66E-03	6.54E-03
T1_GM_parcellation_R_Planum_Tempe_vol_LM	-0.234	2.96E-03	6.57E-03
T1_GM_parcellation_Vermis_IX_Cerebellum_vol_LM	-0.198	3.02E-03	6.65E-03
IDP_dMRI_ProbtrackX_ISOVF_cst_l	0.286	1.83E-03	6.98E-03
IDP_T1_FIRST_right_pallidum_volume	-0.201	3.24E-03	7.10E-03
FS_HippSubfield_rh_volume_HATA.	-0.215	3.38E-03	7.37E-03
ASL_gm_Left_Occipital_Lobe	-0.291	1.71E-03	7.63E-03
ASL_arrival_gm_Left_Frontal_Lobe	0.301	1.73E-03	7.63E-03
ASL_arrival_gm_Left_Parietal_Lobe	0.307	1.83E-03	7.63E-03
FS_aparc.DKTatlas_lh_volume_pericalcarine.	-0.198	3.60E-03	7.77E-03
T1_GM_parcellation_L_Mid_Front_Gyr_vol_LM	-0.220	3.62E-03	7.77E-03
T1_GM_parcellation_R_Mid_Temp_Gyr_temporoOcc_part_vol_LM	-0.217	3.61E-03	7.77E-03
FS_aparc.DKTatlas_rh_thickness_insula.	-0.203	3.80E-03	8.11E-03
FS_aparc.DKTatlas_lh_volume_medialorbitofrontal.	-0.230	3.83E-03	8.13E-03

FS_aparc.DKTatlas_rh_volume_paracentral.	-0.209	3.89E-03	8.22E-03
IDP_dMRI_ProbtrackX_MD_ilf_l	0.290	2.23E-03	8.25E-03
IDP_dMRI_ProbtrackX_MD_ptr_l	0.295	2.23E-03	8.25E-03
IDP_dMRI_ProbtrackX_MD_mcp	0.272	2.54E-03	9.24E-03
T1_GM_parcellation_R_Subcallosal_vol_LM	-0.251	4.44E-03	9.32E-03
IDP_dMRI_ProbtrackX_FA_cst_l	-0.266	2.80E-03	9.69E-03
IDP_dMRI_ProbtrackX_MD_cgc_l	0.290	2.78E-03	9.69E-03
dMRI_MD_R_amyg	0.315	2.80E-03	9.69E-03
FS_aparc.DKTatlas_lh_thickness_precuneus.	-0.198	4.71E-03	9.80E-03
T1_GM_parcellation_L_Occ_Pole_vol_LM	-0.203	4.70E-03	9.80E-03
ASL_arrival_gm_70..GM	0.277	2.83E-03	1.01E-02
ASL_arrival_gm_gmmask_pure_cort	0.281	2.65E-03	1.01E-02
IDP_dMRI_ProbtrackX_FA_atr_r	-0.278	2.99E-03	1.02E-02
T1_GM_parcellation_R_Sup_Temp_Gyr_post_vol_LM	-0.213	5.20E-03	1.07E-02
ASL_gm_VBA	-0.277	3.34E-03	1.11E-02
FS_aparc.DKTatlas_lh_area_pericalcarine.	-0.190	5.63E-03	1.16E-02
dMRI_MD_parahippL	0.310	3.46E-03	1.16E-02
FS_HippSubfield_lh_volume_molecular.layer.HP.body.	-0.201	5.70E-03	1.17E-02
FS_aparc.DKTatlas_lh_thickness_medialorbitofrontal.	-0.198	5.75E-03	1.17E-02
ASL_gm_gmmask_pure_cort	-0.277	3.81E-03	1.19E-02
IDP_dMRI_ProbtrackX_OD_mcp	-0.269	3.69E-03	1.22E-02
FS_aparc.DKTatlas_rh_volume_superiorfrontal.	-0.233	6.33E-03	1.28E-02
IDP_dMRI_ProbtrackX_ICVF_fma	-0.268	3.95E-03	1.29E-02
ASL_Left_Caudate	-0.283	4.37E-03	1.29E-02
IDP_dMRI_ProbtrackX_FA_ptr_l	-0.263	4.04E-03	1.30E-02
FS_aparc.DKTatlas_lh_thickness_parsorbitalis.	-0.190	6.53E-03	1.32E-02
IDP_T1_FIRST_right_putamen_volume	-0.194	6.67E-03	1.34E-02
T1_GM_parcellation_L_Front_Medial_vol_LM	-0.194	6.95E-03	1.39E-02
FS_aparc.DKTatlas_rh_volume_parstriangularis.	-0.200	7.45E-03	1.48E-02
FS_aparc.DKTatlas_lh_volume_rostralanteriorcingulate.	-0.227	7.65E-03	1.51E-02
IDP_dMRI_ProbtrackX_ISOVF_ptr_r	0.257	4.87E-03	1.54E-02
FS_aparc.DKTatlas_lh_volume_rostralmiddlefrontal.	-0.209	7.88E-03	1.54E-02
FS_aparc.DKTatlas_rh_area_lingual.	-0.199	7.90E-03	1.54E-02
IDP_dMRI_ProbtrackX_ICVF_ilf_l	-0.266	5.06E-03	1.58E-02
FS_aparc.DKTatlas_lh_volume_inferiortemporal.	-0.210	8.24E-03	1.60E-02
FS_aparc.DKTatlas_rh_thickness_precuneus.	-0.185	8.26E-03	1.60E-02
FS_aparc.DKTatlas_rh_area_pericalcarine.	-0.183	8.30E-03	1.60E-02
ASL_gm_70..GM	-0.263	5.77E-03	1.60E-02
T1_GM_parcellation_L_Sup_Temp_Gyr_post_vol_LM	-0.203	8.48E-03	1.62E-02
T1_GM_parcellation_L_Supracalcarine_vol_LM	-0.193	8.45E-03	1.62E-02
FS_aparc.DKTatlas_rh_area_lateralorbitofrontal.	-0.239	8.67E-03	1.65E-02
IDP_dMRI_ProbtrackX_ISOVF_ifo_r	0.255	5.57E-03	1.71E-02

T1_GM_parcellation_R_Inf_Front_Gyr_pars_triangularis_vol_LM	-0.181	9.18E-03	1.74E-02
IDP_dMRI_ProbtrackX_OD_ilf_r	-0.270	5.87E-03	1.78E-02
IDP_dMRI_ProbtrackX_ICVF_cst_r	-0.260	6.22E-03	1.86E-02
ASL_wm_Left_Cerebral_White_Matter_80..	-0.247	7.75E-03	1.94E-02
ASL_arrival_gm_Right_Occipital_Lobe	0.252	7.38E-03	1.94E-02
IDP_dMRI_ProbtrackX_ISOVF_ilf_r	0.252	6.61E-03	1.95E-02
IDP_dMRI_ProbtrackX_MO_mcp	0.247	6.86E-03	1.97E-02
IDP_dMRI_ProbtrackX_ISOVF_unc_l	0.248	6.78E-03	1.97E-02
FS_aparc.DKTatlas_lh_area_fusiform.	-0.230	1.05E-02	1.97E-02
IDP_dMRI_ProbtrackX_OD_slf_l	-0.259	7.01E-03	1.99E-02
FS_aparc.DKTatlas_lh_area_superiortemporal.	0.240	1.06E-02	1.99E-02
T1_GM_parcellation_L_Pallidum_vol_LM	0.178	1.09E-02	2.03E-02
T1_GM_parcellation_R_Pallidum_vol_LM	0.183	1.10E-02	2.04E-02
ASL_arrival_gm_Left_Occipital_Lobe	0.244	8.67E-03	2.06E-02
T1_GM_parcellation_R_Mid_Temp_Gyr_ant_vol_LM	-0.191	1.13E-02	2.08E-02
IDP_dMRI_ProbtrackX_ISOVF_cst_r	0.244	7.69E-03	2.15E-02
T1_SIENAX_CSF_unnorm_vol_LM	0.220	1.18E-02	2.17E-02
IDP_dMRI_ProbtrackX_MD_cgc_r	0.255	7.99E-03	2.20E-02
IDP_dMRI_ProbtrackX_MD_ml_l	0.241	8.38E-03	2.25E-02
IDP_dMRI_ProbtrackX_ICVF_ar_l	-0.249	8.30E-03	2.25E-02
FS_HippSubfield_rh_volume_FS_Hippocampal.tail.	-0.180	1.24E-02	2.27E-02
T1_GM_parcellation_L_Temp_Fusiform_post_vol_LM	-0.197	1.25E-02	2.28E-02
FS_aparc.DKTatlas_rh_thickness_inferiorparietal.	-0.179	1.26E-02	2.28E-02
IDP_SWI_T2star_right_amygdala.x	-0.220	7.66E-04	2.30E-02
ASL_Right_Caudate	-0.250	1.02E-02	2.31E-02
FS_aparc.DKTatlas_rh_thickness_rostralmiddlefrontal.	-0.173	1.30E-02	2.34E-02
T1_GM_parcellation_L_Mid_Temp_Gyr_ant_vol_LM	-0.205	1.31E-02	2.35E-02
FS_aparc.DKTatlas_rh_volume_inferiorparietal.	-0.208	1.34E-02	2.40E-02
FS_aparc.DKTatlas_rh_volume_precuneus.	-0.196	1.38E-02	2.46E-02
ASL_wm_wmmask_pure_cereb	-0.233	1.18E-02	2.46E-02
ASL_arrival_Right_Thalamus	0.232	1.15E-02	2.46E-02
T1_GM_parcellation_Vermis_X_Cerebellum_vol_LM	-0.164	1.41E-02	2.49E-02
T1_GM_parcellation_R_Front_Pole_vol_LM	-0.213	1.42E-02	2.51E-02
IDP_dMRI_ProbtrackX_OD_ptr_l	-0.257	9.49E-03	2.52E-02
T1_GM_parcellation_L_Sup_Front_Gyr_vol_LM	-0.186	1.44E-02	2.54E-02
FS_aparc.DKTatlas_rh_thickness_paraFS_Hippocampal.	-0.169	1.48E-02	2.59E-02
FS_aparc.DKTatlas_rh_thickness_postcentral.	-0.167	1.51E-02	2.63E-02
T1_GM_parcellation_R_Angular_Gyr_vol_LM	-0.179	1.53E-02	2.64E-02
IDP_dMRI_ProbtrackX_OD_ifo_r	-0.256	1.02E-02	2.67E-02
FS_aparc.DKTatlas_lh_volume_cuneus.	-0.170	1.56E-02	2.70E-02
ASL_gm_Left_Parietal_Lobe	-0.224	1.52E-02	2.71E-02

ASL_wm_Right_Cerebral_White_Matter_80..	-0.224	1.47E-02	2.71E-02
ASL_arrival_gm_Right_Frontal_Lobe	0.233	1.42E-02	2.71E-02
ASL_arrival_gm_Right_Temporal_Lobe	0.231	1.41E-02	2.71E-02
ASL_wm_90..WM	-0.223	1.69E-02	2.73E-02
ASL_arrival_gm_RICA	0.230	1.60E-02	2.73E-02
ASL_arrival_Left_Thalamus	0.218	1.69E-02	2.73E-02
IDP_dMRI_ProbtrackX_FA_slf_r	-0.240	1.06E-02	2.76E-02
FS_HippSubfield_lh_volume_CA1.body.	-0.167	1.67E-02	2.87E-02
IDP_dMRI_ProbtrackX_FA_unc_r	-0.233	1.15E-02	2.94E-02
T1_SIENAX_WM_unnorm_vol_LM	0.267	1.73E-02	2.96E-02
T1_GM_parcellation_L_Cuneal_vol_LM	-0.173	1.80E-02	3.06E-02
FS_aparc.DKTatlas_rh_area_isthmuscingulate.	-0.189	1.84E-02	3.11E-02
T1_GM_parcellation_R_Parietal_Operculum_vol_LM	-0.179	1.85E-02	3.12E-02
IDP_dMRI_ProbtrackX_FA_ilf_r	-0.226	1.32E-02	3.32E-02
IDP_dMRI_ProbtrackX_MD_cgh_l	0.247	1.32E-02	3.32E-02
T1_GM_parcellation_L_Lateral_Occ_Sup_vol_LM	-0.189	1.99E-02	3.35E-02
dMRI_MD_L_amyg	0.260	1.38E-02	3.43E-02
T1_GM_parcellation_L_Inf_Front_Gyr_pars_opercularis_vol_LM	-0.168	2.08E-02	3.48E-02
FS_aparc.DKTatlas_rh_area_caudalanteriorcingulate.	-0.173	2.09E-02	3.48E-02
T1_GM_parcellation_L_Occ_Fusiform_Gyr_vol_LM	-0.179	2.19E-02	3.64E-02
IDP_dMRI_ProbtrackX_FA_ilf_l	-0.221	1.51E-02	3.69E-02
FS_aparc.DKTatlas_lh_thickness_fusiform.	-0.168	2.42E-02	3.99E-02
T1_GM_parcellation_R_Sup_Front_Gyr_vol_LM	-0.168	2.45E-02	4.03E-02
ASL_arrival_gm_Right_Parietal_Lobe	0.219	2.64E-02	4.12E-02

Supplementary Section 7: Associations with age controlling for volume

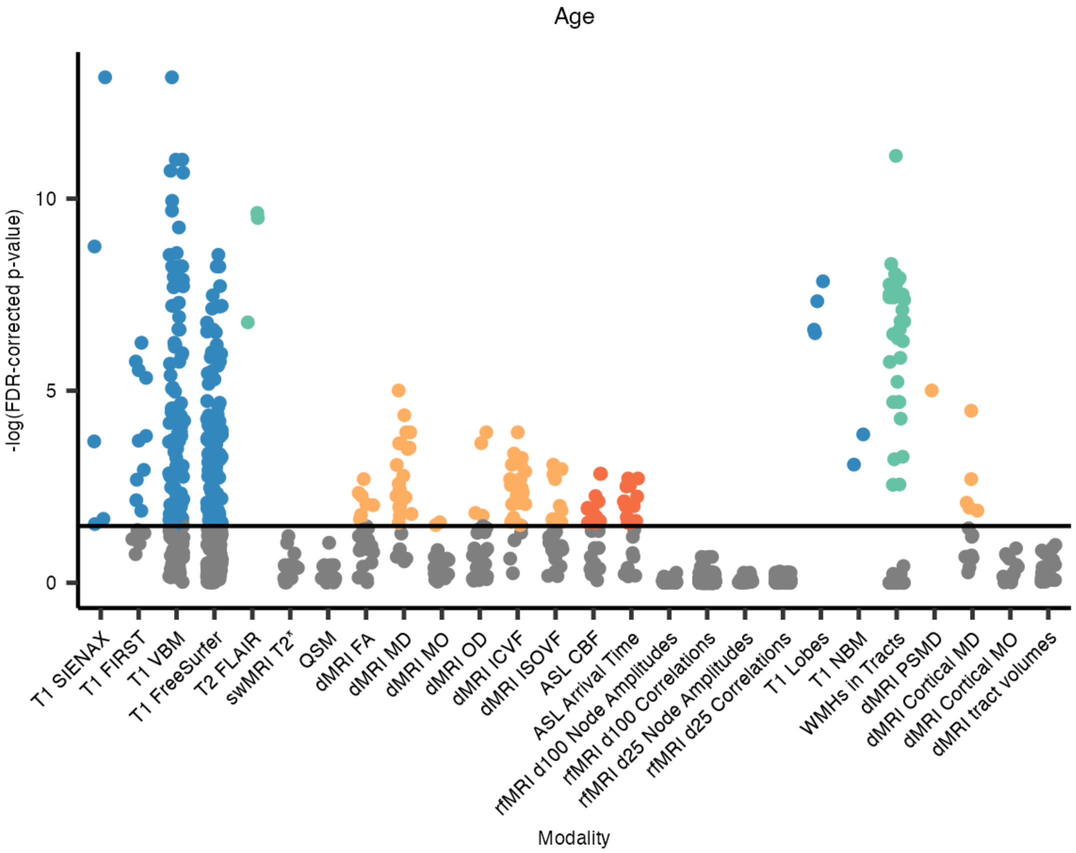

Supplementary Figure 5: FDR-corrected p-values for associations between IDPs and age, controlling for ROI volume with T2 and QSM, and tract volume with tractography-based IDPs (dMRI FA, MD, MO, OD, ICVF, ISOVF). Because the swMRI/QSM modality is no longer significant, the FDR-adjusted threshold (solid line) drops from 0.05(5/6) to 0.05(4/6). SIENAX, Structural Image Evaluation using Normalization of Atrophy (cross-sectionally); FIRST, FMRIB's Integrated Registration and Segmentation Tool; VBM, voxel-based morphometry; BIANCA, Brain Intensity AbNormality Classification Algorithm; FA, fractional anisotropy; MD, mean diffusivity; MO, mode of anisotropy; OD, orientation dispersion index; ICVF, intra-cellular volume fraction; ISOVF, isotropic volume fraction; CBF, cerebral blood flow; NBM, nucleus basalis of Meynert; WMH, white matter hyperintensity; PSMD, peak width of skeletonised mean diffusivity.*

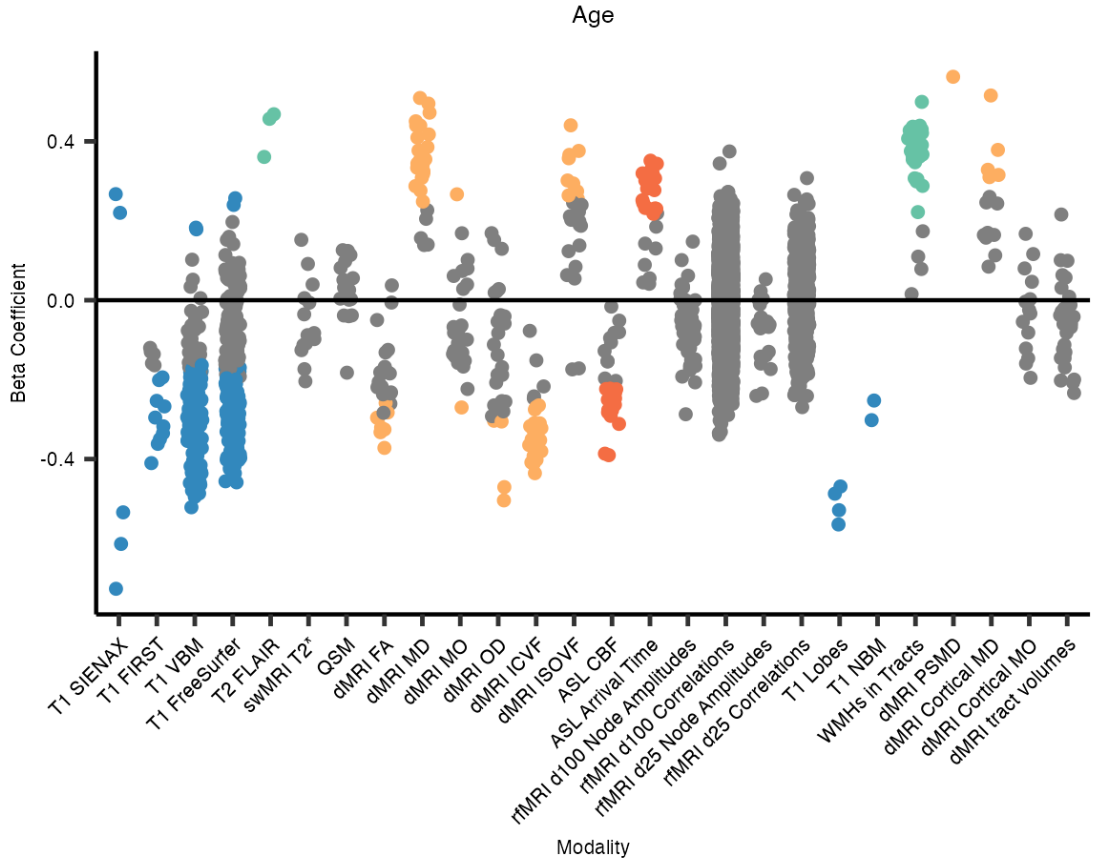

Supplementary Figure 6: Beta coefficients for associations between IDPs and age, controlling for ROI volume with T2 and QSM, and tract volume with tractography-based IDPs (dMRI FA, MD, MO, OD, ICVF, ISOVF). SIENAX, Structural Image Evaluation using Normalization of Atrophy (cross- sectionally); FIRST, FMRIB's Integrated Registration and Segmentation Tool; VBM, voxel- based morphometry; BIANCA, Brain Intensity AbNormality Classification Algorithm; FA, fractional anisotropy; MD, mean diffusivity; MO, mode of anisotropy; OD, orientation dispersion index; ICVF, intra-cellular volume fraction; ISOVF, isotropic volume fraction; CBF, cerebral blood flow; NBM, nucleus basalis of Meynert; WMH, white matter hyperintensity; PSMD, peak width of skeletonised mean diffusivity.*

Supplementary Section 8: Associations with ACE-III

Supplementary Table 5: Associations between IDPs and ACE-III total cognitive score. Corr-pval are after hierarchical FDR correction.

Variable	coef	pval	corr-pval
T1_GMvol_temp	0.648	1.06E-13	4.21E-11
T1_GM_parcellation_L_Mid_Temp_Gyr_post_vol_LM	0.540	2.79E-13	5.51E-11
T1_SIENAX_peripheral_GM_unnorm_vol_LM	0.642	4.62E-12	6.08E-10
FS_aparc.DKTatlas_lh_thickness_middletemporal.	0.429	6.20E-10	6.12E-08
T1_SIENAX_GM_unnorm_vol_LM	0.617	9.72E-10	7.68E-08
T1_GM_parcellation_L_Lateral_Occ_Sup_vol_LM	0.435	3.33E-09	2.19E-07
T1_GMvol_par	0.531	5.46E-09	3.08E-07
T1_GM_parcellation_L_Sup_Temp_Gyr_post_vol_LM	0.398	2.23E-08	1.10E-06
T2_FLAIR_WMHvol_Splenium_of_corpus_callosum	-0.420	2.22E-08	1.13E-06
T1_GM_parcellation_L_Inf_Temp_Gyr_post_vol_LM	0.417	2.64E-08	1.16E-06
FS_aparc.DKTatlas_lh_volume_superiortemporal.	0.415	6.44E-08	2.12E-06
T1_GM_parcellation_L_Temp_Pole_vol_LM	0.411	6.14E-08	2.12E-06
T1_GM_parcellation_R_Mid_Temp_Gyr_post_vol_LM	0.404	5.85E-08	2.12E-06
T1_GM_parcellation_L_Insula_vol_LM	0.414	7.71E-08	2.34E-06
FS_aparc.DKTatlas_lh_volume_middletemporal.	0.402	3.03E-07	8.55E-06
FS_HippSubfield_lh_volume_Whole.FS_Hippocampal.body.	0.394	3.62E-07	9.53E-06
T1_GM_parcellation_L_Mid_Temp_Gyr_ant_vol_LM	0.392	4.36E-07	1.06E-05
T1_GM_parcellation_L_Sup_Parietal_Lobule_vol_LM	0.357	4.56E-07	1.06E-05
IDP_dMRI_ProbtrackX_MD_cgh_r	-0.500	5.15E-08	1.12E-05
FS_HippSubfield_lh_volume_GC.ML.DG.body.	0.392	5.87E-07	1.29E-05
T1_GM_parcellation_L_Sup_Temp_Gyr_ant_vol_LM	0.361	6.64E-07	1.38E-05
FS_HippSubfield_lh_volume_subiculum.body.	0.385	7.96E-07	1.57E-05
FS_aparc.DKTatlas_lh_thickness_fusiform.	0.345	8.83E-07	1.66E-05
T1_GM_parcellation_L_Front_Pole_vol_LM	0.399	9.56E-07	1.72E-05
T1_GMvol_front	0.444	1.18E-06	2.02E-05
dMRI_PSMD	-0.511	1.89E-07	2.06E-05
T1_GM_parcellation_L_Temp_Fusiform_post_vol_LM	0.362	1.40E-06	2.30E-05
FS_HippSubfield_lh_volume_CA4.body.	0.369	2.36E-06	3.61E-05
L_hipp_masked	0.329	2.38E-06	3.61E-05
FS_HippSubfield_lh_volume_subiculum.head.	0.340	3.12E-06	4.17E-05
FS_aparc.DKTatlas_lh_thickness_inferiortemporal.	0.327	3.08E-06	4.17E-05
T1_GM_parcellation_L_Subcallosal_vol_LM	0.393	2.91E-06	4.17E-05
T1_GM_parcellation_L_Precuneous_vol_LM	0.373	3.17E-06	4.17E-05
T1_GM_parcellation_R_Front_Orbital_vol_LM	0.361	5.88E-06	7.49E-05
FS_HippSubfield_lh_volume_Whole.FS_Hippocampus.	0.356	6.23E-06	7.50E-05
FS_aparc.DKTatlas_lh_volume_inferiortemporal.	0.346	6.26E-06	7.50E-05
IDP_dMRI_ProbtrackX_MD_cgh_l	-0.406	1.07E-06	7.80E-05
FS_HippSubfield_lh_volume_presubiculum.head.	0.340	7.19E-06	8.35E-05

FS_aparc.DKTatlas_lh_thickness_inferiorparietal.	0.305	1.27E-05	1.43E-04
FS_aparc.DKTatlas_rh_volume_middletemporal.	0.351	1.56E-05	1.71E-04
IDP_T1_FIRST_left_putamen_volume	0.321	1.77E-05	1.81E-04
FS_aparc.DKTatlas_lh_thickness_superiortemporal.	0.318	1.79E-05	1.81E-04
T1_GM_parcellation_R_Inf_Temp_Gyr_ant_vol_LM	0.317	1.83E-05	1.81E-04
T1_GM_parcellation_L_Parahippocampal_Gyr_ant_vol_LM	0.350	1.70E-05	1.81E-04
FS_aparc.DKTatlas_lh_volume_fusiform.	0.335	2.05E-05	1.98E-04
T1_SIENAX_CSF_unnorm_vol_LM	-0.359	2.25E-05	2.08E-04
T1_GM_parcellation_L_Inf_Temp_Gyr_ant_vol_LM	0.308	2.27E-05	2.08E-04
FS_aparc.DKTatlas_lh_volume_superiorparietal.	0.321	2.32E-05	2.09E-04
T1_GM_parcellation_R_Inf_Temp_Gyr_post_vol_LM	0.331	2.46E-05	2.11E-04
T1_GM_parcellation_L_Amygdala_vol_LM	0.341	2.46E-05	2.11E-04
T1_GMvol_occ	0.379	2.97E-05	2.49E-04
T1_GM_parcellation_L_Parietal_Operculum_vol_LM	0.305	3.95E-05	3.25E-04
T1_GM_parcellation_L_Parahippocampal_Gyr_post_vol_LM	0.304	4.04E-05	3.25E-04
WMH_pvent	-0.331	1.92E-05	3.27E-04
T2_FLAIR_WMHvol_Posterior_thalamic_radiation_L	-0.327	1.36E-05	3.27E-04
FS_HippSubfield_lh_volume_Whole.FS_Hippocampal.head.	0.318	4.55E-05	3.31E-04
T1_GM_parcellation_R_Front_Pole_vol_LM	0.344	4.49E-05	3.31E-04
T1_GM_parcellation_R_Sup_Temp_Gyr_post_vol_LM	0.305	4.29E-05	3.31E-04
T1_GM_parcellation_L_Front_Orbital_vol_LM	0.339	4.49E-05	3.31E-04
T1_GM_parcellation_L_Caudate_vol_LM	0.346	4.61E-05	3.31E-04
T1_GM_parcellation_R_Caudate_vol_LM	0.347	4.46E-05	3.31E-04
T1_GM_parcellation_L_Front_Operculum_vol_LM	0.302	4.87E-05	3.44E-04
FS_HippSubfield_lh_volume_CA4.head.	0.316	5.04E-05	3.50E-04
T1_GM_parcellation_L_Hippocampus_vol_LM	0.326	5.35E-05	3.65E-04
FS_HippSubfield_rh_volume_GC.ML.DG.body.	0.324	5.61E-05	3.69E-04
T1_GM_parcellation_R_Temp_Fusiform_post_vol_LM	0.312	5.58E-05	3.69E-04
IDP_T1_FIRST_left_accumbens_volume	0.292	5.77E-05	3.74E-04
T1_GM_parcellation_R_Temp_Pole_vol_LM	0.300	6.14E-05	3.91E-04
FS_HippSubfield_lh_volume_CA3.body.	0.288	6.99E-05	4.31E-04
FS_aparc.DKTatlas_rh_volume_inferiorparietal.	0.327	6.96E-05	4.31E-04
FS_HippSubfield_rh_volume_subiculum.body.	0.312	7.57E-05	4.60E-04
FS_HippSubfield_lh_volume_GC.ML.DG.head.	0.304	7.71E-05	4.62E-04
IDP_T1_FIRST_right_accumbens_volume	0.281	9.13E-05	5.38E-04
IDP_dMRI_ProbtrackX_ISOVF_cgh_r	-0.413	1.19E-05	6.47E-04
T2_FLAIR_WMHvol_Body_of_corpus_callosum	-0.307	5.37E-05	6.85E-04
WMH_total	-0.304	8.18E-05	8.34E-04
FS_aparc.DKTatlas_lh_volume_entorhinal.	0.283	1.50E-04	8.68E-04
T1_GM_parcellation_R_Insula_vol_LM	0.300	1.52E-04	8.68E-04
T1_GM_parcellation_L_Central_Opercular_vol_LM	0.313	1.74E-04	9.80E-04
FS_HippSubfield_lh_volume_FS_Hippocampal.tail.	0.284	1.88E-04	1.05E-03

IDP_dMRI_ProbtrackX_ICVF_cgh_l	0.371	2.47E-05	1.08E-03
T1_GM_parcellation_R_Mid_Temp_Gyr_ant_vol_LM	0.277	1.97E-04	1.08E-03
FS_HippSubfield_lh_volume_CA1.head.	0.279	2.22E-04	1.20E-03
T1_GM_parcellation_R_Lateral_Occ_Sup_vol_LM	0.281	2.37E-04	1.27E-03
rfMRI_d100_NodeAmplitudes_28	0.427	4.07E-07	1.41E-03
T1_GM_parcellation_R_Sup_Temp_Gyr_ant_vol_LM	0.279	2.90E-04	1.53E-03
FS_aparc.DKTatlas_lh_thickness_entorhinal.	0.267	2.99E-04	1.55E-03
FS_HippSubfield_rh_volume_Whole.FS_Hippocampal.body.	0.290	3.09E-04	1.58E-03
FS_aparc.DKTatlas_lh_volume_supramarginal.	0.279	3.11E-04	1.58E-03
FS_aparc.DKTatlas_rh_volume_posteriorcingulate.	0.258	3.22E-04	1.59E-03
T1_GM_parcellation_L_Planum_Tempe_vol_LM	0.281	3.18E-04	1.59E-03
IDP_dMRI_ProbtrackX_MO_fmi	0.350	4.42E-05	1.61E-03
IDP_T1_FIRST_left_thalamus_volume	0.290	3.54E-04	1.72E-03
T1_GM_parcellation_R_Front_Operculum_vol_LM	0.268	3.56E-04	1.72E-03
T2_FLAIR_WMHvol_Posterior_thalamic_radiation_R	-0.279	2.06E-04	1.75E-03
IDP_dMRI_ProbtrackX_FA_cgh_l	0.351	6.84E-05	1.97E-03
IDP_dMRI_ProbtrackX_ICVF_cgh_r	0.358	8.10E-05	1.97E-03
dMRI_MO_supramargR	0.379	8.12E-05	1.97E-03
FS_HippSubfield_lh_volume_presubiculum.body.	0.254	4.31E-04	2.05E-03
T1_GM_parcellation_L_Paracingulate_Gyr_vol_LM	0.258	4.43E-04	2.09E-03
T2_FLAIR_WMHvol_Anterior_corona_radiata_R	-0.276	2.90E-04	2.11E-03
T1_GM_parcellation_L_Lateral_Occ_Inf_vol_LM	0.265	4.91E-04	2.28E-03
FS_aparc.DKTatlas_lh_volume_parsopercularis.	0.259	5.21E-04	2.37E-03
T1_GM_parcellation_L_Inf_Temp_Gyr_temporoOcc_part_vol_LM	0.254	5.19E-04	2.37E-03
FS_HippSubfield_rh_volume_CA4.body.	0.274	5.38E-04	2.42E-03
FS_aparc.DKTatlas_lh_area_superiortemporal.	0.322	5.57E-04	2.47E-03
FS_HippSubfield_lh_volume_molecular.layer.HP.body.	0.250	5.84E-04	2.56E-03
T1_GM_parcellation_R_Temp_Fusiform_ant_vol_LM	0.271	5.96E-04	2.59E-03
IDP_dMRI_ProbtrackX_FA_cgh_r	0.351	1.26E-04	2.75E-03
T1_GM_parcellation_R_Angular_Gyr_vol_LM	0.249	6.69E-04	2.87E-03
IDP_dMRI_ProbtrackX_ISOVF_cgh_l	-0.319	1.46E-04	2.89E-03
T1_SIENAX_brain.unnorm_vol_LM	0.465	7.44E-04	3.16E-03
T1_GM_parcellation_R_Amygdala_vol_LM	0.276	8.39E-04	3.52E-03
T1_GM_parcellation_L_Supramarginal_Gyr_post_vol_LM	0.261	8.94E-04	3.72E-03
FS_aparc.DKTatlas_lh_thickness_paraFS_Hippocampal.	0.226	9.56E-04	3.93E-03
FS_aparc.DKTatlas_rh_area_middletemporal.	0.297	1.09E-03	4.44E-03
FS_HippSubfield_lh_volume_CA3.head.	0.247	1.16E-03	4.63E-03
FS_HippSubfield_rh_volume_subiculum.head.	0.251	1.15E-03	4.63E-03
T2_FLAIR_WMHvol_Superior_corona_radiata_L	-0.257	7.43E-04	4.74E-03
T1_GM_parcellation_R_Central_Opercular_vol_LM	0.266	1.22E-03	4.81E-03
dMRI_MD_supramargL	-0.361	2.78E-04	5.06E-03

T2_FLAIR_WMHvol_Posterior_corona_radiata_L	-0.247	8.95E-04	5.07E-03
FS_aparc.DKTatlas_rh_thickness_inferiortemporal.	0.238	1.34E-03	5.26E-03
T2_FLAIR_WMHvol_Retrolenticular_part_of_internal_capsule_L	-0.282	1.04E-03	5.28E-03
T1_GM_parcellation_R_Paracingulate_Gyr_vol_LM	0.238	1.38E-03	5.35E-03
T1_GM_parcellation_R_Sup_Parietal_Lobule_vol_LM	0.223	1.43E-03	5.47E-03
T1_GM_parcellation_R_Subcallosal_vol_LM	0.281	1.50E-03	5.69E-03
FS_aparc.DKTatlas_lh_volume_parstriangularis.	0.235	1.56E-03	5.83E-03
FS_aparc.DKTatlas_rh_volume_inferiortemporal.	0.257	1.56E-03	5.83E-03
FS_aparc.DKTatlas_lh_area_inferiortemporal.	0.267	1.61E-03	5.93E-03
rfMRI_d100_NodeAmplitudes_19	0.391	3.44E-06	5.97E-03
FS_aparc.DKTatlas_rh_area_posteriorcingulate.	0.257	1.66E-03	6.07E-03
dMRI_MO_supramargL	0.340	3.63E-04	6.09E-03
T1_GM_parcellation_R_Planum_Polare_vol_LM	0.268	1.71E-03	6.21E-03
T2_FLAIR_WMHvol_Superior_longitudinal_fasciculus_L	-0.242	1.53E-03	6.50E-03
T2_FLAIR_WMHvol_Tapetum_L	-0.230	1.52E-03	6.50E-03
FS_aparc.DKTatlas_lh_volume_posteriorcingulate.	0.236	1.81E-03	6.51E-03
T2_FLAIR_WMHvol_Superior_corona_radiata_R	-0.254	1.82E-03	7.13E-03
FS_HippSubfield_rh_volume_Whole.FS_Hippocampus.	0.249	2.00E-03	7.13E-03
FS_aparc.DKTatlas_rh_volume_medialorbitofrontal.	0.236	2.04E-03	7.21E-03
T1_GM_parcellation_R_Supramarginal_Gyr_post_vol_LM	0.236	2.18E-03	7.62E-03
rfMRI_d100_NodeAmplitudes_21	0.385	8.33E-06	8.11E-03
rfMRI_d100_partialcorr_827	0.378	9.36E-06	8.11E-03
T2_FLAIR_WMHvol_Cingulum_cingulate_gyrus_L	-0.266	2.31E-03	8.41E-03
FS_aparc.DKTatlas_lh_volume_insula.	0.238	2.54E-03	8.79E-03
T2_FLAIR_WMHvol_Retrolenticular_part_of_internal_capsule_R	-0.216	2.64E-03	8.96E-03
FS_aparc.DKTatlas_rh_thickness_entorhinal.	0.223	2.64E-03	8.98E-03
FS_aparc.DKTatlas_rh_thickness_middletemporal.	0.216	2.64E-03	8.98E-03
FS_aparc.DKTatlas_lh_volume_medialorbitofrontal.	0.242	2.69E-03	9.08E-03
rfMRI_d100_NodeAmplitudes_44	0.387	1.58E-05	9.14E-03
rfMRI_d25_NodeAmplitudes_6	0.369	1.58E-05	9.14E-03
FS_aparc.DKTatlas_rh_area_inferiorparietal.	0.266	2.84E-03	9.50E-03
IDP_T1_FIRST_right_putamen_volume	0.215	2.86E-03	9.51E-03
FS_aparc.DKTatlas_lh_area_superiorparietal.	0.235	3.08E-03	1.01E-02
rfMRI_d25_NodeAmplitudes_13	0.371	2.05E-05	1.02E-02
T1_GM_parcellation_L_Sup_Front_Gyr_vol_LM	0.225	3.14E-03	1.02E-02
T1_GM_parcellation_R_Inf_Front_Gyr_pars_opercularis_vol_LM	0.213	3.15E-03	1.02E-02
T2_FLAIR_WMHvol_Posterior_corona_radiata_R	-0.216	3.29E-03	1.05E-02
T2_FLAIR_WMHvol_Genu_of_corpus_callosum	-0.218	3.53E-03	1.06E-02
T1_GM_parcellation_R_Inf_Temp_Gyr_temporoOcc_part_vol_LM	0.233	3.44E-03	1.10E-02
T1_GM_parcellation_R_Precuneous_vol_LM	0.240	3.56E-03	1.13E-02

dMRI_tractvol_fma	0.367	7.49E-04	1.17E-02
FS_aparc.DKTatlas_lh_thickness_supramarginal.	0.213	3.74E-03	1.17E-02
FS_aparc.DKTatlas_rh_volume_superiortemporal.	0.238	3.72E-03	1.17E-02
T1_GM_parcellation_L_Planum_Polare_vol_LM	0.238	3.76E-03	1.17E-02
T1_GM_parcellation_R_Parahippocampal_Gyr_ant_vol_LM	0.236	3.85E-03	1.19E-02
IDP_dMRI_ProbtrackX_ICVF_cgc_l	0.315	8.19E-04	1.19E-02
T1_GM_parcellation_R_Mid_Temp_Gyr_temporoOcc_part_vol_LM	0.218	4.00E-03	1.23E-02
T1_GM_parcellation_R_Lingual_Gyr_vol_LM	0.260	4.26E-03	1.30E-02
rfMRI_d100_NodeAmplitudes_15	0.359	3.45E-05	1.30E-02
rfMRI_d100_NodeAmplitudes_24	0.355	3.41E-05	1.30E-02
rfMRI_d100_NodeAmplitudes_45	0.360	3.75E-05	1.30E-02
T2_FLAIR_WMHvol_Anterior_corona_radiata_L	-0.211	4.61E-03	1.31E-02
IDP_dMRI_ProbtrackX_ISOVF_ptr_l	-0.287	9.73E-04	1.33E-02
IDP_dMRI_ProbtrackX_MD_ptr_l	-0.293	1.04E-03	1.33E-02
IDP_dMRI_ProbtrackX_OD_ar_l	0.273	1.14E-03	1.34E-02
dMRI_tractvol_cst_l	-0.351	1.17E-03	1.34E-02
R_hipp_masked	0.209	4.48E-03	1.35E-02
FS_aparc.DKTatlas_rh_thickness_inferiorparietal.	0.204	4.70E-03	1.40E-02
FS_aparc.DKTatlas_rh_thickness_superiortemporal.	0.211	4.75E-03	1.40E-02
T1_GM_parcellation_R_Mid_Front_Gyr_vol_LM	0.229	4.79E-03	1.40E-02
T1_GM_parcellation_L_Cuneal_vol_LM	0.207	4.77E-03	1.40E-02
FS_aparc.DKTatlas_lh_volume_precuneus.	0.217	5.08E-03	1.48E-02
IDP_dMRI_ProbtrackX_MD_ilf_l	-0.283	1.37E-03	1.49E-02
dMRI_MD_parahippR	-0.314	1.46E-03	1.52E-02
T1_GM_parcellation_L_Angular_Gyr_vol_LM	0.204	5.44E-03	1.57E-02
rfMRI_d100_partialcorr_824	0.343	5.03E-05	1.58E-02
T1_GM_parcellation_L_Front_Medial_vol_LM	0.201	5.56E-03	1.59E-02
IDP_dMRI_ProbtrackX_OD_str_r	0.267	1.65E-03	1.61E-02
dMRI_MD_precuneousR	-0.346	1.70E-03	1.61E-02
T1_NBMvol_L	0.212	5.81E-03	1.65E-02
rfMRI_d100_NodeAmplitudes_29	0.342	5.76E-05	1.66E-02
FS_aparc.DKTatlas_lh_volume_paraFS_Hippocampal.	0.192	5.97E-03	1.68E-02
T1_GM_parcellation_L_Occ_Fusiform_Gyr_vol_LM	0.215	6.01E-03	1.68E-02
dMRI_MD_L_hipp	-0.301	1.88E-03	1.70E-02
T1_GM_parcellation_R_Hippocampus_vol_LM	0.224	6.20E-03	1.73E-02
FS_aparc.DKTatlas_lh_area_middletemporal.	0.245	6.27E-03	1.73E-02
FS_aparc.DKTatlas_rh_thickness_paraFS_Hippocampal.	0.189	6.78E-03	1.86E-02
FS_aparc.DKTatlas_lh_thickness_medialorbitofrontal.	0.197	6.88E-03	1.87E-02
FS_HippSubfield_rh_volume_CA1.head.	0.209	7.26E-03	1.93E-02
FS_HippSubfield_rh_volume_fimbria.	0.205	7.33E-03	1.93E-02
FS_aparc.DKTatlas_lh_area_supramarginal.	0.243	7.22E-03	1.93E-02

T1_GM_parcellation_L_Lingual_Gyr_vol_LM	0.229	7.26E-03	1.93E-02
T1_GM_parcellation_L_Temp_Fusiform_ant_vol_LM	0.204	7.30E-03	1.93E-02
FS_HippSubfield_lh_volume_fimbria.	0.205	7.39E-03	1.93E-02
FS_HippSubfield_rh_volume_Whole.FS_Hippocampal.head.	0.215	7.75E-03	2.01E-02
FS_aparc.DKTatlas_lh_volume_isthmuscingulate.	0.206	7.84E-03	2.03E-02
T1_GM_parcellation_R_Postcentral_Gyr_vol_LM	0.205	7.99E-03	2.05E-02
FS_aparc.DKTatlas_rh_thickness_fusiform.	0.192	8.27E-03	2.11E-02
T1_GM_parcellation_L_Supramarginal_Gyr_ant_vol_LM	0.194	8.75E-03	2.22E-02
rfMRI_d100_NodeAmplitudes_31	0.339	9.02E-05	2.23E-02
rfMRI_d100_fullcorr_824	0.335	8.83E-05	2.23E-02
IDP_dMRI_ProbtrackX_ICVF_slf_l	0.269	2.70E-03	2.26E-02
IDP_dMRI_ProbtrackX_OD_str_l	0.266	2.66E-03	2.26E-02
FS_HippSubfield_lh_volume_CA1.body.	0.184	9.21E-03	2.32E-02
IDP_dMRI_ProbtrackX_ICVF_ilf_l	0.262	2.92E-03	2.36E-02
T2_FLAIR_WMHvol_Tapetum_R	-0.190	9.16E-03	2.46E-02
IDP_dMRI_ProbtrackX_OD_slf_l	0.262	3.22E-03	2.50E-02
FS_HippSubfield_rh_volume_CA3.body.	0.188	1.04E-02	2.61E-02
T2_FLAIR_WMHvol_Fornix_cres.Stria_terminalis_L	-0.379	1.07E-02	2.62E-02
T2_FLAIR_WMHvol_Superior_fronto.occipital_fasciculus_R	-0.211	1.08E-02	2.62E-02
IDP_dMRI_ProbtrackX_MO_cgc_r	0.259	3.52E-03	2.64E-02
IDP_dMRI_ProbtrackX_MO_cgc_l	0.249	3.98E-03	2.72E-02
IDP_dMRI_ProbtrackX_ICVF_unc_l	0.257	4.02E-03	2.72E-02
dMRI_MD_parahippL	-0.290	4.09E-03	2.72E-02
dMRI_MD_R_amyg	-0.290	4.11E-03	2.72E-02
rfMRI_d100_partialcorr_639	-0.336	1.20E-04	2.77E-02
T1_GM_parcellation_L_Inf_Front_Gyr_pars_opercularis_vol_LM	0.186	1.13E-02	2.81E-02
T2_FLAIR_WMHvol_Superior_longitudinal_fasciculus_R	-0.191	1.23E-02	2.84E-02
IDP_dMRI_ProbtrackX_MD_slf_l	-0.258	4.61E-03	2.96E-02
FS_aparc.DKTatlas_lh_area_parsopercularis.	0.199	1.24E-02	3.05E-02
IDP_dMRI_ProbtrackX_MD_unc_l	-0.253	5.10E-03	3.09E-02
IDP_dMRI_ProbtrackX_OD_slf_r	0.253	5.08E-03	3.09E-02
dMRI_MD_supramargR	-0.274	5.25E-03	3.09E-02
dMRI_MD_precuneousL	-0.281	5.42E-03	3.11E-02

Supplementary Section 9: Associations with cognition controlling for volume

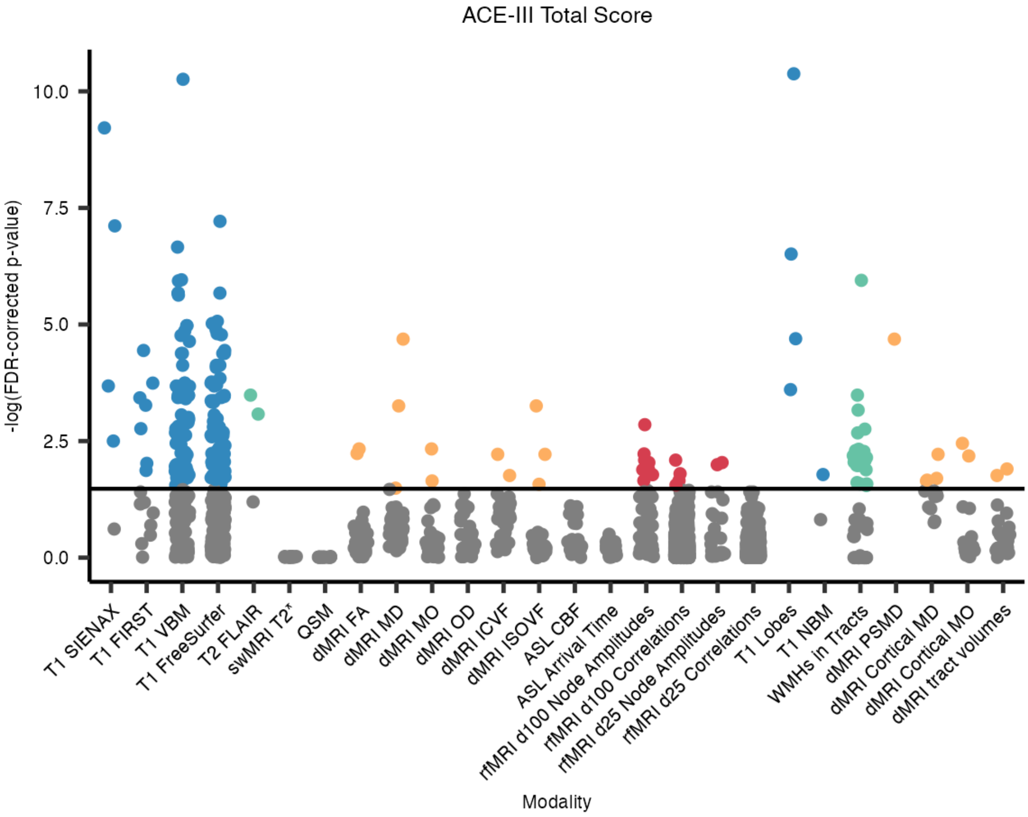

Supplementary Figure 7: FDR-corrected p-values for associations between IDPs and cognition, controlling for ROI volume with T2 and QSM, and tract volume with tractography-based IDPs (dMRI FA, MD, MO, OD, ICVF, ISOVF). SIENAX, Structural Image Evaluation using Normalization of Atrophy (cross-sectionally); FIRST, FMRIB's Integrated Registration and Segmentation Tool; VBM, voxel-based morphometry; BIANCA, Brain Intensity AbNormality Classification Algorithm; FA, fractional anisotropy; MD, mean diffusivity; MO, mode of anisotropy; OD, orientation dispersion index; ICVF, intra-cellular volume fraction; ISOVF, isotropic volume fraction; CBF, cerebral blood flow; NBM, nucleus basalis of Meynert; WMH, white matter hyperintensity; PSMD, peak width of skeletonised mean diffusivity.*

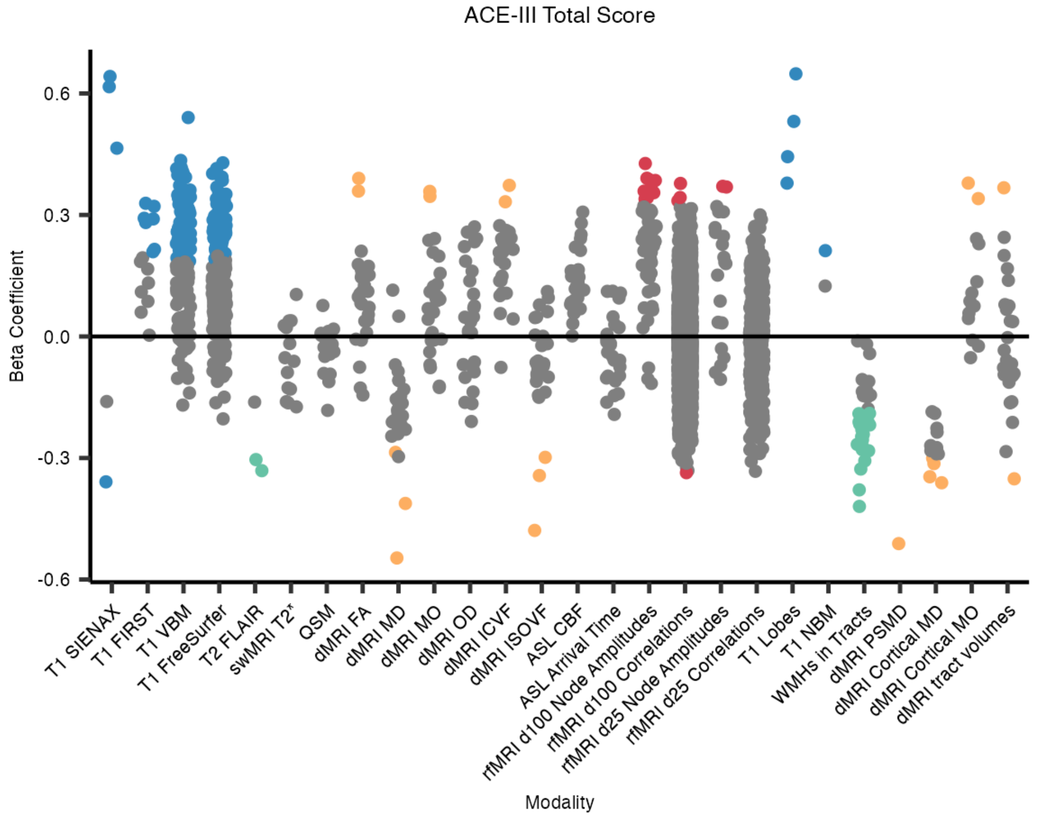

Supplementary Figure 8: Beta coefficients for associations between IDPs and cognition, controlling for ROI volume with T2 and QSM, and tract volume with tractography-based IDPs (dMRI FA, MD, MO, OD, ICVF, ISOVF). SIENAX, Structural Image Evaluation using Normalization of Atrophy (cross- sectionally); FIRST, FMRIB's Integrated Registration and Segmentation Tool; VBM, voxel- based morphometry; BIANCA, Brain Intensity AbNormality Classification Algorithm; FA, fractional anisotropy; MD, mean diffusivity; MO, mode of anisotropy; OD, orientation dispersion index; ICVF, intra-cellular volume fraction; ISOVF, isotropic volume fraction; CBF, cerebral blood flow; NBM, nucleus basalis of Meynert; WMH, white matter hyperintensity; PSMD, peak width of skeletonised mean diffusivity.*

Supplementary Section 10: Associations with diagnosis

Supplementary Table 6: Associations between IDPs and diagnostic groups. Corr-pval are after hierarchical FDR correction.

Variable	coef	OR	pval	corr-pval
T1_GMvol_temp	-1.917	0.147	1.91E-12	7.56E-10
T1_SIENAX_peripheral_GM_unnorm_vol_LM	-1.856	0.156	1.50E-11	2.96E-09
T1_GM_parcellation_L_Front_Pole_vol_LM	-1.326	0.265	1.82E-09	2.40E-07
T1_GM_parcellation_L_Parahippocampal_Gyr_ant_vol_LM	-1.221	0.295	3.94E-09	3.89E-07
T1_SIENAX_GM_unnorm_vol_LM	-1.588	0.204	7.01E-09	4.81E-07
T1_GM_parcellation_R_Mid_Temp_Gyr_post_vol_LM	-1.083	0.339	7.31E-09	4.81E-07
T1_GMvol_front	-1.343	0.261	1.51E-08	7.48E-07
T1_GMvol_par	-1.297	0.273	1.41E-08	7.48E-07
FS_HippSubfield_lh_volume_GC.ML.DG.body.	-1.067	0.344	1.88E-08	8.24E-07
FS_HippSubfield_lh_volume_Whole.FS_Hippocampal.body.	-1.067	0.344	2.59E-08	1.02E-06
FS_HippSubfield_lh_volume_presubiculum.head.	-1.014	0.363	3.03E-08	1.09E-06
FS_HippSubfield_lh_volume_CA4.body.	-1.017	0.362	4.13E-08	1.36E-06
T1_GM_parcellation_L_Temp_Pole_vol_LM	-1.018	0.361	5.17E-08	1.57E-06
FS_HippSubfield_lh_volume_Whole.FS_Hippocampus.	-1.011	0.364	8.32E-08	2.29E-06
T1_GM_parcellation_R_Front_Orbital_vol_LM	-1.074	0.342	8.68E-08	2.29E-06
FS_aparc.DKTatlas_lh_thickness_middletemporal.	-0.900	0.407	9.41E-08	2.32E-06
FS_HippSubfield_lh_volume_Whole.FS_Hippocampal.head.	-0.960	0.383	1.85E-07	4.30E-06
L_hipp_masked	-0.901	0.406	2.14E-07	4.70E-06
T1_GM_parcellation_L_Insula_vol_LM	-0.989	0.372	2.53E-07	5.27E-06
FS_HippSubfield_lh_volume_subiculum.body.	-0.960	0.383	2.68E-07	5.30E-06
T2_FLAIR_WMHvol_Splenium_of_corpus_callosum	0.964	2.621	1.12E-07	5.71E-06
FS_HippSubfield_lh_volume_CA4.head.	-0.920	0.398	3.35E-07	6.01E-06
T1_GM_parcellation_R_Temp_Pole_vol_LM	-0.961	0.383	3.20E-07	6.01E-06
T1_GM_parcellation_R_Sup_Temp_Gyr_post_vol_LM	-0.880	0.415	4.06E-07	6.98E-06
T1_GM_parcellation_L_Hippocampus_vol_LM	-0.972	0.379	4.63E-07	7.62E-06
FS_HippSubfield_rh_volume_GC.ML.DG.body.	-0.932	0.394	4.85E-07	7.66E-06
FS_HippSubfield_lh_volume_GC.ML.DG.head.	-0.891	0.410	6.07E-07	9.23E-06
FS_HippSubfield_lh_volume_CA3.head.	-0.863	0.422	7.56E-07	1.11E-05
T1_GM_parcellation_R_Paracingulate_Gyr_vol_LM	-0.864	0.421	9.97E-07	1.36E-05
T1_GM_parcellation_R_Amygdala_vol_LM	-0.971	0.379	9.63E-07	1.36E-05
T1_GM_parcellation_L_Sup_Temp_Gyr_ant_vol_LM	-0.839	0.432	1.23E-06	1.57E-05
T1_GM_parcellation_L_Mid_Temp_Gyr_post_vol_LM	-0.880	0.415	1.22E-06	1.57E-05
T1_GM_parcellation_R_Front_Pole_vol_LM	-0.996	0.369	1.53E-06	1.89E-05
FS_HippSubfield_rh_volume_Whole.FS_Hippocampal.body.	-0.884	0.413	1.60E-06	1.92E-05
T1_GM_parcellation_R_Parahippocampal_Gyr_ant_vol_LM	-0.928	0.395	1.78E-06	2.07E-05

FS_HippSubfield_rh_volume_subiculum.body.	-0.871	0.418	1.99E-06	2.24E-05
FS_HippSubfield_lh_volume_CA3.body.	-0.784	0.457	2.26E-06	2.48E-05
FS_HippSubfield_lh_volume_CA1.head.	-0.825	0.438	2.41E-06	2.57E-05
T1_GM_parcellation_R_Insula_vol_LM	-0.923	0.397	2.52E-06	2.62E-05
T1_GM_parcellation_L_Amygdala_vol_LM	-0.913	0.401	2.73E-06	2.77E-05
FS_HippSubfield_lh_volume_subiculum.head.	-0.773	0.462	3.43E-06	3.39E-05
FS_HippSubfield_rh_volume_CA4.body.	-0.813	0.443	3.79E-06	3.65E-05
T1_GM_parcellation_L_Lateral_Occ_Sup_vol_LM	-0.773	0.462	4.09E-06	3.85E-05
T1_GM_parcellation_R_Inf_Temp_Gyr_post_vol_LM	-0.819	0.441	4.55E-06	4.18E-05
T1_GM_parcellation_L_Precuneous_vol_LM	-0.846	0.429	4.83E-06	4.34E-05
T1_GM_parcellation_L_Mid_Temp_Gyr_ant_vol_LM	-0.783	0.457	5.11E-06	4.48E-05
T1_GM_parcellation_L_Temp_Fusiform_ant_vol_LM	-0.790	0.454	5.64E-06	4.85E-05
T1_GM_parcellation_L_Temp_Fusiform_post_vol_LM	-0.796	0.451	6.26E-06	5.26E-05
FS_HippSubfield_rh_volume_Whole.FS_Hippocampus.	-0.821	0.440	7.23E-06	5.68E-05
FS_aparc.DKTatlas_lh_thickness_inferiortemporal.	-0.722	0.486	7.11E-06	5.68E-05
T1_GM_parcellation_L_Inf_Temp_Gyr_post_vol_LM	-0.822	0.440	7.34E-06	5.68E-05
T1_GM_parcellation_L_Paracingulate_Gyr_vol_LM	-0.773	0.462	7.15E-06	5.68E-05
T1_GM_parcellation_R_Precuneous_vol_LM	-0.842	0.431	7.49E-06	5.69E-05
FS_HippSubfield_lh_volume_fimbria.	-0.778	0.459	8.03E-06	5.99E-05
T1_GM_parcellation_L_Inf_Temp_Gyr_ant_vol_LM	-0.734	0.480	8.89E-06	6.50E-05
FS_HippSubfield_rh_volume_CA1.head.	-0.781	0.458	9.33E-06	6.58E-05
T1_GM_parcellation_R_Inf_Temp_Gyr_temporoOcc_part_vol_LM	-0.748	0.473	9.31E-06	6.58E-05
R_hipp_masked	-0.720	0.487	1.27E-05	8.83E-05
FS_aparc.DKTatlas_rh_thickness_entorhinal.	-0.700	0.496	1.59E-05	1.08E-04
T1_GM_parcellation_L_Sup_Parietal_Lobule_vol_LM	-0.684	0.504	1.74E-05	1.16E-04
T1_GM_parcellation_R_Inf_Temp_Gyr_ant_vol_LM	-0.707	0.493	2.04E-05	1.34E-04
FS_aparc.DKTatlas_lh_volume_middletemporal.	-0.799	0.450	2.21E-05	1.43E-04
FS_HippSubfield_rh_volume_Whole.FS_Hippocampal.head.	-0.754	0.470	2.41E-05	1.53E-04
T1_GM_parcellation_R_Temp_Fusiform_ant_vol_LM	-0.778	0.459	2.60E-05	1.61E-04
T1_GM_parcellation_L_Front_Operculum_vol_LM	-0.698	0.497	2.56E-05	1.61E-04
T1_SIENAX_CSF_unnorm_vol_LM	0.793	2.211	2.69E-05	1.64E-04
WMH_pvent	0.769	2.157	6.76E-06	1.72E-04
FS_aparc.DKTatlas_lh_volume_superiortemporal.	-0.750	0.472	2.88E-05	1.72E-04
FS_HippSubfield_rh_volume_fimbria.	-0.714	0.490	3.13E-05	1.79E-04
FS_aparc.DKTatlas_lh_thickness_entorhinal.	-0.679	0.507	3.09E-05	1.79E-04
T1_GM_parcellation_R_Hippocampus_vol_LM	-0.765	0.466	3.13E-05	1.79E-04
FS_HippSubfield_rh_volume_CA3.body.	-0.660	0.517	3.52E-05	1.98E-04
FS_aparc.DKTatlas_lh_thickness_superiortemporal.	-0.683	0.505	3.80E-05	2.11E-04
T2_FLAIR_WMHvol_Body_of_corpus_callosum	0.742	2.100	1.30E-05	2.22E-04
FS_aparc.DKTatlas_lh_volume_inferiortemporal.	-0.703	0.495	4.22E-05	2.32E-04

T1_GM_parcellation_R_Mid_Temp_Gyr_ant_vol_LM	-0.685	0.504	4.37E-05	2.36E-04
FS_HippSubfield_rh_volume_CA3.head.	-0.688	0.503	4.82E-05	2.57E-04
T1_GM_parcellation_L_Front_Orbital_vol_LM	-0.752	0.471	4.89E-05	2.58E-04
rfMRI_d100_NodeAmplitudes_28	-1.250	0.286	9.24E-08	3.20E-04
T1_GM_parcellation_R_Central_Opercular_vol_LM	-0.741	0.476	6.33E-05	3.29E-04
FS_aparc.DKTatlas_lh_thickness_fusiform.	-0.632	0.532	7.04E-05	3.56E-04
FS_aparc.DKTatlas_rh_volume_middletemporal.	-0.719	0.487	7.04E-05	3.56E-04
T1_GM_parcellation_R_Caudate_vol_LM	-0.754	0.471	7.66E-05	3.83E-04
FS_HippSubfield_rh_volume_GC.ML.DG.head.	-0.657	0.518	9.77E-05	4.83E-04
FS_aparc.DKTatlas_rh_thickness_superiortemporal.	-0.648	0.523	1.01E-04	4.86E-04
T1_GM_parcellation_L_Sup_Front_Gyr_vol_LM	-0.650	0.522	9.99E-05	4.86E-04
T1_GM_parcellation_L_Sup_Temp_Gyr_post_vol_LM	-0.605	0.546	1.10E-04	5.05E-04
T1_GM_parcellation_R_Subcallosal_vol_LM	-0.776	0.460	1.09E-04	5.05E-04
T1_GM_parcellation_L_Central_Opercular_vol_LM	-0.738	0.478	1.09E-04	5.05E-04
T1_GM_parcellation_R_Pallidum_vol_LM	0.620	1.860	1.07E-04	5.05E-04
IDP_dMRI_ProbtrackX_MD_cgh_r	1.076	2.934	5.86E-06	5.08E-04
dMRI_MD_precuneousR	1.439	4.216	6.99E-06	5.08E-04
dMRI_PSMD	1.157	3.182	4.12E-06	5.08E-04
FS_aparc.DKTatlas_rh_volume_medialorbitofrontal.	-0.677	0.508	1.14E-04	5.18E-04
FS_aparc.DKTatlas_rh_volume_superiortemporal.	-0.709	0.492	1.28E-04	5.74E-04
FS_HippSubfield_rh_volume_CA4.head.	-0.637	0.529	1.30E-04	5.78E-04
FS_HippSubfield_lh_volume_presubiculum.body.	-0.603	0.547	1.36E-04	5.96E-04
WMH_total	0.685	1.984	4.80E-05	6.12E-04
T1_GM_parcellation_L_Inf_Temp_Gyr_temporoOcc_part_vol_LM	-0.622	0.537	1.42E-04	6.15E-04
T1_GM_parcellation_R_Temp_Fusiform_post_vol_LM	-0.662	0.516	1.43E-04	6.15E-04
rfMRI_d100_NodeAmplitudes_44	-1.182	0.307	6.56E-07	7.58E-04
rfMRI_d25_NodeAmplitudes_6	-1.088	0.337	6.23E-07	7.58E-04
FS_aparc.DKTatlas_lh_volume_entorhinal.	-0.588	0.556	2.08E-04	8.70E-04
T1_GM_parcellation_L_Inf_Front_Gyr_pars_triangularis_vol_LM	-0.560	0.571	2.11E-04	8.70E-04
T1_GM_parcellation_R_Sup_Temp_Gyr_ant_vol_LM	-0.636	0.530	2.10E-04	8.70E-04
T1_GM_parcellation_R_Postcentral_Gyr_vol_LM	-0.639	0.528	2.07E-04	8.70E-04
T1_GM_parcellation_L_Supramarginal_Gyr_post_vol_LM	-0.602	0.548	2.24E-04	8.93E-04
T1_GM_parcellation_R_Lateral_Occ_Sup_vol_LM	-0.612	0.542	2.24E-04	8.93E-04
T1_GM_parcellation_R_Planum_Polare_vol_LM	-0.722	0.486	2.22E-04	8.93E-04
T2_FLAIR_WMHvol_Posterior_thalamic_radiation_L	0.625	1.869	9.15E-05	9.33E-04
IDP_T1_FIRST_left_accumbens_volume	-0.588	0.555	2.44E-04	9.63E-04
T2_FLAIR_WMHvol_Superior_fronto.occipital_fasciculus_R	0.710	2.035	1.18E-04	1.00E-03
IDP_T1_FIRST_right_accumbens_volume	-0.577	0.561	2.65E-04	1.03E-03
T1_GM_parcellation_L_Subcallosal_vol_LM	-0.713	0.490	2.67E-04	1.03E-03
T1_GMvol_occ	-0.722	0.486	2.99E-04	1.15E-03

FS_HippSubfield_lh_volume_CA1.body.	-0.556	0.573	3.24E-04	1.23E-03
T1_GM_parcellation_R_Angular_Gyr_vol_LM	-0.552	0.576	3.28E-04	1.23E-03
T1_GM_parcellation_R_Planum_Tempe_vol_LM	-0.646	0.524	3.31E-04	1.23E-03
T1_GM_parcellation_L_Front_Medial_vol_LM	-0.573	0.564	3.73E-04	1.38E-03
rfMRI_d100_NodeAmplitudes_21	-1.036	0.355	2.19E-06	1.52E-03
rfMRI_d25_NodeAmplitudes_13	-1.049	0.350	1.92E-06	1.52E-03
T1_GM_parcellation_L_Lateral_Occ_Inf_vol_LM	-0.568	0.567	4.18E-04	1.53E-03
FS_aparc.DKTatlas_rh_volume_inferiorparietal.	-0.618	0.539	4.50E-04	1.63E-03
T1_GM_parcellation_L_Caudate_vol_LM	-0.653	0.521	5.05E-04	1.81E-03
rfMRI_d100_NodeAmplitudes_31	-0.980	0.375	3.24E-06	1.87E-03
FS_HippSubfield_lh_volume_FS_Hippocampal.tail.	-0.545	0.580	5.49E-04	1.94E-03
T1_GM_parcellation_R_Front_Operculum_vol_LM	-0.562	0.570	5.47E-04	1.94E-03
FS_HippSubfield_rh_volume_presubiculum.head.	-0.590	0.554	5.72E-04	2.00E-03
T1_GM_parcellation_L_Parietal_Operculum_vol_LM	-0.537	0.584	5.98E-04	2.07E-03
T2_FLAIR_WMHvol_Anterior_corona_radiata_R	0.593	1.810	2.89E-04	2.10E-03
FS_aparc.DKTatlas_lh_volume_insula.	-0.610	0.544	6.42E-04	2.21E-03
FS_HippSubfield_lh_volume_molecular.layer.HP.head.	-0.556	0.573	6.83E-04	2.33E-03
FS_HippSubfield_rh_volume_FS_Hippocampal.tail.	-0.526	0.591	6.89E-04	2.33E-03
T1_GM_parcellation_L_Supramarginal_Gyr_ant_vol_LM	-0.535	0.586	7.07E-04	2.37E-03
FS_aparc.DKTatlas_lh_volume_superiorparietal.	-0.584	0.558	7.38E-04	2.45E-03
FS_HippSubfield_rh_volume_CA1.body.	-0.513	0.599	7.82E-04	2.57E-03
rfMRI_d100_NodeAmplitudes_25	-0.928	0.395	5.22E-06	2.59E-03
FS_aparc.DKTatlas_lh_thickness_medialorbitofrontal.	-0.510	0.600	7.97E-04	2.59E-03
FS_aparc.DKTatlas_rh_thickness_middletemporal.	-0.519	0.595	7.99E-04	2.59E-03
FS_aparc.DKTatlas_lh_thickness_paraFS_Hippocampal.	-0.504	0.604	8.56E-04	2.75E-03
IDP_dMRI_ProbtrackX_ISOVF_cgh_r	0.925	2.523	5.19E-05	2.83E-03
IDP_T1_FIRST_left_putamen_volume	-0.552	0.576	9.30E-04	2.96E-03
T1_GM_parcellation_R_Supramarginal_Gyr_post_vol_LM	-0.532	0.588	9.73E-04	3.07E-03
FS_aparc.DKTatlas_lh_volume_paraFS_Hippocampal.	-0.488	0.614	9.99E-04	3.13E-03
rfMRI_d100_NodeAmplitudes_7	-0.922	0.398	8.30E-06	3.20E-03
rfMRI_d100_NodeAmplitudes_45	-0.957	0.384	7.91E-06	3.20E-03
rfMRI_d100_partialcorr_442	0.934	2.546	9.37E-06	3.25E-03
T1_GM_parcellation_L_Planum_Polare_vol_LM	-0.588	0.555	1.05E-03	3.26E-03
IDP_dMRI_ProbtrackX_MD_cgh_l	0.771	2.162	9.40E-05	3.42E-03
IDP_dMRI_ProbtrackX_MO_fmi	-0.792	0.453	8.01E-05	3.42E-03
T2_FLAIR_WMHvol_Genu_of_corpus_callosum	0.552	1.737	5.77E-04	3.46E-03
T2_FLAIR_WMHvol_Superior_corona_radiata_R	0.585	1.795	6.79E-04	3.46E-03
T2_FLAIR_WMHvol_Superior_corona_radiata_L	0.554	1.740	6.34E-04	3.46E-03
IDP_T1_FIRST_right_putamen_volume	-0.532	0.588	1.15E-03	3.54E-03
FS_aparc.DKTatlas_lh_thickness_insula.	-0.486	0.615	1.19E-03	3.64E-03

T1_GM_parcellation_L_Mid_Temp_Gyr_temporoOcc_part_vol_LM	-0.486	0.615	1.32E-03	4.01E-03
T1_SIENAX_brain.unnorm_vol_LM	-0.948	0.388	1.36E-03	4.10E-03
FS_aparc.DKTatlas_rh_thickness_insula.	-0.493	0.611	1.37E-03	4.11E-03
IDP_dMRI_ProbtrackX_MD_ptr_l	0.793	2.210	1.39E-04	4.34E-03
T2_FLAIR_WMHvol_Posterior_thalamic_radiation_R	0.535	1.708	9.69E-04	4.35E-03
T2_FLAIR_WMHvol_Cingulum_cingulate_gyrus_L	0.674	1.962	1.02E-03	4.35E-03
FS_aparc.DKTatlas_lh_thickness_pericalcarine.	0.458	1.580	1.47E-03	4.36E-03
FS_aparc.DKTatlas_rh_volume_entorhinal.	-0.524	0.592	1.49E-03	4.39E-03
FS_aparc.DKTatlas_rh_thickness_medialorbitofrontal.	-0.500	0.607	1.52E-03	4.44E-03
T1_GM_parcellation_R_Sup_Parietal_Lobule_vol_LM	-0.471	0.624	1.69E-03	4.92E-03
T1_GM_parcellation_R_Sup_Front_Gyr_vol_LM	-0.529	0.589	1.77E-03	5.11E-03
FS_aparc.DKTatlas_lh_volume_fusiform.	-0.542	0.581	1.85E-03	5.28E-03
T2_FLAIR_WMHvol_External_capsule_R	0.533	1.704	1.62E-03	6.36E-03
T1_GM_parcellation_R_Mid_Temp_Gyr_temporoOcc_part_vol_LM	-0.483	0.617	2.26E-03	6.43E-03
T1_GM_parcellation_R_Front_Medial_vol_LM	-0.486	0.615	2.28E-03	6.43E-03
rfMRI_d100_NodeAmplitudes_19	-0.895	0.409	2.05E-05	6.47E-03
dMRI_MO_supramargR	-0.832	0.435	2.57E-04	7.02E-03
FS_HippSubfield_lh_volume_molecular.layer.HP.body.	-0.453	0.636	2.61E-03	7.31E-03
rfMRI_d100_NodeAmplitudes_37	-0.858	0.424	2.75E-05	7.94E-03
FS_aparc.DKTatlas_rh_thickness_paraFS_Hippocampal.	-0.450	0.638	2.88E-03	8.02E-03
FS_aparc.DKTatlas_lh_volume_medialorbitofrontal.	-0.506	0.603	2.91E-03	8.04E-03
FS_aparc.DKTatlas_lh_thickness_inferiorparietal.	-0.445	0.641	3.01E-03	8.24E-03
T1_GM_parcellation_L_Pallidum_vol_LM	0.456	1.577	3.04E-03	8.27E-03
T2_FLAIR_WMHvol_Retrolenticular_part_of_internal_capsule_R	0.458	1.581	2.27E-03	8.28E-03
FS_HippSubfield_rh_volume_subiculum.head.	-0.486	0.615	3.17E-03	8.51E-03
T1_GM_parcellation_R_Inf_Front_Gyr_pars_triangularis_vol_LM	-0.446	0.640	3.15E-03	8.51E-03
IDP_dMRI_ProbtrackX_ISOVF_unc_r	0.726	2.067	3.53E-04	8.56E-03
FS_aparc.DKTatlas_rh_volume_inferiortemporal.	-0.507	0.603	3.32E-03	8.87E-03
FS_HippSubfield_lh_volume_HATA.	-0.447	0.640	3.37E-03	8.87E-03
FS_aparc.DKTatlas_rh_area_middletemporal.	-0.547	0.579	3.36E-03	8.87E-03
FS_aparc.DKTatlas_lh_volume_superiorfrontal.	-0.518	0.596	3.56E-03	9.28E-03
FS_aparc.DKTatlas_rh_volume_insula.	-0.513	0.599	3.57E-03	9.28E-03
IDP_dMRI_ProbtrackX_ISOVF_cgh_l	0.662	1.938	4.68E-04	9.28E-03
dMRI_MO_R_amyg	-0.771	0.462	4.39E-04	9.28E-03
T1_GM_parcellation_L_Inf_Front_Gyr_pars_opercularis_vol_LM	-0.457	0.633	3.66E-03	9.44E-03
T2_FLAIR_WMHvol_Posterior_corona_radiata_L	0.460	1.584	2.88E-03	9.80E-03
T2_FLAIR_WMHvol_Superior_longitudinal_fasciculus_L	0.473	1.604	3.15E-03	1.00E-02
IDP_dMRI_ProbtrackX_ICVF_fma	-0.672	0.510	5.57E-04	1.01E-02
FS_aparc.DKTatlas_lh_volume_parstriangularis.	-0.457	0.633	4.21E-03	1.08E-02

T1_GM_parcellation_L_Occ_Pole_vol_LM	-0.456	0.634	4.23E-03	1.08E-02
FS_aparc.DKTatlas_rh_thickness_inferiortemporal.	-0.448	0.639	4.33E-03	1.09E-02
FS_aparc.DKTatlas_rh_volume_fusiform.	-0.480	0.619	4.34E-03	1.09E-02
rfMRI_d100_NodeAmplitudes_13	-0.845	0.429	4.19E-05	1.12E-02
IDP_T1_FIRST_right_amygdala_volume	-0.420	0.657	4.84E-03	1.19E-02
FS_aparc.DKTatlas_lh_area_superiorparietal.	-0.488	0.614	4.91E-03	1.19E-02
FS_aparc.DKTatlas_rh_thickness_pericalcarine.	0.413	1.512	4.86E-03	1.19E-02
T1_GM_parcellation_R_Mid_Front_Gyr_vol_LM	-0.488	0.614	4.82E-03	1.19E-02
T1_GM_parcellation_R_Parahippocampal_Gyr_post_vol_LM	-0.444	0.641	4.88E-03	1.19E-02
T1_NBMvol_L	-0.462	0.630	4.79E-03	1.19E-02
T1_NBMvol_R	-0.440	0.644	4.96E-03	1.20E-02
T2_FLAIR_WMHvol_Posterior_limb_of_internal_capsule_R	0.559	1.749	4.37E-03	1.24E-02
T2_FLAIR_WMHvol_Tapetum_L	0.424	1.528	4.13E-03	1.24E-02
IDP_dMRI_ProbtrackX_ISOVF_ptr_l	0.671	1.957	7.51E-04	1.26E-02
T1_GM_parcellation_L_Parahippocampal_Gyr_post_vol_LM	-0.439	0.645	5.33E-03	1.28E-02
T2_FLAIR_WMHvol_Retrolenticular_part_of_internal_capsule_L	0.505	1.657	4.76E-03	1.28E-02
T2_FLAIR_WMHvol_Posterior_corona_radiata_R	0.422	1.525	5.68E-03	1.45E-02
IDP_dMRI_ProbtrackX_MD_atr_r	0.668	1.951	1.34E-03	1.46E-02
IDP_dMRI_ProbtrackX_MD_ifo_l	0.689	1.991	1.02E-03	1.46E-02
IDP_dMRI_ProbtrackX_MD_ilf_l	0.652	1.919	1.05E-03	1.46E-02
IDP_dMRI_ProbtrackX_ICVF_ilf_l	-0.631	0.532	1.25E-03	1.46E-02
IDP_dMRI_ProbtrackX_ICVF_ptr_l	-0.626	0.534	1.22E-03	1.46E-02
IDP_dMRI_ProbtrackX_ICVF_ptr_r	-0.641	0.527	1.33E-03	1.46E-02
dMRI_tractvol_fma	-0.814	0.443	1.12E-03	1.46E-02
FS_aparc.DKTatlas_rh_volume_paraFS_Hippocampal.	-0.422	0.656	6.27E-03	1.49E-02
FS_aparc.DKTatlas_rh_volume_superiorfrontal.	-0.535	0.586	6.36E-03	1.50E-02
T1_GM_parcellation_L_Precentral_Gyr_vol_LM	-0.461	0.631	6.53E-03	1.54E-02
IDP_dMRI_ProbtrackX_MD_ilf_r	0.630	1.878	1.57E-03	1.56E-02
IDP_dMRI_ProbtrackX_ICVF_ilf_r	-0.617	0.540	1.57E-03	1.56E-02
IDP_dMRI_ProbtrackX_MD_str_r	0.635	1.886	1.66E-03	1.57E-02
T1_GM_parcellation_R_Inf_Front_Gyr_pars_opercularis_vol_LM	-0.406	0.666	7.16E-03	1.67E-02
IDP_dMRI_ProbtrackX_MD_ifo_r	0.635	1.886	1.93E-03	1.69E-02
IDP_dMRI_ProbtrackX_ICVF_cgh_r	-0.634	0.531	1.89E-03	1.69E-02
FS_aparc.DKTatlas_lh_area_inferiortemporal.	-0.471	0.625	7.35E-03	1.71E-02
IDP_dMRI_ProbtrackX_OD_str_l	-0.588	0.556	2.05E-03	1.72E-02
IDP_dMRI_ProbtrackX_FA_cgh_l	-0.595	0.552	2.36E-03	1.72E-02
IDP_dMRI_ProbtrackX_FA_ptr_l	-0.581	0.560	2.53E-03	1.72E-02
IDP_dMRI_ProbtrackX_MD_ptr_r	0.598	1.819	2.24E-03	1.72E-02
IDP_dMRI_ProbtrackX_MD_slf_l	0.586	1.797	2.76E-03	1.72E-02

IDP_dMRI_ProbtrackX_MD_slf_r	0.593	1.809	2.63E-03	1.72E-02
IDP_dMRI_ProbtrackX_ICVF_slf_r	-0.580	0.560	2.71E-03	1.72E-02
IDP_dMRI_ProbtrackX_OD_str_r	-0.573	0.564	2.36E-03	1.72E-02
dMRI_MD_parahippL	0.681	1.977	2.58E-03	1.72E-02
dMRI_tractvol_cst_l	0.714	2.041	2.54E-03	1.72E-02
T2_FLAIR_WMHvol_Tapetum_R	0.397	1.487	7.23E-03	1.76E-02
FS_HippSubfield_rh_volume_molecular.layer.HP.head.	-0.411	0.663	7.92E-03	1.82E-02
T1_GM_parcellation_Brain.Stem_vol_LM	0.420	1.521	7.88E-03	1.82E-02
rfMRI_d100_NodeAmplitudes_11	-0.773	0.462	9.61E-05	1.83E-02
rfMRI_d100_partialcorr_238	0.797	2.219	8.73E-05	1.83E-02
rfMRI_d100_partialcorr_360	-0.828	0.437	7.46E-05	1.83E-02
rfMRI_d25_NodeAmplitudes_1	-0.752	0.471	1.00E-04	1.83E-02
rfMRI_d25_NodeAmplitudes_14	-0.786	0.456	9.36E-05	1.83E-02
rfMRI_d25_fullcorr_105	0.761	2.141	9.86E-05	1.83E-02
IDP_dMRI_ProbtrackX_OD_slf_l	-0.569	0.566	3.17E-03	1.92E-02
FS_aparc.DKTatlas_lh_volume_parsopercularis.	-0.430	0.651	8.70E-03	1.99E-02
dMRI_MD_precuneousL	0.671	1.957	3.37E-03	1.99E-02
IDP_dMRI_ProbtrackX_ICVF_slf_l	-0.559	0.572	3.56E-03	2.04E-02
IDP_T1_FIRST_left_amygdala_volume	-0.372	0.689	9.16E-03	2.08E-02
IDP_dMRI_ProbtrackX_MD_unc_r	0.570	1.768	3.81E-03	2.12E-02
IDP_dMRI_ProbtrackX_ISOVF_unc_l	0.571	1.770	3.89E-03	2.12E-02
dMRI_tractvol_ml_l	0.744	2.104	4.07E-03	2.16E-02
IDP_dMRI_ProbtrackX_MD_cst_r	0.543	1.721	4.37E-03	2.27E-02
T2_FLAIR_WMHvol_Anterior_limb_of_internal_capsule_R	0.444	1.559	1.05E-02	2.28E-02
T2_FLAIR_WMHvol_Posterior_limb_of_internal_capsule_L	0.489	1.631	1.04E-02	2.28E-02
T2_FLAIR_WMHvol_Superior_fronto.occipital_fasciculus_L	0.415	1.515	1.07E-02	2.28E-02
dMRI_MD_R_amyg	0.652	1.919	4.59E-03	2.33E-02
FS_aparc.DKTatlas_lh_thickness_supramarginal.	-0.391	0.676	1.07E-02	2.41E-02
T2_FLAIR_WMHvol_Anterior_corona_radiata_L	0.394	1.483	1.19E-02	2.43E-02
IDP_dMRI_ProbtrackX_ICVF_cgh_l	-0.554	0.574	5.07E-03	2.51E-02
IDP_dMRI_ProbtrackX_MO_cgh_l	-0.551	0.577	5.44E-03	2.60E-02
IDP_dMRI_ProbtrackX_ICVF_ifo_l	-0.542	0.582	5.61E-03	2.60E-02
IDP_dMRI_ProbtrackX_ICVF_ifo_r	-0.554	0.575	5.56E-03	2.60E-02
T1_GM_parcellation_R_Parietal_Operculum_vol_LM	-0.412	0.662	1.18E-02	2.65E-02
rfMRI_d100_partialcorr_961	-0.755	0.470	1.55E-04	2.68E-02
FS_aparc.DKTatlas_rh_area_fusiform.	-0.457	0.633	1.22E-02	2.70E-02
T1_GM_parcellation_R_Occ_Fusiform_Gyr_vol_LM	-0.396	0.673	1.22E-02	2.70E-02
IDP_dMRI_ProbtrackX_ICVF_ar_r	-0.513	0.599	5.99E-03	2.72E-02
rfMRI_d100_NodeAmplitudes_24	-0.740	0.477	1.84E-04	3.03E-02
rfMRI_d100_NodeAmplitudes_12	-0.757	0.469	2.02E-04	3.04E-02

rfMRI_d100_fullcorr_442	0.697	2.007	1.94E-04	3.04E-02
T1_GM_parcellation_R_Heschl_Gyr_vol_LM	-0.407	0.666	1.38E-02	3.05E-02
rfMRI_d100_fullcorr_135	0.742	2.100	2.23E-04	3.21E-02
IDP_dMRI_ProbtrackX_FA_fmi	-0.527	0.591	7.33E-03	3.23E-02
IDP_dMRI_ProbtrackX_ISOVF_fmi	0.491	1.634	7.56E-03	3.23E-02
dMRI_MO_supramargL	-0.570	0.565	7.57E-03	3.23E-02
rfMRI_d100_fullcorr_1095	-0.729	0.483	2.35E-04	3.26E-02
IDP_dMRI_ProbtrackX_MD_unc_l	0.555	1.742	7.96E-03	3.27E-02
IDP_dMRI_ProbtrackX_OD_ar_l	-0.496	0.609	7.92E-03	3.27E-02
FS_aparc.DKTatlas_rh_thickness_isthmuscingulate.	-0.363	0.696	1.51E-02	3.30E-02
FS_aparc.DKTatlas_rh_area_inferiorparietal.	-0.439	0.644	1.52E-02	3.32E-02
IDP_dMRI_ProbtrackX_MO_atr_r	-0.484	0.617	8.23E-03	3.32E-02
